# Mucosal vaccination with hemagglutinin stalk provides broad protection against influenza virus subtypes in mice

**DOI:** 10.1172/JCI203675

**Published:** 2026-11-16

**Authors:** Weizhong Li, Arunraj M. Rajendrakumar, ZiZhen Miao, Bibek Dhakal, Bogdan M. Domrachev, Tao Wang, Qiuqi Kan, Qiaofeng Wan, Hongquan Wan, Wenbin Tuo, Florian Krammer, Zhiping Ye, Xiaoping Zhu

**Affiliations:** 1 Division of Immunology, Virginia-Maryland College of Veterinary Medicine, University of Maryland, College Park, Maryland, USA.; 2 Animal Parasitic Diseases Laboratory, Agricultural Research Service, United States Department of Agriculture, Beltsville, Maryland, USA.; 3 Division of Viral Products, Center for Biologics Evaluation and Research, Food and Drug Administration, Silver Spring, Maryland, USA.; 4 Department of Microbiology, Icahn School of Medicine at Mount Sinai, New York, New York, USA.

**Keywords:** Immunology, Infectious disease, Virology, Influenza, Vaccines

## Abstract

Influenza viruses continually evolve through antigenic drift and shift, spreading via respiratory droplets and aerosols. Developing a universal nasal vaccine is essential for preventing seasonal and pandemic infections by inducing long-lasting immunity in the respiratory tract. The neonatal Fc receptor (FcRn) transfers IgG Abs across the epithelial barrier. This study investigates whether the i.n. delivery of the conserved Stalk antigen of HA, utilizing the FcRn, can stimulate protective immune responses against various influenza virus subtypes. We engineered a monomeric IgG Fc fused to a modified HA Stalk in its prefusion form. Mice i.n. immunized with Stalk-Fc exhibited significant protection against infections from H1N1, H5N1, H6N2, and H9N2 compared with groups of control mice. The immunized mice produced long-lasting HA Stalk–specific IgA and IgG Abs in nasal washes, bronchoalveolar lavage, and blood, along with tissue-resident memory T cells in the lungs and long-lived plasma cells in the bone marrow. This led to notably lower viral replication in the nasal turbinate and lungs. The Stalk-specific Abs demonstrated strong cross-reactivity against different influenza subtypes through neutralization, Ab- or complement-dependent cellular cytotoxicity, or phagocytosis. These results indicate that FcRn efficiently transports HA Stalk-Fc across the respiratory epithelial barrier, supporting the development of a universal influenza nasal vaccine.

## Abstract 2

Devekoping a universal flu nasal vaccine to provide broad protection against multiple strains of influenza virus infection and transmission

## Graphical abstract

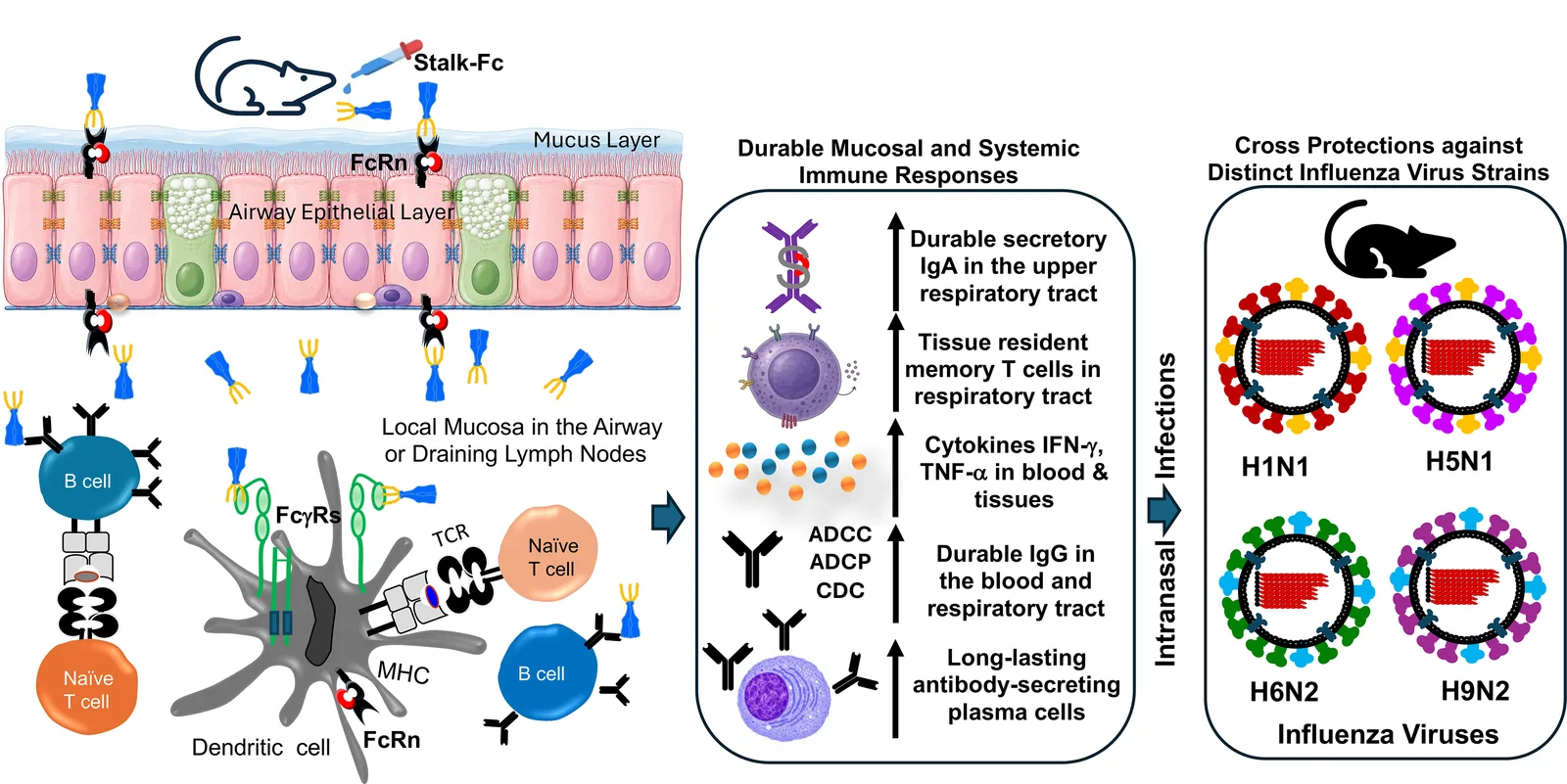

## Introduction

Influenza A virus is an enveloped RNA virus with an 8-segmented genome. The virus envelope contains 3 proteins: HA, neuraminidase (NA), and matrix protein 2. New strains or subtypes of the influenza virus can emerge through antigenic mutations, genetic mixing of viruses, or both during infections (1). Annual seasonal influenza vaccines are available, but frequent antigenic drift results in circulating virus strains differing from the vaccine strains and significantly reducing vaccine effectiveness. Additionally, seasonal vaccines are unlikely to protect against a newly emerging strain of the influenza virus. To address this, efforts have been made to develop universal influenza vaccines that provide strong, long-lasting protection against multiple virus subtypes (2).

Since influenza virus infection and transmission occur through airborne particles, respiratory droplets, and contact (3), the upper and lower respiratory tracts are common sites of exposure. There is an urgent need to develop a safe and effective universal influenza vaccine, including a nasal spray option, to prevent initial infection and the spread of the virus. Most broad-protective or universal influenza vaccines in development are designed for delivery via muscle or skin routes. However, these methods do not always generate effective mucosal immune responses (4–6), such as secretory IgA (sIgA) and tissue-resident memory (TRM) T cells (7–9) in the respiratory tract, which are essential for defending against respiratory infections. Therefore, an ideal universal influenza vaccine should deliver the antigens directly through the respiratory route, mimicking natural infections and promoting beneficial local immunity. Our current ability to deliver universal influenza vaccine antigens safely and effectively across the respiratory mucosal barrier remains limited.

The respiratory tract is lined with a layer of simple or pseudostratified columnar epithelial cells. These cells have 2 distinct plasma membrane domains, apical and basolateral, separated by tight junctions. The neonatal Fc receptor (FcRn) present in these cells helps transfer naturally occurring IgG Abs across the epithelium (10). This transfer process, known as IgG transcytosis, is crucial for the body’s defense at mucosal surfaces. FcRn extends the lifespan of IgG in circulation or tissues by protecting it from degradation in lysosomes (11). Once IgG binds to FcRn, it undergoes a nondegradative transport pathway within epithelial cells. It is eventually released into the lumen, submucosa, or cell surfaces to reenter the circulation at physiological pH.

Immunity to natural influenza infection mainly relies on anti-HA Abs. The influenza HA protein has a membrane-distal globular head and a membrane-proximal Stalk domain. The HA head facilitates the virus’s binding to cell surface receptors containing sialic acid residues, while the Stalk mediates the fusion of viral and endosomal membranes during entry, a critical step for the release of the viral genome into the cell. Abs induced by HA mainly target the head domain, and these neutralizing Abs are crucial for preventing viral entry into cells and stopping infection. The HA head exhibits high structural flexibility and continuously undergoes antigenic changes. In contrast, the HA Stalk is conserved across different influenza strains (12–14). The Abs targeting the HA Stalk can protect against multiple influenza virus strains (15–21); therefore, the HA Stalk has been recognized as a major target for eliciting broader, cross-reactive immunity.

Our previous study demonstrated that FcRn effectively delivers the trimeric influenza HA antigen in the respiratory tract, triggering strong mucosal and systemic immunity against lethal influenza virus infection (22). In this study, we further explored FcRn’s ability to transport the HA Stalk antigen fused to a monomeric Fc region of IgG across the respiratory epithelial barrier. We further evaluated the protective immune responses and mechanisms associated with this delivery method for mucosal vaccination. Our findings indicate that FcRn-mediated i.n. delivery of the HA Stalk results in elevated levels of long-lasting Ab and T cell responses, including TRM T cells in the lungs, which offer broad protection against infections from different influenza virus subtypes in mice. These results demonstrate that FcRn-mediated i.n. delivery of HA Stalk antigens could be an effective universal influenza mucosal vaccine against both seasonal and pandemic influenza viruses.

## Results

### Expression and characterization of HA Stalk-Fc fusion proteins.

The HA Stalk is a promising target for eliciting broadly neutralizing or cross-reactive Abs (23). However, the immunogenicity of HA Stalk is believed to be masked by the highly immunogenic HA head (23, 24). To induce broad and high levels of Stalk-reactive Abs, several studies have designed and expressed stable headless HA Stalk domains using structure-based rational approaches (18, 19, 25–28), which include the HA2 domain and partial regions of HA1 that contribute to Stalk folding.

To generate a stabilized, trimeric HA Stalk protein, the HA (GenBank AAP34324.1) of influenza virus A/New Caledonia/20/1999 (H1N1) was used as a backbone. Several changes were made to remove the head and stabilize the Stalk as a prefusion conformation. First, 33–310 aa (mature HA protein) were deleted and replaced with a GSG linker. Second, the SIQS sequence before the HA1-HA2 cleavage site (R326/G327) was substituted with the QRET sequence. Third, 386–418 aa were replaced with the GSGGSG linker. Fourth, to stabilize the HA Stalk conformation under low-pH conditions, several mutations (K377M, Y420D, N421L, and E429L) were introduced. To promote trimer formation, the bacteriophage T4 foldon domain (PDB ID 1RFO) was added to the C-terminus of the Stalk. The HA Stalk was expressed as a His-tagged protein (Stalk, Figure 1A).

To target FcRn, we fused the modified HA Stalk and foldon domain with the Fc portion of mouse IgG2a, creating Stalk-Fc. Mouse IgG2a Fc, which is like human IgG1 Fc, was chosen due to its high affinity for activating FcγRI (29). Since IgG Fc usually forms a disulfide-bonded dimer, we made a monomeric Fc by replacing cysteine residues at positions 224, 227, and 229 with serine in the hinge region to remove the disulfide bonds (Stalk-Fc/wt, Figure 1A). We also generated a mutant Fc (Stalk-Fc/mut, Figure 1A) that cannot bind FcRn by making HQ/AD mutations at positions 310/311and HN/AQ mutations at positions 433/434 (30). In both the wild-type (wt) and mutant (mut) Fc, the C1q-binding motif was removed to eliminate C1q binding (31). As a result, we fused either the monomeric IgG Fc/wt or Fc/mut with HA Stalk-foldon (Supplemental Table 1; supplemental material available online with this article; https://doi.org/10.1172/JCI203675DS1). The soluble Stalk-Fc or Stalk proteins were secreted from the transfected CHO cells and purified using affinity columns (Figure 1B). The Stalk-Fc/wt, Stalk-Fc/mut, and Stalk proteins were verified by Western blots using Abs against mouse IgG, HA, or His tag (Supplemental Figure 1). Importantly, Stalk-Fc/wt and Stalk-Fc/mut were expressed as monomers under both nonreducing (Supplemental Figure 1, left) and reducing conditions (Supplemental Figure 1, middle), confirming that the removal of disulfide bonds prevented Fc dimerization. The trimeric structure of the Stalk-Fc/wt or Spike was predicted by AlphaFold 3, showing the independent folding of the HA Stalk and Fc domains.

FcRn binds IgG in a pH-dependent manner (11). To test whether the Stalk-Fc/wt protein binds to FcRn, FcγRI, or C1q, we performed an ELISA. The results showed that, like mouse IgG, Stalk-Fc/wt bound to FcRn at pH 6.0 but not at pH 7.4 (Figure 1C). In contrast, both Stalk-Fc/mut and the standalone Stalk protein failed to bind FcRn at pH 6.0. Stalk-Fc/wt and Stalk-Fc/mut bound to the high-affinity receptor FcγRI (CD64) (Figure 1D). Surface plasmon resonance assay further confirmed that the affinity constant (*K_D_*) for Stalk-Fc binding to immobilized FcRn or FcγRI was 8.73 × 10^–8^ M (Supplemental Figure 2, left) or 1.67 × 10^–7^ M (Supplemental Figure 2, right), respectively. Unlike IgG, Stalk-Fc/wt, Stalk-Fc/mut, and Stalk proteins did not react with C1q, confirming the successful removal of the C1q binding site in the Fc portion of Stalk-Fc (Figure 1E). Taken together, these results indicate that the Stalk-Fc/wt protein maintains the structural integrity necessary for interaction with FcRn or FcγRI, but not with C1q protein, enabling Stalk-Fc to be nonlytic.

HA Stalk fused to an Fc fragment more effectively mimics the native HA Stalk structure by maintaining conformation-dependent B cell epitopes (32). We tested a panel of HA Stalk–specific mAbs known for their broad binding to conformational epitopes in the HA Stalk (33–36). Both Stalk-Fc fusion and Stalk were recognized by the mAbs KB2, CR6261, FI6v3, and 6F12 and a polyclonal Ab against Stalk in ELISAs (Figure 1F). However, the CR8020 mAb showed low-affinity binding (Supplemental Figure 3); this is expected because CR8020 mainly interacts with the HA Stalk from group 2 influenza viruses (37). SARS-CoV-2 Spike protein (38) was used as a negative control, demonstrating specific Ab binding. Results of Ab binding are summarized in a heatmap and presented as EC_50_ (log ng/mL) (Figure 1G).

The stability of HA Stalk-Fc was tested under various conditions, including repeated freeze-thaw cycles (5 or 10 times), heating at 55°C for 30 min or 1 h, incubating at 37°C for 1 or 2 days, and storage at room temperature for 4 or 6 days (Supplemental Figure 4A). The Stalk-Fc protein exhibited significant stability, as demonstrated by a Coomassie blue–stained SDS-PAGE gel. A minor band at 37 kDa was present on the SDS-PAGE gel; however, this band was undetectable by either anti-HA or anti-mouse IgG Abs in a Western blot (Supplemental Figure 1). Additionally, in comparison with the untreated protein, the treated Stalk-Fc protein under the above conditions maintained the ability to bind to FcRn; FcγRI; anti-mouse IgG2a Ab; mAbs KB2, CR6261, FI6v3, and 6F12; and a polyclonal Ab against the Stalk in ELISA (Supplemental Figure 4, B–I). Overall, these findings indicate that both the Fc and Stalk portions of the Stalk-Fc fusion protein retain their ability to interact with FcRn and FcγRI under rigorous conditions while preserving their conformational B cell epitopes.

### FcRn-dependent delivery of Stalk-Fc/wt antigen across the epithelial barrier.

To determine whether Stalk-Fc/wt, but not Stalk-Fc/mut, fusion protein is transported across the epithelial barrier by FcRn, we used an MDCK^FcRn^ cell line to study the transport of Stalk-Fc fusion proteins. MDCK cells expressing rat FcRn and rat β2m have been shown to specifically transport murine IgG in vitro (39). When Stalk-Fc/wt was applied to the apical reservoir (Figure 2A), it was transported to the basolateral reservoir and detected by ELISA (Figure 2B). In contrast, Stalk-Fc/mut or Stalk alone were poorly transported across the MDCK^rFcRn+rb2m^ monolayer (Figure 2B), indicating that Stalk-Fc/wt was transported specifically by FcRn.

To further examine whether Stalk-Fc is transported across the nasal epithelial cells in vivo following i.n. administration, 10 μg of the AF647-labeled Stalk-Fc/wt, Stalk-Fc/mut, or 5.5 μg (equal molar number) of the Stalk protein was i.n. administered to mice; after 7 h, the nose was harvested, decalcified, and subjected to cryosection at the middle part of the nasal passages. The coronal planes were visualized under a confocal microscope (Figure 2B). As shown in Figure 2C, Stalk-Fc/wt was readily detected as punctate staining in the nasal epithelial tissue (arrows) of WT mice but not in that of FcRn-KO animals. Additionally, Stalk-Fc/mut or Stalk protein alone remained mainly on the apical surface of nasal epithelial cells, which was barely transported. Based on these results, we conclude that the Stalk-Fc/wt fusion protein was efficiently transported across the polarized epithelial cells lining the airway in an FcRn-dependent manner.

### FcRn-mediated nasal delivery significantly enhances Stalk-specific systemic and local immune responses.

To determine whether FcRn-dependent transport enhances the immunogenicity of the HA Stalk antigen, mice were i.n. immunized with 10 μg of Stalk-Fc, 5.5 μg Stalk (in equal molar amount), or PBS (Supplemental Table 2), followed by boosting with the same dose twice at 3-week intervals (Figure 3A). Using WT mice immunized with Stalk-Fc/mut and FcRn-KO mice immunized with Stalk-Fc/wt, we demonstrated the necessity of FcRn binding for enhancing immune responses. Using Stalk alone allowed us to assess the FcRn-independent effects in vivo. The inclusion of these control groups also removed potential concerns regarding mucosal leakage. A mucosal adjuvant, CpG (10 �g/mouse) or c-di-GMP (5 �g), was used to overcome potential mucosal tolerance (40, 41). Previous studies demonstrated that neither CpG nor c-di-GMP compromises the structural integrity of the respiratory epithelial barrier (41, 42).

Two weeks after the final boost (Figure 3A), significantly higher titers of Stalk-specific IgG in the sera were detected in the Stalk-Fc/wt–immunized WT mice compared with the Stalk-Fc/wt–immunized FcRn-KO mice or Stalk-, Stalk-Fc/mut–, and PBS-treated groups (Figure 3B). To show the breadth and cross-reactive potency of the Ab response induced by the Stalk-Fc/wt protein, we performed an ELISA by coating various HA proteins. Our results showed that the serum Abs from the Stalk-Fc/wt–immunized mice interacted with various HA proteins (HA1, HA2, HA5, HA9, and HA13) from group 1 influenza virus subtypes more extensively than the sera from PBS control mice (Figure 3C). In a competitive ELISA, the serum Abs from the Stalk-Fc/wt–immunized mice were found to significantly diminish the binding of the HA Stalk–specific mAbs, such as KB2, 6F12, and F16v3, by competitively binding to HA Stalk (Figure 3D). KB2 and 6F12 are effective against the infection of a wide range of group 1 influenza strains (34), and F16v3 effectively neutralizes both groups 1 and 2 influenza A viruses (43). Hence, the broad reactivity with different HAs and recognition of conserved neutralizing epitopes by the serum Abs indicate the potent efficacy of FcRn-mediated nasal delivery of the Stalk-Fc against various influenza A virus subtypes. Additionally, Stalk-Fc/wt proteins stimulated robust T cell immune responses in the spleens of WT mice, as demonstrated by IFN-γ–, TNF-α–, or IL-4–producing (Figure 3, E–G, respectively) cell responses, as shown by an enzyme-linked immunospot (ELISpot) assay, compared with other control groups after the boost.

Influenza virus initiates its infection in the airways (3). Therefore, a key objective of FcRn-mediated mucosal delivery of HA Stalk is to elicit stronger local immune responses at these specific anatomical sites. To this end, we measured the levels of Stalk-specific Abs in mucosal secretions following i.n. immunizations. Nasal wash and bronchoalveolar lavage (BAL) fluids were collected 2 weeks after the final boost and analyzed for Stalk-specific IgG and IgA using ELISA. The results showed significantly increased levels of Stalk-specific IgA and IgG in nasal washes (Figure 3H) and BAL (Figure 3I) from mice immunized with Stalk-Fc/wt compared with other control groups. During the initial assessment, it was noted that Stalk alone induced a barely detectable IgG and IgA immune response (Figure 3, B, H, and I) following i.n. administration. To verify its immunogenicity, we i.m. administered it along with aluminum adjuvant in mice. After boosting, Stalk alone induced a similar level of serum IgG compared with that of the Stalk-Fc/wt (Supplemental Figure 5). Overall, our study indicates that targeting HA Stalk to FcRn via an i.n. immunization strategy significantly enhances the levels of serum IgG Abs and mucosal IgA and IgG Abs.

### FcRn-mediated nasal delivery significantly induces TRM CD4^+^ and CD8^+^ T cells in the lung.

Memory CD4^+^ and CD8^+^ T cells play a crucial role in protecting against the influenza virus (44, 45). A key subset of these cells, known as TRM cells, are noncirculating and remain in the nasal and lung tissues, providing a rapid response upon reexposure to the influenza virus (7–9). In our study, we evaluated whether FcRn-mediated nasal delivery of HA Stalk-Fc/wt could induce the formation of TRM T cells in the lungs (Figure 4A). To distinguish circulating T cells from lung-resident T cells, PE-CD45 Ab was intravenously injected 5 min before the euthanasia of mice, and the cells were obtained immediately for analysis. The gating strategy for TRM T cells is depicted in Supplemental Figure 6. We found a significant number of CD4^+^CD69^+^CD103^+/–^ (Figure 4B) and CD8^+^CD69^+^CD103^+^ TRM cells (Figure 4C) in the lungs of mice that were i.n. immunized with Stalk-Fc/wt. Most importantly, we observed a significant increase in the proportion of both monofunctional and polyfunctional CD4^+^ TRM T cells producing IFN-γ, TNF-α, and IL-2 upon peptide pool stimulation (Figure 4D). This remarkable increase is proportional to the total number of CD4^+^ T cells detected in the lungs (Supplemental Figure 7A). We also noted that the functional CD8^+^ TRM T cells in the lungs of Stalk-Fc/wt–immunized mice exhibited similar responsiveness to HA peptides, as assessed by the percentages of monofunctional TRM cells producing IFN-γ, IL-2, and TNF-α, as well as cells producing both IFN-γ and TNF-α (Figure 4E). However, the total number of functional CD8^+^ TRM T cells detected in the lungs was relatively low, which is consistent with the fact that the total numbers of CD8^+^ T cells did not differ significantly across different groups (Supplemental Figure 7B). We did not include an i.m. control group because we did not observe significant increases in CD4^+^ or CD8^+^ TRM T cells in the lungs after i.m. immunization with HA-Fc (22) or Spike-Fc (38).

To further evaluate protection mediated by TRM T cells, which is independent of the protection conferred by circulating T cells, we treated mice with FTY720 prior to and during the infection (Figure 4A). This treatment sequesters circulating T cells within secondary lymphoid tissues, while TRM T cells remain in the lungs (7). After challenging mice with the rH5N1 virus, we found that most of the Stalk-Fc/wt–immunized mice treated with (90%) or without FTY720 (80%) were protected from virus infection, as indicated by minimal weight loss in both groups (Figure 4, F and G). In contrast, the PBS control mice treated with FTY720 experienced significant weight loss of up to 25% within 7–9 days following the viral challenge, ultimately resulting in 100% mortality (Figure 4, F and G). These results demonstrate that i.n. immunization with Stalk-Fc/wt protects mice from the morbidity and mortality associated with H5N1 infection, a protective efficacy independent of circulating lymphocytes. Therefore, our findings suggest that FcRn-mediated nasal delivery of Stalk-Fc can effectively induce protective TRM T cells in the lungs. Further investigation is warranted to determine the protective capacity of adoptively transferred TRM T cells.

### FcRn-mediated nasal immunization with Stalk-Fc provides increased protection against lethal challenges of various influenza virus subtypes.

To evaluate the effectiveness and breadth of the humoral and cellular immune responses generated by FcRn-mediated i.n. vaccination with Stalk-Fc/wt, we challenged immunized mice with a lethal dose (5 MLD_50_) of rH5N1, H1N1 (PR8), and rH6N2 viruses 3 weeks after the final boost (Figure 5A). All or most mice in the control groups, including Stalk-Fc/wt/KO, Stalk-Fc/mut, Stalk, and PBS, experienced significant weight loss, up to 25%, within 6–9 days following the viral challenges (Figure 5, B–D) and succumbed to death with a low survival rate ranging from 0% to 27.3% (Figure 5, E–G). In contrast, most WT mice immunized with Stalk-Fc/wt antigen did not exhibit severe body weight loss or other symptoms (Figure 5, B–D). Consequently, the Stalk-Fc/wt–immunized WT mice demonstrated substantial protective survival rates against rH5N1 (90%; Figure 5E), H1N1 (91%; Figure 5F), and rH6N2 (80%; Figure 5G) viruses. We also tested whether Stalk-Fc provides cross-protection against infection by group 2 H3N2 virus. Similar to the PBS control mice, the majority of Stalk-Fc/wt–immunized mice (4 out of 5) succumbed to H3N2 infection (Supplemental Figure 8).

We further evaluated virus replication in the nasal turbinate (Figure 5, H and J) and lungs (Figure 5, I and K) 4 days after the rH5N1 and H9N2 challenge. The results demonstrated a 1.5 to 2.5 log reduction of the live virus levels in the nasal turbinate or lungs of the Stalk-Fc/wt–immunized mice compared with the other control groups, which corresponds with the immune response results (Figure 3). Taken together, our data clearly indicate that FcRn-mediated nasal delivery of Stalk-Fc is essential for broad protection against infections from different influenza virus subtypes or strains.

Given that most people have received seasonal influenza vaccines or have been exposed to influenza virus infections, we further investigated whether i.n. immunization with Stalk-Fc could enhance preexisting immunity and provide additional protection. To establish preexisting immunity, we either i.m. immunized mice (*n* = 10) with HA1 and HA3 proteins, along with CpG and Alum adjuvants, or i.n. infected mice (*n* = 10) with a sublethal dose of the PR8 virus (Supplemental Figure 9A). The establishment of preexisting immunity was confirmed by detecting HA- or Stalk-specific IgG Abs in the sera of HA-immunized or PR8-infected mice (Supplemental Figure 9B). One month later, all mice with preexisting immunity were i.n. boosted with either 10 �g of Stalk-Fc (*n* = 5) or PBS (*n* = 5). Mice that received the Stalk-Fc boost developed significantly higher levels of HA-specific (Supplemental Figure 9C) or Stalk-specific (Supplemental Figure 9D) IgG compared with those of the PBS control mice. To further assess whether the i.n. boost with Stalk-Fc could reduce viral replication, we challenged the mice with a lethal dose of the rH5N1 virus. The results showed that the single Stalk-Fc boost significantly reduced rH5N1 virus replication in the nasal turbinate (Supplemental Figure 9E) and lungs (Supplemental Figure 9F) at 4 days post infection compared with the PBS control mice. In conclusion, an i.n. boost with Stalk-Fc protein can significantly enhance the preexisting immune response and lower viral replication in mice.

### FcRn-mediated nasal delivery of Stalk-Fc induces strong memory immune responses.

A universal influenza mucosal vaccine is expected to induce a long-lasting immune memory response (46). We determined whether the nasal delivery of Stalk-Fc/wt via the FcRn pathway could induce a durable memory immune response lasting at least 10 months after the final boost (Figure 6A). As shown in Figure 6B, the highest levels of Stalk-specific serum IgG were detected in WT mice immunized with Stalk-Fc/wt compared with those of the control groups. Additionally, we observed significantly higher levels of Stalk-specific IgA and IgG in the nasal washes (Figure 6C) and BAL fluid (Figure 6D) from the Stalk-Fc/wt–immunized mice. Interestingly, we were still able to detect remarkably high levels of Stalk-specific IgG in the sera (Figure 6E), IgA in nasal washes (Supplemental Figure 10A), and IgA and IgG in BAL fluid (Supplemental Figure 10B) from the Stalk-Fc/wt–immunized mice 16 months after the final boost, but not from other control groups. Using ELISpot, we found a substantially high number of Stalk-specific Ab-secreting cells in the bone marrow of mice immunized with Stalk-Fc/wt antigen after 10-month (Figure 6F) or 16-month (Supplemental Figure 10, C and D) immunizations. The presence of long-lived plasma cells in the bone marrow niche likely contributes to the sustained high levels of Stalk-specific IgG in the blood (47). These results reveal that Stalk-specific B cell memory is sustained at a high level for at least 10 months after immunization.

To evaluate whether these memory immune responses offer protection, we challenged the mice with a lethal dosage of rH5N1 virus 6 months after the boost. Most mice in the control groups experienced a severe body weight drop within 4–9 days after the challenge and either succumbed to the infection or were euthanized after losing 25% of their initial body weight (Figure 6G). In contrast, mice immunized with Stalk-Fc/wt antigen showed significantly reduced disease severity, with a survival rate of 80% (Figure 6H). Overall, the mucosal delivery of Stalk-Fc via the FcRn pathway resulted in an effective memory immune response and protected against the lethal challenge of influenza viruses.

### Stalk-specific Ab-mediated neutralizing activity and inhibition of membrane fusion.

To explore the mechanisms of protection against lethal influenza virus infection in mice i.n. immunized with the Stalk-Fc antigen, we tested the immune sera using pseudotype microneutralization (pMN) assay. Typically, the pMN assay is more sensitive than the conventional MN assay (43, 48), especially for measuring HA Stalk–specific Abs. The pH5N1 virus, a pseudotyped lentiviral particle expressing HA5 and NA1 surface proteins of the H5N1 virus and harboring a luciferase reporter construct, was used. As shown in Figure 7A, the pooled Stalk-Fc/wt–immunized mouse sera exhibited powerful neutralization activity against pH5N1 virus (pMN titer = 960), while the sera from other control groups exhibited much less neutralization ability (pMN titer = 100 or <20), demonstrating that the HA Stalk-Fc/wt can induce Stalk-specific neutralizing Abs.

We next tested whether the serum Abs utilized the inhibitory mechanisms typically observed for Stalk-binding mAbs (43, 49). The HA0 precursor of influenza A virus HA is found on the surfaces of infected cells. When exposed to trypsin treatment and the low pH of the environment, HA0 undergoes a conformational change from its native form to a fusion-active form, leading to the formation of polykaryons (50). Indeed, Vero cells infected with H1N1 at pH 5.0 displayed polykaryon formation, whereas no polykaryons were visualized at pH 7.2 (Figure 7B, left). We further examined the effects of immune sera from Stalk-Fc–immunized mice on the fusion process of Vero cells infected with different influenza strains. Notably, the immune sera significantly inhibited polykaryon formation in Vero cells infected with H1N1 (Figure 7B, right, and Supplemental Figure 11). In contrast, sera from PBS control mice did not prevent this fusion. Additionally, the immune sera effectively blocked polykaryon formation in Vero cells infected with rH5N1, rH6N2, and rH9N2 (Figure 7, C–E) compared with the sera from PBS control mice. We expected that the sera from Stalk-Fc–immunized mice would not inhibit polykaryon formation in Vero cells infected with H3N2 virus (Figure 7F), as H3N2 belongs to group 2 influenza A virus. In a quantitative assay (Figure 7G), we calculated the fusion indexes of each group; the indexes represent the ratio of polykaryon-forming cells to the total number of cells counted. The values were significantly lower in groups treated with Stalk antisera specific to group 1 H1N1, rH5N1, rH6N2, and rH9N2 when compared with the same groups treated with sera from PBS control mice. The fusion indexes did not differ in the group 2 H3N2 virus group when cells were treated with sera from PBS control mice. Overall, these results indicate that immune sera from Stalk-Fc–immunized mice block viral fusion with endosomal membranes induced by low pH, a crucial step in the viral replication cycle.

### Stalk-specific Ab Fc-mediated effector mechanisms.

Beyond direct virus neutralization, influenza virus HA Stalk–specific Abs have been shown to possess potent Fc-mediated effector functions, which are important in ameliorating disease severity by eliminating virus-infected cells (51–54). Several HA Stalk–based vaccines have been shown to stimulate Ab-dependent cellular cytotoxicity (ADCC) and Ab-dependent cellular phagocytosis (ADCP) in both animal models and humans (55, 56). Meanwhile, some HA Stalk–specific Abs promote the lysis of virus-infected cells via complement-dependent cytotoxicity (CDC) (57) that correlates with protection against influenza illness after seasonal influenza vaccination in pregnant women (54). Hence, we evaluated the ADCC, ADCP, and CDC activities of HA Stalk–specific Abs against influenza virus–infected cells. We first measured ADCC activity using a high-throughput mouse FcγRIV activated reporter assay. The H1N1 influenza virus–infected MDCK cells were subjected to treatment with antisera from different immunization groups, followed by incubation with Jurkat reporter cells expressing mouse FcγRIV. The reporter gene can only be activated by the sera from Stalk-Fc/wt vaccinated WT mice, but not those from other groups (Figure 8A, left). The elevated reporter signal by Stalk-Fc/wt sera was also remarkable in MDCK cells infected with other group 1 viruses, including rH5N1, rH6N2, and rH9N2, but was not detectable in H3N2 (group 2) virus-infected cells (Figure 8A, right). These results suggest that the cross-reactive Abs in immune sera predominantly target HA from group 1 but not group 2 influenza A virus. We further examined the functional CDC activity of Stalk Ab by incubating virus-infected MDCK cells with the diluted mouse sera and guinea pig complement. The subsequent cell death was measured using a cell viability detection kit and the percentage of cytotoxicity caused by the Stalk-specific Ab (Figure 8B). The combination of Stalk-Fc/wt–stimulated mouse sera, but not the PBS-immunized sera, with exogenous complement resulted in significantly higher cytotoxicity in target cells infected with H1N1, rH5N1, rH6N2, and rH9N2 strains. However, the Ab-specific cell lysis was minimal in H3N2 strain–infected cells.

Phagocytic macrophages (58) and neutrophils (59) have been shown to recognize the Fc portion of Abs via their FcRns, then engulf and destroy the Ab-coated target cells or virus particles, a process known as ADCP. To assess the ADCP function of Stalk Ab in immune sera, we employed a microsphere-based approach as previously described (60). The yellow/green fluorescent beads coated with various influenza HA proteins were used as a surrogate of virus-infected target cells. Beads were incubated with mouse sera and loaded on J774 or BMDM cells. The internalization of beads was visualized using a fluorescence microscope (Figure 8, C and D, and Supplemental Figure 12) or monitored using flow cytometry (Figure 8, E and F, and Supplemental Figure 13). In the presence of serum from Stalk-Fc/wt–immunized mice, a significantly high number of beads coated with HA1, HA5, or HA9 proteins were internalized by both cells (Figure 8C and Supplemental Figure 12, A and B, and Supplemental Figure 14), indicating the enhanced engulfment by Stalk Ab is broad for group 1 HA proteins. As expected, this enhancement was not observed in the PBS serum–treated samples. Furthermore, immune sera containing Stalk Abs showed negligible ADCP activity against HA4-coated beads, which can be attributed to the high diversity of group 2 HA4 protein from the group 1 HA protein (Figure 8D and Supplemental Figure 12C). Therefore, i.n. administration of HA Stalk-Fc/wt resulted in a significant increase in HA Stalk–specific ADCP activity within the immune sera.

Taken together, our results demonstrate that serum Abs elicited by FcRn-mediated nasal delivery of Stalk-Fc/wt protein directly protect through the Fc-mediated effector function, which effectively eliminates the virus-infected cells via multiple mechanisms, including ADCC, ADCP, or CDC. This may partially explain the protection against the heterologous or heterosubtypic infection from group 1 influenza virus strains in mice (Figures 4 and 5).

## Discussion

The goal of universal vaccines is to protect against all influenza A and B viruses, including those causing seasonal outbreaks and pandemics. The HA Stalk is a promising target due to its conservation across strains; however, the highly immunogenic HA head can divert the immune response away from the Stalk, resulting in fewer Stalk-specific Abs (12, 23, 24, 61). Strategies like chimeric HA, hyperglycosylated HA, headless HA, and mosaic HA are being explored to enhance Stalk-specific Ab responses (2, 62). This study explores an approach utilizing FcRn for the nasal delivery of the HA Stalk antigen.

Local immune responses are crucial for defending against initial viral infections in the respiratory tract (63). Since the influenza virus infects epithelial cells via droplets and aerosols, an effective universal influenza vaccine should induce durable local immunity in the airway to block initial viral entry and replication. This can only be achieved by delivering vaccine antigens through the airway, stimulating the production of sIgA in the upper respiratory tract or IgG in BAL fluid and promoting the generation of TRMs in the nasal tissue and lungs (7–9). We found that FcRn-mediated nasal delivery of HA Stalk significantly enhanced local immune responses. First, FcRn transports Stalk-Fc/wt across the respiratory barrier. FcRn binds IgG preferably at a pH below 6.5 and releases it at neutral or higher pH. The pH of airway surface liquid is typically slightly acidic, which favors IgG binding by FcRn. Second, mice immunized with HA Stalk-Fc/wt produced high levels of IgA and IgG Abs in nasal secretions, BAL, and blood. sIgA serves as a critical defense by preventing viral attachment to epithelial cells. Also, either sIgA or IgG Abs can mediate intracellular neutralization of influenza virus in epithelial cells (64). Several studies showed that vaccination with influenza vaccines boosts existing Ab responses to HA Stalk (65–67). We suggest that FcRn-mediated airway delivery of Stalk-Fc can likely stimulate HA Stalk–specific memory B cells. Third, Stalk-Fc/wt i.n. immunization resulted in significant numbers of monofunctional and polyfunctional CD4^+^ TRM T cells in the lungs. These results align with recent findings that CD4^+^ TRM T cells in the nasal tissues provide cross-protective immunity against influenza viruses (9). Studies have shown that TRM T cells can only be induced via i.n., but not by i.m., immunization (7, 22, 38). Further research is needed to assess the longevity and specific role of CD4^+^ and CD8^+^ TRM T cells against influenza infections.

FcRn-mediated delivery of HA Stalk induces a broad immune response against various lethal influenza virus subtypes. Several lines of evidence support this conclusion. First, the correctly folded HA Stalk-Fc is recognized by several conformation-dependent, Stalk-specific mAbs. Serum from Stalk-Fc/wt–immunized mice competitively binds to Stalk with broad neutralizing mAbs FI6v3, 6F12, and KB2. Second, serum Abs from these mice can bind to various HA proteins from HA1, HA2, HA5, HA9, and HA13 influenza viruses. Third, most Stalk-Fc/wt–immunized mice resist infections from H1N1, H5N1, H6N2, and H9N2 viruses, showing reduced virus replication in the nasal tissue and lungs. Although the immune sera showed some cross-reactivity with group 2 HA antigens, significant ADC, ADCP, or CDC activities against H3N2 or H4N6 viruses were lacking, likely due to structural differences of groups 1 and 2 HA Stalks (43). Indeed, most of the mice immunized with Stalk-Fc/wt failed to survive H3N2 infection. In general, a universal influenza vaccine is expected to protect against both groups 1 and 2 viruses (46, 68). This study focuses on Stalk-Fc using group 1 influenza A HA, with plans to include the HA stalks from group 2 and influenza B viruses. Furthermore, incorporating matrix protein 2 with HA Stalk can further enhance protection against multiple influenza strains (69). Combining these strategies into a multivalent mucosal vaccine using FcRn delivery warrants further investigation.

A universal flu vaccine aims to provide long-lasting protection through multifaceted memory immune responses (46, 70), including virus-specific memory T and B cells, as well as long-lived plasma cells. This study found that i.n. immunization with Stalk-Fc/wt resulted in sustained levels of Stalk-specific IgA and IgG Abs in nasal washes and BAL fluid even 10–16 months after the boost. Mice immunized with HA Stalk-Fc/wt had a significant number of long-lived IgA- or IgG-secreting cells in their bone marrow, which contributed to these sustained Ab levels (47). Most of the mice also resisted lethal H5N1 infection for at least 6 months after the boost. Thus, the durable mucosal and systemic response induced by FcRn delivery may help advance a universal nasal flu vaccine in humans, potentially reducing viral infections and transmission in the community.

Several mechanisms may explain the protection against lethal influenza virus infection in mice immunized via FcRn-mediated delivery of Stalk-Fc antigen. First, the immunized mice develop specific neutralizing Abs against H5N1. The Stalk-specific neutralizing Abs prevent the virus from fusion with host cellular membrane by locking the HA in a prefusion state (12). Indeed, this Stalk-Fc–induced immune response markedly reduced viral and cellular membrane fusion induced by group 1 influenza viruses. This result differs from a finding that anti-Stalk Abs induced by inactivated vaccine promote virus fusion (71). Second, HA Stalk–specific Abs can engage other immune mechanisms, such as ADCC, CDC, or ADCP activities (Supplemental Figure 16) against influenza virus infection, which are principally mediated by Fc receptor (FcR) activation of immune effector cells (52, 54, 58, 72). Stalk-specific Abs are reportedly more potent in inducing CDC due to their binding proximity to the infected cell membranes, facilitating cell lysis (33, 73). Third, the Fc portion of Stalk-Fc enhances nasal delivery and half-life of the antigen (11), promoting uptake by DCs and macrophages (Supplemental Figure 15, A–C) for better antigen presentation to T cells. Indeed, FcRn is also involved in Ab-mediated phagocytosis (74) and enhancing antigen or cross-antigen presentations in macrophages and DCs (75, 76). This process may explain the higher percentages of lung TRM T cells induced by Stalk-Fc/wt. Additionally, anti-HA Stalk Abs may inhibit influenza NA activity, aiding viral neutralization (77–79). Further investigations should assess whether these Abs limit NA access to sialic acids on the virion surface.

A safe and more effective universal influenza nasal vaccine is needed to protect high-risk populations, including the young and elderly (46). The live attenuated vaccine FluMist is currently approved for individuals from ages 2 to 49, but the HA Stalk-Fc may serve as a suitable candidate for all ages. While HA Stalk–directed vaccination strategies induce cross-reactive immunity, their weak immunogenicity requires multiple or high doses, particularly in vulnerable groups (80, 81). In this study, mice i.n. immunized with Stalk alone exhibited poor immune responses and minimal protection. However, fusing an IgG Fc to HA Stalk for FcRn targeting significantly enhances its immunogenicity when administered nasally, resulting in enhanced local and systemic immune responses and protection. Additionally, people with prior influenza infections or vaccinations often have baseline Stalk-directed Abs or preexisting memory B cells (65, 67, 82, 83), which may further enhance the Stalk-Fc–induced immune responses. Indeed, a single i.n. boost with the Stalk-Fc protein in mice with preexisting immunity significantly enhances immune responses and reduces viral replications in the respiratory tract. Additionally, combining CpG with c-di-GMP or other potent and safe mucosal adjuvants could further boost the Stalk immunogenicity in humans. Overall, FcRn-mediated delivery shows promise for improving the effectiveness of influenza virus HA Stalk antigens as a universal influenza nasal vaccine.

## Methods

All procedures are described in detail in Supplemental Methods.

### Sex as a biological variable.

Both male and female C57BL/6 mice were examined for immune responses in this study, and similar findings are reported for both sexes. Since female mice are more susceptible to the influenza A virus infection, they were used to examine vaccine efficacy in the virus challenge study.

### Statistics.

All data were analyzed using GraphPad Prism 10.0 software. For normally distributed data, an unpaired 2-tailed *t* test was employed to compare means between 2 groups, while 1-way ANOVA was used to assess differences among 3 or more groups. Additionally, a post hoc test was applied following 1-way ANOVA. Dunnett’s multiple-comparison test was used to compare means from different treatment groups against a single control group. If the data distribution was not normal, the Mann-Whitney test (2-tailed, for 2 groups) or the Kruskal-Wallis test (for more than 2 groups) was employed. To compare Kaplan-Meier survival curves, the log-rank (Mantel-Cox) test was used. Statistical methods for each experiment are indicated in the figure legends. *P* values < 0.05 were considered significant. Significance levels were further classified as *P* < 0.05, *P* < 0.01, *P* < 0.001, and *P* < 0.0001.

### Study approval.

All biosafety and animal experimental procedures used in this study were approved by the Institutional Biosafety Committee and Institutional Animal Care and Use Committee of the University of Maryland.

### Data availability.

Values for all data points in graphs are included in the Supporting Data Values file.

## Manuscript information

### Author contributions.

WL and XZ designed experiments and analyzed data. WL, WT, and XZ wrote the paper. WL, AMR, ZM, BD, BMD, TW, QK, and QW conducted experiments. WT, QW, FK, HW, and ZY provided critical materials, interpreted data, and made editorial suggestions.

### AI disclosure.

The generative AI tool ChatGPT (free version) was used to create the airway epithelial layer image in the Graphical Abstract in June 2026.

### Conflict of interest.

XZ is listed as an inventor on US patent 9,238,683 B2, entitled “Efficient Mucosal Vaccination Mediated by the Neonatal Fc receptor.” XZ and WL are listed as inventors on US patent 12,297,233, entitled “FcRn-targeted mucosal vaccination against influenza infections.”

### Funding.

This study was supported by the following:

- NIH grants AI146063 and AI190355 (to XZ).
- USDA National Institute of Food and Agriculture A1181 Biosecurity grant 2025-39601-44631 (to XZ and WT).
- Maryland Agricultural Experiment Station grants from the University of Maryland (to XZ).
- The Basil and Anne Hatziolos Scholarship Fund for Veterinary Medical Research (to ZM and B Dhakal).
- The USDA ARS intramural research program (WT and AMR).

This work is the result of NIH funding, in whole or in part, and is subject to the NIH Public Access Policy. Through acceptance of this federal funding, the NIH has been given a right to make the work publicly available in PubMed Central.

## Supplementary Material

Supplemental data

Unedited blot and gel images

Supporting data values

## Figures and Tables

**Figure 1:**
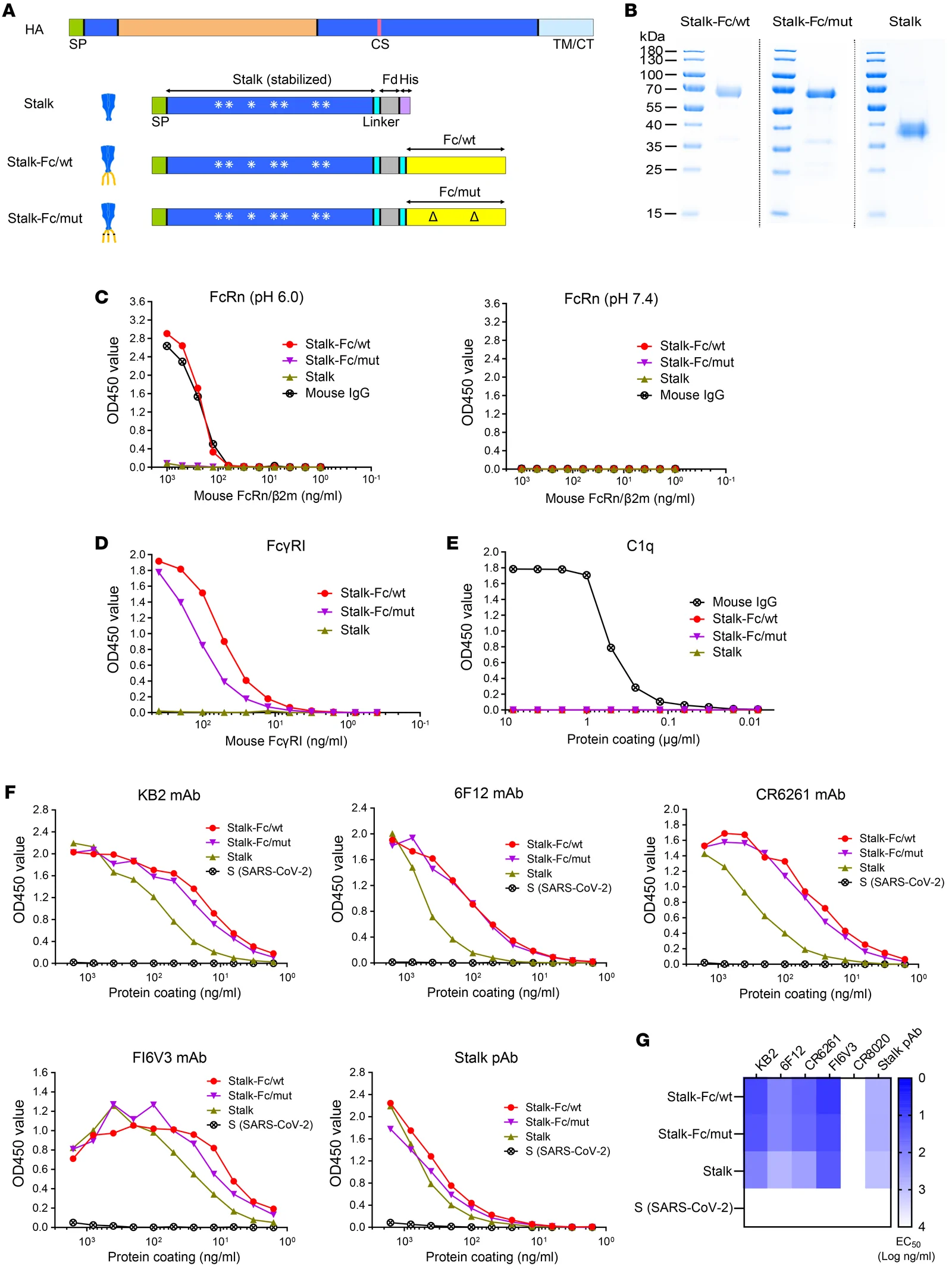
Expression and characterization of prefusion HA Stalk and Stalk-Fc proteins. (**A**) Schematic of influenza A virus HA protein, including signal peptide (SP), cleavage site (CS), transmembrane domain (TM), and cytoplasmic tail (CT). The graphic shows the fusion of the HA Stalk with the T4 fibritin foldon domain (Fd) and His tag to produce a soluble, prefusion-stabilized HA Stalk protein. Specific mutations, indicated by asterisks, were introduced in the HA Stalk to stabilize the prefusion form. Additionally, a Stalk-Fc/wt protein was generated by fusing Stalk with foldon and mouse Fcγ2a Fc. Mutations were created in the hinge region by replacing 3 cysteines with serine residues and in the Fc region by modifying Glu318, Lys320, and Lys322 to alanine residues to eliminate the C1q binding site. The Stalk-Fc/mut construct was also generated by incorporating HQ/AD (310/311) and HN/AQ (433/434) mutations (indicated by triangles) to disrupt FcRn binding. To ensure proper domain folding, G/S linkers are included. (**B**) The Stalk-Fc and Stalk proteins were purified from stable CHO cell lines. Both soluble Stalk-Fc and Stalk proteins were isolated using affinity columns and subsequently analyzed by SDS-PAGE under reducing conditions, followed by Coomassie blue staining. (**C–E**) The binding of Stalk-Fc to mouse FcRn/β2m, FcγRI, and C1q was measured by ELISA to assess the specific interactions. Stalk protein served as a negative control and mouse IgG as a positive control. (**F**) The interactions of Stalk-Fc or Stalk alone with HA Stalk–specific and conformation-dependent mAbs KB2, 6F12, CR6261, and FI6V3 were examined by ELISA. Stalk-specific polyclonal Ab (pAb) was used as a positive control, and a trimeric SARS-CoV-2 Spike (S) protein as a negative control. (**G**) A heatmap displaying the EC_50_ values derived from the results in **F** and Supplemental Figure 3.

**Figure 2:**
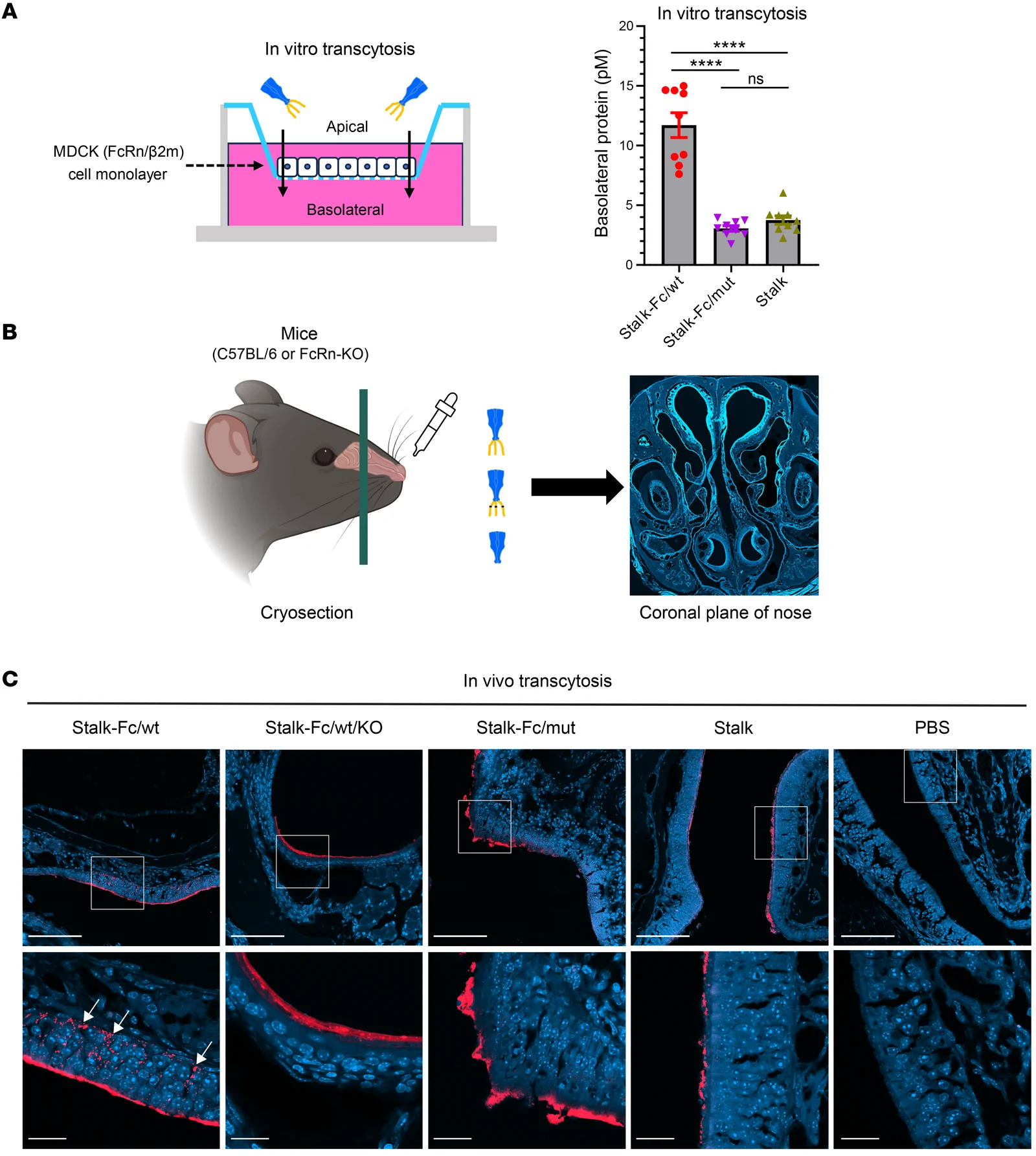
FcRn-mediated transport of the HA Stalk-Fc/wt protein across polarized epithelial cells. (**A**) In vitro transcytosis of the Stalk-Fc/wt across MDCK cell monolayers expressing FcRn and β2m. MDCK^FcRn/β2m^ cells were grown on the apical side of Transwell inserts to become fully polarized. Stalk-Fc (50 μg/mL) or Stalk (27.5 μg/mL) proteins were added to the apical side and incubated at 37°C for 90 min. An ELISA was conducted to measure the amount of protein transported to the basolateral side. The data presented are the mean ± SEM from 9 individual wells per protein across 2 independent assays. Statistical analysis was conducted using Welch’s 1-way ANOVA (with Dunnett’s T3 multiple-comparison test). ****P < 0.0001. (**B**) Schematic showing the process of making a nose cryosection for the imaging analysis of in vivo transcytosis. (**C**) In vivo transcytosis of Stalk-Fc/wt proteins across the epithelial cells lining the nasal cavities of mice. Stalk-Fc or Stalk proteins were conjugated with the fluorescent dye AF647. A total of 10 μg of Stalk-Fc/wt, Stalk-Fc/mut, or 5.5 μg of Stalk proteins was i.n. inoculated into C57BL/6 or FcRn KO mice (*n* = 3) in a volume of 20 μL. 7 h later, nasal tissue samples were fixed in 10% formalin, decalcified, embedded in OCT compound, and sectioned using a cryostat. Protein distributions in the nasal epithelium (arrows) were visualized using confocal microscopy after DAPI staining. Representative images display a ventral view of the mouse noses. Scale bars: 60 μm (top), 10 μm (bottom).

**Figure 3:**
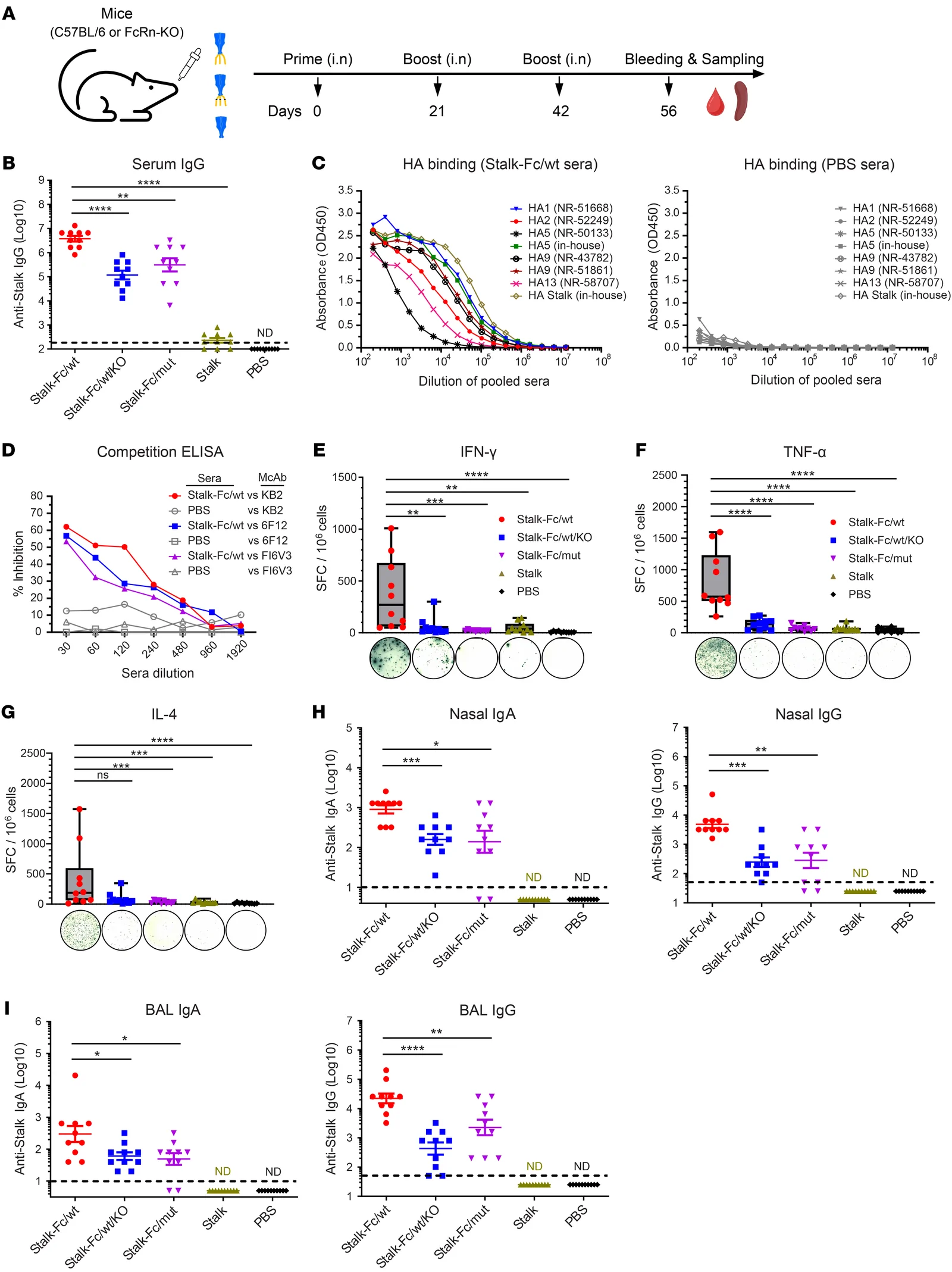
FcRn-mediated i.n. immunization significantly enhances HA Stalk–specific Ab immune responses. (**A**) 10 μg of Stalk-Fc, 5.5 μg Stalk (equivalent molar), or PBS in combination with 10 μg of CpG or 5 μg of c-di-GMP in 20 μL was i.n. administered into 7-week-old C57BL/6 or FcRn KO mice (*n* = 10). Mice were boosted twice at a 3-week interval. Sampling was done at the indicated time points. (**B**) The levels of Stalk-specific IgG were measured by ELISA. Symbols represent individual mice, and the horizontal lines indicate the mean ± SEM. (**C**) The broad bindings of the sera from the Stalk-Fc/wt– or PBS-immunized mice were examined by interacting with the HA1, HA2, HA5, HA9, and HA13 proteins from different influenza subtypes in ELISA. (**D**) The competitive Stalk binding of the sera from the mice immunized with Stalk-Fc/wt or PBS with the Stalk-specific mAbs KB2, 6F12, and FI6V3 was measured by ELISA. (**E**–**G**) Cytokine-secreting cells in the spleen. The spot-forming cells (SFCs) secreting IFN-γ (**E**), TNF-α (**F**), and IL-4 (**G**) per 1 × 10^6^ splenocytes from the immunized mice were quantified by ELISpot. The representative images (5 × 10^5^ cells /well) are shown below the plot. Data are presented as box-and-whisker plots with minimum, maximum, median, and interquartile ranges. (**H a**nd **I**) The levels of Stalk-specific IgA and IgG Abs in nasal wash (**H**) or BAL (**I**) were measured by ELISA; data are presented as mean ± SEM. Statistical analyses were conducted using ordinary 1-way ANOVA with Dunnett’s multiple-comparison tests for **G** and **I**; Welch’s 1-way ANOVA with Dunnett’s T3 multiple comparisons for **B**, **E**, **F**, and **H** (left); or Kruskal-Wallis test with Dunn’s multiple comparisons for **H** (right). *P < 0.05, **P < 0.01, ***P < 0.001, ****P < 0.000. ND: not detected, which was excluded from statistical analysis.

**Figure 4:**
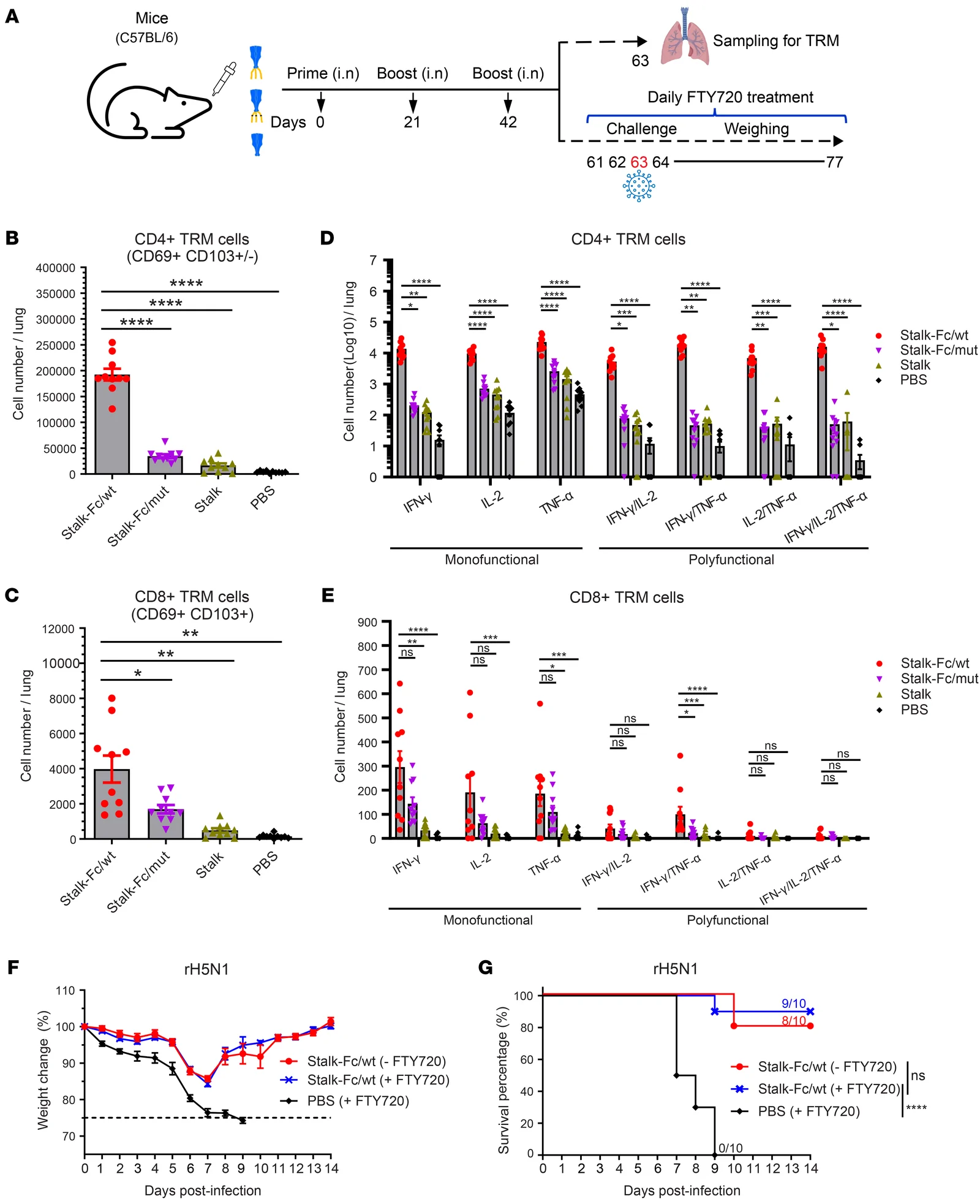
TRM CD4^+^ and CD8^+^ T cell responses in the lungs. (**A**) Schematic depicting procedures for immunization and collection of TRM cells (top) and influenza virus challenge in mice with or without FTY720 treatment (bottom). For the FTY720 treatment, Stalk-Fc/wt– or PBS-immunized mice received daily i.p. injection with 20 �g FTY720 in 100 �L 0.9% saline 2 days before and throughout the challenge period. The immunized mice administered with 0.9% saline served as controls. (**B** and **C**) TRM cells in the lungs (*n* = 10/group). The number of CD4^+^ TRM (CD69^+^CD103^+/–^) (**B**) or CD8^+^ TRM (CD69^+^CD103^+^) (**C**) T cells from the lungs of each mouse were quantified and plotted as mean ± SEM. The data were combined from 2 independent experiments. (**D** and **E**) The TRM cells were functionally evaluated by measuring the production of IFN-γ, TNF-α, and IL-2 cytokines after HA peptide stimulation. The number of CD4^+^ (**D**) or CD8^+^ (**E**) TRM-secreting single or multiple cytokines in the lung is presented as mean ± SEM. (**F** and **G**) The immunized mice treated with or without FTY720 were i.n. infected with rH5N1 virus (5 × 10^2^ TCID_50_), and body weight changes were monitored daily for 14 days (**F**). Protection is shown as a Kaplan-Meier survival curve (**G**). Statistical analyses were conducted using an ordinary 1-way ANOVA with Dunnett’s multiple-comparison test for TNF-α (**D**); Welch’s 1-way ANOVA with Dunnett’s T3 multiple-comparison test for CD4^+^ TRM (**B**), CD8^+^ TRM (**C**), and IL-2 cytokine (**D**); or Kruskal-Wallis test followed by Dunn’s multiple comparisons (**D** and **E**). *P < 0.05, **P < 0.01, ***P < 0.001, ****P < 0.0001.

**Figure 5:**
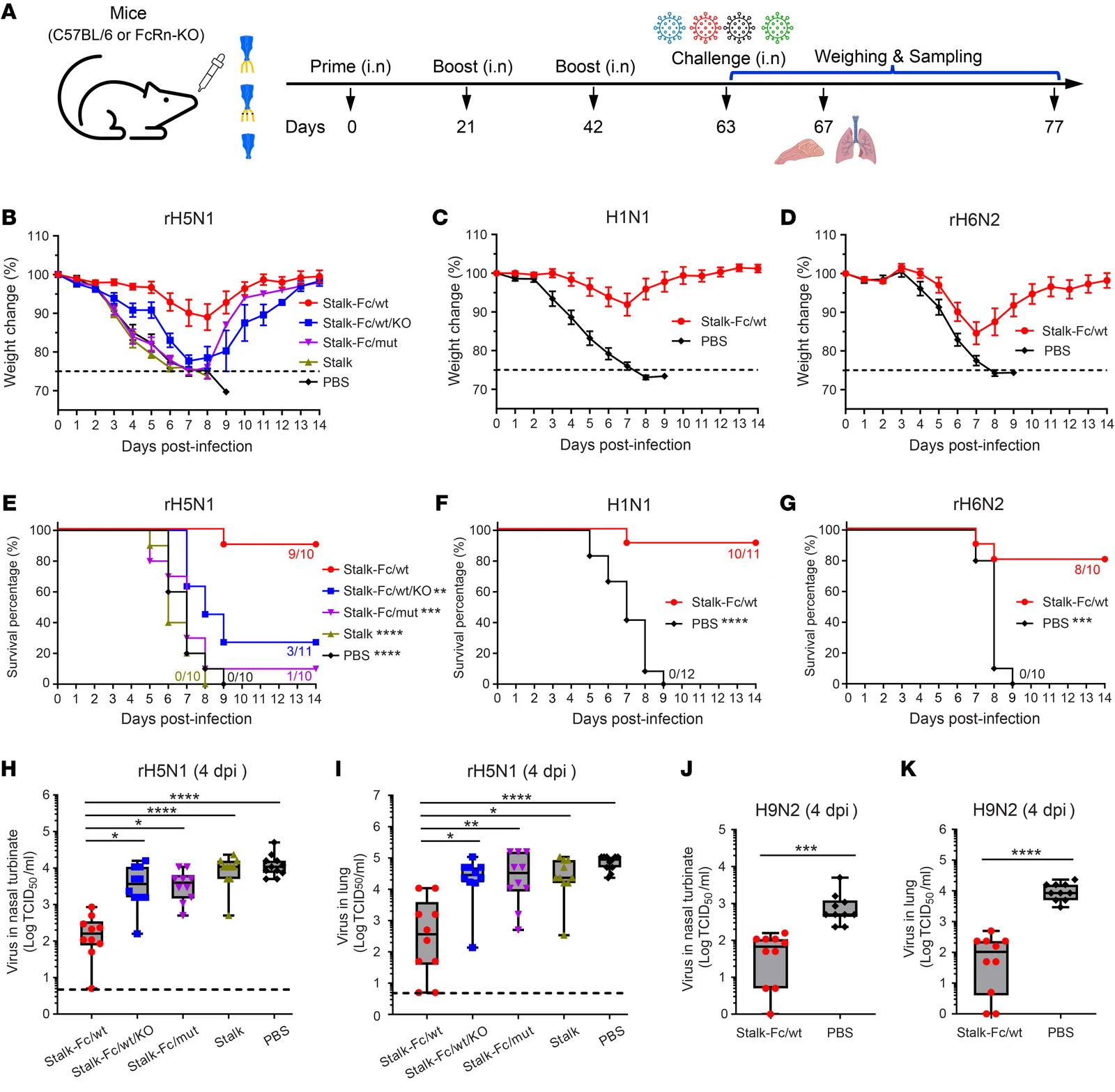
The i.n. immunization by HA Stalk-Fc/wt induces broad protection against the challenge of different subtypes of influenza A virus. (**A**) Schematic of the immunization and challenge procedures. The 7-week-old C57BL/6 or FcRn KO mice were i.n. immunized with 10 μg of Stalk-Fc or 5.5 mg of Stalk protein. 10 μg CpG or 5 μg c-di-GMP was used as an adjuvant. Mice were boosted twice at a 3-week interval. 3 weeks after the final boost, the mice were i.n. challenged with 50 mL of a lethal dosage of mouse-adapted influenza virus rH5N1, H1N1, or rH6N2 or a field strain of H9N2. (**B**–**D**) The body weights were measured daily for 14 days in each experiment, and the data represent the mean ± SEM (*n* = 10–12 per group). Mice were euthanized when 25% body weight loss or a humane endpoint was reached. (**E**–**G**) The survival rates are plotted by Kaplan-Meier curves (*n* = 10–12 per group). (**H**–**K**) rH5N1 or H9N2 virus replication in the nasal turbinate (**H** and **J**) and lungs (**I** and **K**) at 4 days post infection was also evaluated. The virus titers in the samples (*n* = 10–11 per group) were determined by cytopathic effect (CPE) in MDCK cells and shown as TCID_50_. Data are presented as box-and-whisker plots with minimum, maximum, median, and interquartile ranges. Statistical analyses were conducted using a log-rank (Mantel-Cox) test (**E**–**G**), Kruskal-Wallis test followed by Dunn’s multiple comparisons (**H** and **I**), unpaired 2-tailed *t* test (**J**), or an unpaired 2-tailed *t* test with Welch’s correction (**K**). *P < 0.05, **P < 0.01, ***P < 0.001, ****P < 0.0001.

**Figure 6:**
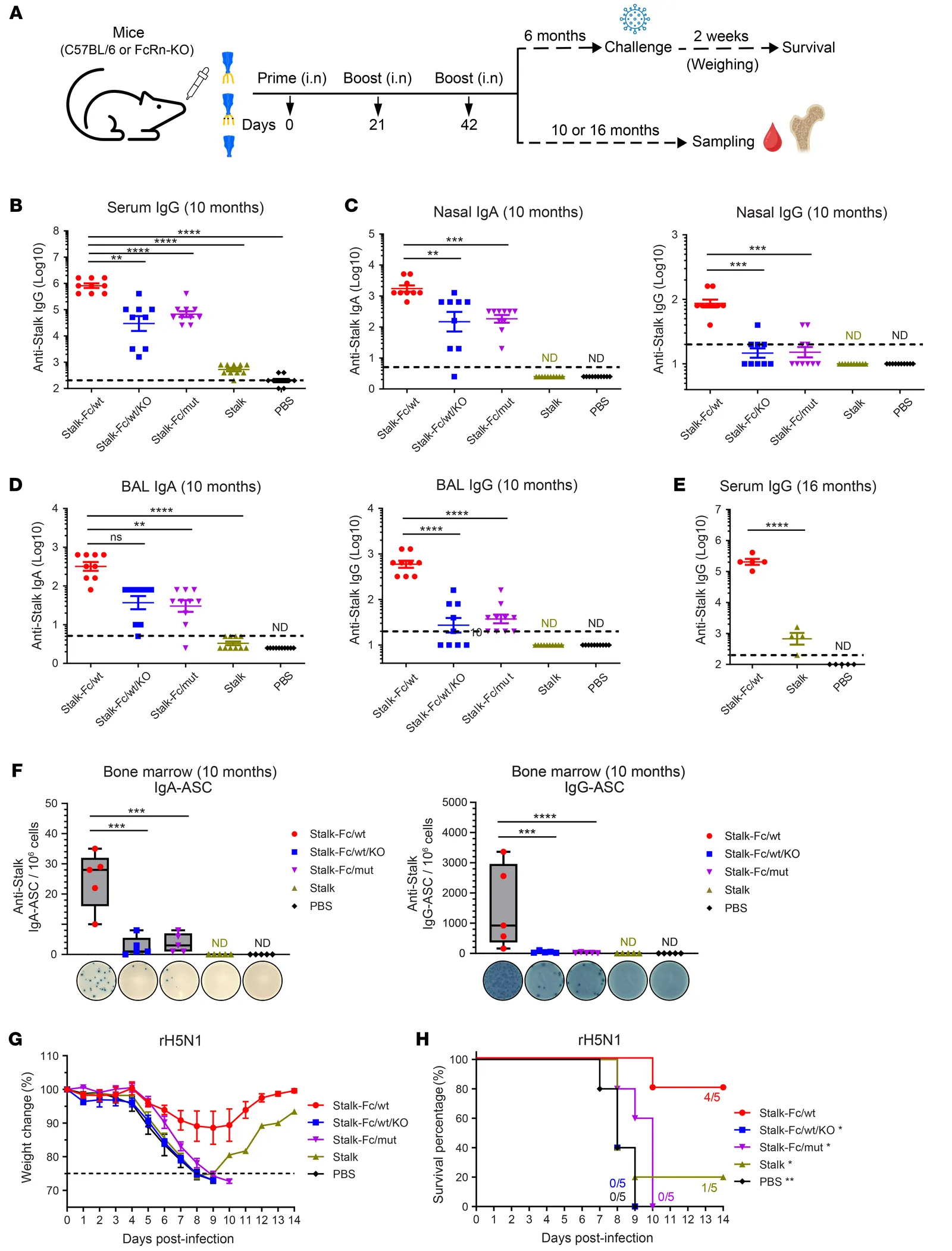
FcRn-mediated i.n. immunization with HA Stalk-Fc enhances memory immune responses. (**A**) Schematic of the experimental design to evaluate the memory immune responses. Mice were i.n. immunized at the indicated time and euthanized for sampling sera, nasal washes, BAL, and bone marrow. Some mice were subjected to rH5N1 virus challenge. (**B**–**E**) The duration of Stalk-specific Ab responses was assessed 10 or 16 months after the final boost. Stalk-specific IgG Ab in mouse sera (**B** and **E**), nasal washes (**C**, right), and BAL (**D**, right) or IgA in nasal washes (**C**, left) and BAL (**D**, left) were quantified by ELISA and exhibited as mean ± SEM (*n* = 9–10 for 10 month samples, and *n* = 4–5 for 16 month samples). (**F**) Long-lived Stalk-specific Ab-secreting cells (ASCs) in BM were isolated after 10 months of final boost. The 2.5 × 10^5^ cells/well from the Stalk-Fc/wt group and 1 × 10^6^ cells/well from other groups were placed on Stalk-coated plates and quantified by ELISpot analysis of IgA-secreting (left) or IgG-secreting (right) plasma cells. Boxes illustrate median values as well as 25th and 75th percentile values (*n* = 5 per group). Representative spot images are shown. (**G**) Body weight changes following rH5N1 virus challenge. 6 months after the final boost, groups of 5 mice were i.n. challenged with rH5N1 virus (5 × 10^2^ TCID_50_) and weighed daily for 14 days. (**H**) Protection is shown as a Kaplan-Meier survival curve. Statistical analyses were conducted using Welch’s 1-way ANOVA with Dunnett’s T3 multiple comparisons (**B**), Kruskal-Wallis test followed by Dunn’s multiple comparisons (**C** and **D** [left]), ordinary 1-way ANOVA with Dunnett’s multiple comparisons (**D** [right] and **F**), unpaired 2-tailed *t* test (**E**), or log-rank (Mantel-Cox) test (**H**). *P < 0.05, **P < 0.01, ***P < 0.001, ****P < 0.0001. ND: not detected, which was excluded from statistical analysis.

**Figure 7:**
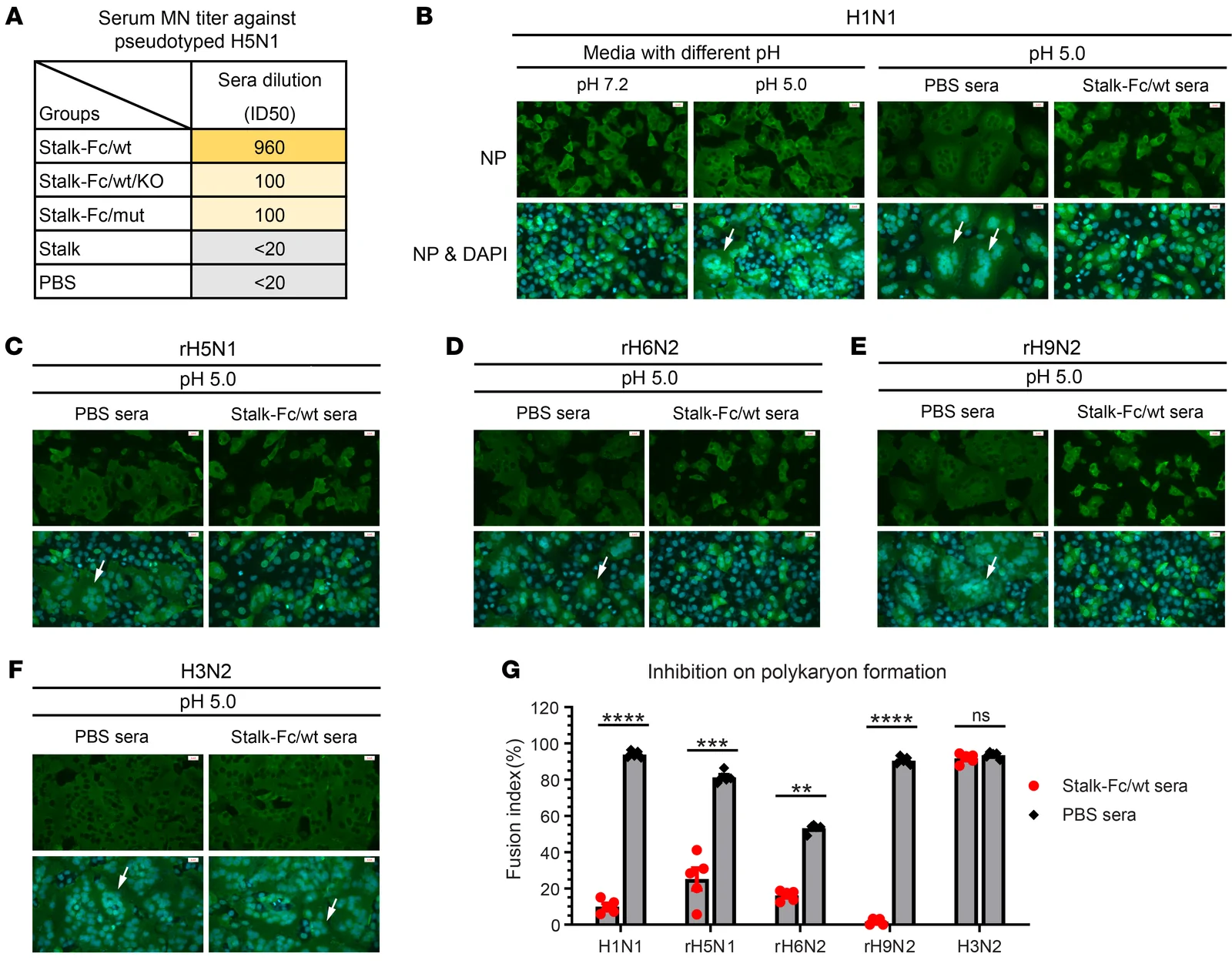
Effects of immune serum–mediated neutralizing activity and the inhibition of the membrane fusion. (**A**) The microneutralization titer of the pooled mouse sera against the pseudotyped H5N1 reporter virus is expressed as the average ID_50_ from 2 independent tests. (**B**–**F**) Sera samples were evaluated for their ability to inhibit the pH-dependent cell–cell fusion in influenza A virus–infected cells. Vero cells were infected with different influenza viruses for 12 h: H1N1 (**B**, MOI = 0.2), rH5N1 (**C**, MOI = 0.04), rH6N2 (**D**, MOI = 0.4), rH9N2 (**E**, MOI = 0.4), or H3N2 (**F**, MOI = 0.4). Following infection, the cells were treated with TPCK-trypsin (5 �g/mL) for 7 min. Pooled sera from the Stalk-Fc/wt–immunized mice or PBS-control mice were diluted and added to cells for 1.5 h incubation. Cell–cell fusion was triggered by exposing the cells to pH 5.0 PBS for 10 min, whereas pH 7.2 PBS served as a control. After an additional 3 h of culture at 37°C in fresh media, the cells were stained with a nucleoprotein (NP)-specific Ab and DAPI. Membrane fusion and polykaryon formation were observed using fluorescence microscopy. Scale bars: 127 μm. (**G**) The fusion index of the virus-infected cells treated with sera from Stalk-Fc/wt–immunized or PBS control mice was calculated from 5 random fields under microscope. Fusion index (%) = total number of nuclei in the infected cells forming polykaryon/total number of nuclei in all infected cells.

**Figure 8:**
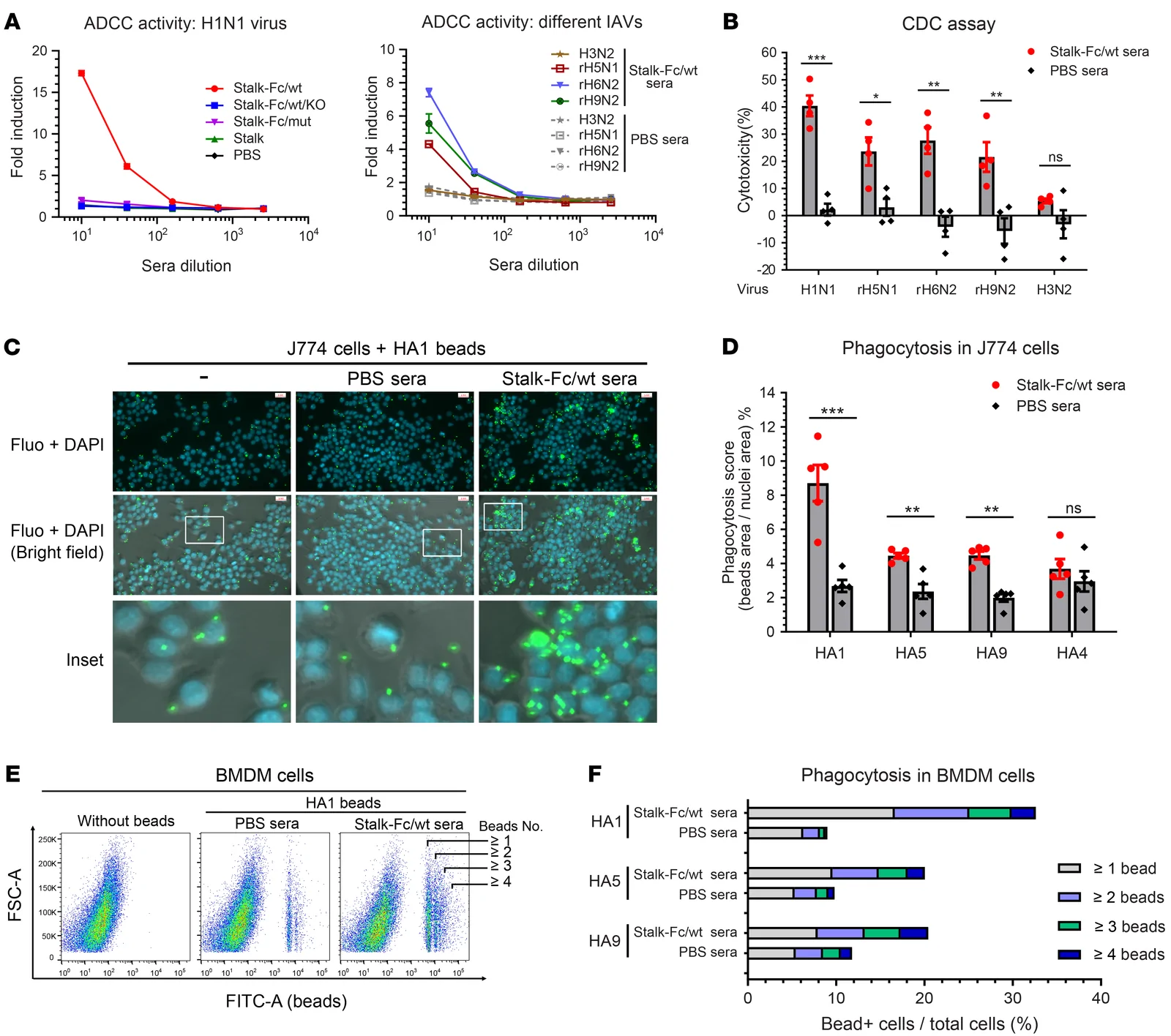
Evaluation of ADCC, CDC, and ADCP activities of immune sera. (**A**) ADCC assay. MDCK cells that were infected with the indicated influenza viruses were incubated with serially diluted immune sera. Jurkat reporter cells were added for measuring luciferase activity. Each symbol represents the mean value from duplicate wells. Data are shown as the ADCC fold change between wells treated with or without serum. IAV, influenza A virus. (**B**) CDC assay. Influenza virus–infected MDCK cells were incubated with the pooled immune or control sera in the presence of 10% complement to determine the lysis. Each symbol represents the values of duplicate samples from 2 independent experiments. (**C**) ADCP assay. HA1-coated fluorescence beads that were incubated with immune sera were added to J774 cells for phagocytosis. The cell nucleus was stained with DAPI. Fluo, fluorescence. Scale bars = 127 μm. (**D**) Quantitative analysis of phagocytosis in J774 was performed using beads coated with the indicated HA proteins. For each sample, images were captured from 5 random fields. The areas of beads and nuclei in each field were calculated with ImageJ (NIH), and the resulting ratio represents the phagocytosis score. (**E** and **F**) Phagocytosis analysis in BMDMs. Cells containing HA-coated beads were identified by flow cytometry and are shown in dot plots from a representative result (**E**). A stacked column graph (**F**) illustrates the percentage of cells internalizing different numbers of HA-coated beads (≥1, 2, 3, or 4 beads) within the total cell populations. The fractions within each column indicate the mean values from 2 experiments. Statistical analyses were conducted using an unpaired 2-tailed *t* test for the H1N1, rH5N1, rH6N2, and rH9N2 viruses or an unpaired 2-tailed *t* test with Welch’s correction for H3N2 virus in **B**, or an unpaired 2-tailed *t* test for HA1, HA5, and HA4 or a Mann-Whitney test for HA9 in **D**. *P < 0.05, **P < 0.01, ***P < 0.001.

## Version 1 11/16/2026

Electronic publication

## References

[1] Bouvier NM, Palese P (2008). The biology of influenza viruses. Vaccine.

[2] Bullard BL, Weaver EA (2021). Strategies targeting hemagglutinin as a universal influenza vaccine. Vaccines (Basel).

[3] Le Sage V (2023). Block the spread: barriers to transmission of influenza viruses. Annu Rev Virol.

[4] Krammer F (2019). The human antibody response to influenza A virus infection and vaccination. Nat Rev Immunol.

[5] Brokstad KA (2002). Parenteral vaccination against influenza does not induce a local antigen-specific immune response in the nasal mucosa. J Infect Dis.

[6] Bean R (2024). Mucosal correlates of protection after influenza viral challenge of vaccinated and unvaccinated healthy volunteers. mBio.

[7] Zens KD (2016). Vaccine-generated lung tissue-resident memory T cells provide heterosubtypic protection to influenza infection. JCI Insight.

[8] Pizzolla A (2017). Resident memory CD8+ T cells in the upper respiratory tract prevent pulmonary influenza virus infection. Sci Immunol.

[9] Mathew NR (2026). Nasal CD4+ tissue-resident memory T cells provide cross-protective immunity to influenza. J Exp Med.

[10] Spiekermann GM (2002). Receptor-mediated immunoglobulin G transport across mucosal barriers in adult life: functional expression of FcRn in the mammalian lung. J Exp Med.

[11] Roopenian DC, Akilesh S (2007). FcRn: the neonatal Fc receptor comes of age. Nat Rev Immunol.

[12] Krammer F, Palese P (2013). Influenza virus hemagglutinin stalk-based antibodies and vaccines. Curr Opin Virol.

[13] Kirkpatrick E (2018). The influenza virus hemagglutinin head evolves faster than the stalk domain. Sci Rep.

[14] Christensen SR (2022). The 2009 pandemic H1N1 hemagglutinin stalk remained antigenically stable after circulating in humans for a decade. J Virol.

[15] Darricarrère N (2021). Broad neutralization of H1 and H3 viruses by adjuvanted influenza HA stem vaccines in nonhuman primates. Sci Transl Med.

[16] Moin SM (2022). Co-immunization with hemagglutinin stem immunogens elicits cross-group neutralizing antibodies and broad protection against influenza A viruses. Immunity.

[17] Guthmiller JJ (2022). Broadly neutralizing antibodies target a haemagglutinin anchor epitope. Nature.

[18] Andrews SF (2023). An influenza H1 hemagglutinin stem-only immunogen elicits a broadly cross-reactive B cell response in humans. Sci Transl Med.

[19] Swart M (2023). Enhancing breadth and durability of humoral immune responses in non-human primates with an adjuvanted group 1 influenza hemagglutinin stem antigen. NPJ Vaccines.

[20] Widge AT (2023). An influenza hemagglutinin stem nanoparticle vaccine induces cross-group 1 neutralizing antibodies in healthy adults. Sci Transl Med.

[21] León AN (2025). Structural mapping of polyclonal IgG responses to HA after influenza virus vaccination or infection. mBio.

[22] Ochsner SP (2021). FcRn-targeted mucosal vaccination against influenza virus infection. J Immunol.

[23] Graves P (1983). Preparation of influenza virus subviral particles lacking the HA1 subunit of hemagglutinin: unmasking of cross-reactive HA2 determinants. Virology.

[24] Steel J (2010). Influenza virus vaccine based on the conserved hemagglutinin stalk domain. MBio.

[25] Lu Y (2014). Production and stabilization of the trimeric influenza hemagglutinin stem domain for potentially broadly protective influenza vaccines. Proc Natl Acad Sci U S A.

[26] Impagliazzo A (2015). A stable trimeric influenza hemagglutinin stem as a broadly protective immunogen. Science.

[27] Yassine HM (2015). Hemagglutinin-stem nanoparticles generate heterosubtypic influenza protection. Nat Med.

[28] Corbett KS (2019). Design of nanoparticulate group 2 influenza virus hemagglutinin stem antigens that activate unmutated ancestor B cell receptors of broadly neutralizing antibody lineages. MBio.

[29] Nimmerjahn F, Ravetch JV (2005). Divergent immunoglobulin g subclass activity through selective Fc receptor binding. Science.

[30] Lu L (2011). A neonatal Fc receptor-targeted mucosal vaccine strategy effectively induces HIV-1 antigen-specific immunity to genital infection. J Virol.

[31] Duncan AR, Winter G (1988). The binding site for C1q on IgG. Nature.

[32] Weldon WC (2010). Enhanced immunogenicity of stabilized trimeric soluble influenza hemagglutinin. PLoS One.

[33] Ekiert DC (2009). Antibody recognition of a highly conserved influenza virus epitope. Science.

[34] Tan GS (2012). A pan-H1 anti-hemagglutinin monoclonal antibody with potent broad-spectrum efficacy in vivo. J Virol.

[35] Friesen RH (2014). A common solution to group 2 influenza virus neutralization. Proc Natl Acad Sci U S A.

[36] Nath Neerukonda S (2020). Neutralizing antibodies targeting the conserved stem region of influenza hemagglutinin. Vaccines (Basel).

[37] Ekiert DC (2011). A highly conserved neutralizing epitope on group 2 influenza A viruses. Science.

[38] Li W (2023). An FcRn-targeted mucosal vaccine against SARS-CoV-2 infection and transmission. Nat Commun.

[39] Tesar DB (2006). Ligand valency affects transcytosis, recycling and intracellular trafficking mediated by the neonatal Fc receptor. Traffic.

[40] Moldoveanu Z (1998). CpG DNA, a novel immune enhancer for systemic and mucosal immunization with influenza virus. Vaccine.

[41] Blaauboer SM (2015). The mucosal adjuvant cyclic di-GMP enhances antigen uptake and selectively activates pinocytosis-efficient cells in vivo. Elife.

[42] Kubo T (2015). CpG-DNA enhances the tight junction integrity of the bronchial epithelial cell barrier. J Allergy Clin Immunol.

[43] Corti D (2011). A neutralizing antibody selected from plasma cells that binds to group 1 and group 2 influenza A hemagglutinins. Science.

[44] Allie SR, Randall TD (2017). Pulmonary immunity to viruses. Clin Sci (Lond).

[45] Turner D (2014). Lung niches for the generation and maintenance of tissue-resident memory T cells. Mucosal Immunol.

[46] Erbelding EJ (2018). A universal influenza vaccine: the strategic plan for the national institute of allergy and infectious diseases. J Infect Dis.

[47] Slifka MK (1998). Humoral immunity due to long-lived plasma cells. Immunity.

[48] Temperton NJ (2007). A sensitive retroviral pseudotype assay for influenza H5N1-neutralizing antibodies. Influenza Other Respir Viruses.

[49] Kallewaard NL (2016). Structure and function analysis of an antibody recognizing all influenza A subtypes. Cell.

[50] Garcia-Moro E (2022). Reversible structural changes in the influenza hemagglutinin precursor at membrane fusion pH. Proc Natl Acad Sci U S A.

[51] Jegaskanda S (2013). Cross-reactive influenza-specific antibody-dependent cellular cytotoxicity antibodies in the absence of neutralizing antibodies. J Immunol.

[52] DiLillo DJ (2014). Broadly neutralizing hemagglutinin stalk-specific antibodies require FcγR interactions for protection against influenza virus in vivo. Nat Med.

[53] de Vries RD (2017). Influenza virus-specific antibody dependent cellular cytoxicity induced by vaccination or natural infection. Vaccine.

[54] Motsoeneng BM (2024). Hemagglutinin stalk-specific fc-mediated functions are associated with protection against influenza illness after seasonal influenza vaccination. J Infect Dis.

[55] Nachbagauer R (2021). A chimeric hemagglutinin-based universal influenza virus vaccine approach induces broad and long-lasting immunity in a randomized, placebo-controlled phase I trial. Nat Med.

[56] Edgar JE (2023). Antibodies elicited in humans upon chimeric hemagglutinin-based influenza virus vaccination confer FcγR-dependent protection in vivo. Proc Natl Acad Sci U S A.

[57] Terajima M (2011). Complement-dependent lysis of influenza a virus-infected cells by broadly cross-reactive human monoclonal antibodies. J Virol.

[58] He W (2017). Alveolar macrophages are critical for broadly-reactive antibody-mediated protection against influenza A virus in mice. Nat Commun.

[59] Mullarkey CE (2016). Broadly neutralizing hemagglutinin stalk-specific antibodies induce potent phagocytosis of immune complexes by neutrophils in an Fc-dependent manner. MBio.

[60] Ackerman ME (2011). A robust, high-throughput assay to determine the phagocytic activity of clinical antibody samples. J Immunol Methods.

[61] Tan H-X (2019). Subdominance and poor intrinsic immunogenicity limit humoral immunity targeting influenza HA stem. J Clin Invest.

[62] Nagashima KA, Mousa JJ (2021). Next-generation influenza HA immunogens and adjuvants in pursuit of a broadly protective vaccine. Viruses.

[63] Renegar KB (2004). Role of IgA versus IgG in the control of influenza viral infection in the murine respiratory tract. J Immunol.

[64] Bai Y (2011). Intracellular neutralization of viral infection in polarized epithelial cells by neonatal Fc receptor (FcRn)-mediated IgG transport. Proc Natl Acad Sci U S A.

[65] Tete SM (2016). Dissecting the hemagglutinin head and stalk-specific IgG antibody response in healthcare workers following pandemic H1N1 vaccination. NPJ Vaccines.

[66] Nachbagauer R (2019). Pandemic influenza virus vaccines boost hemagglutinin stalk-specific antibody responses in primed adult and pediatric cohorts. NPJ Vaccines.

[67] Dugan HL (2020). Preexisting immunity shapes distinct antibody landscapes after influenza virus infection and vaccination in humans. Sci Transl Med.

[68] Joyce MG (2016). Vaccine-induced antibodies that neutralize group 1 and group 2 influenza A viruses. Cell.

[69] Subbiah J (2022). A chimeric thermostable M2e and H3 stalk-based universal influenza A virus vaccine. NPJ Vaccines.

[70] Houser K, Subbarao K (2015). Influenza vaccines: challenges and solutions. Cell Host Microbe.

[71] Khurana S (2013). Vaccine-induced anti-HA2 antibodies promote virus fusion and enhance influenza virus respiratory disease. Sci Transl Med.

[72] Epstein SL (2018). Universal influenza vaccines: progress in achieving broad cross-protection in vivo. Am J Epidemiol.

[73] Sui J (2009). Structural and functional bases for broad-spectrum neutralization of avian and human influenza A viruses. Nat Struct Mol Biol.

[74] Vidarsson G (2006). FcRn: an IgG receptor on phagocytes with a novel role in phagocytosis. Blood.

[75] Baker K (2011). Neonatal Fc receptor for IgG (FcRn) regulates cross-presentation of IgG immune complexes by CD8-CD11b+ dendritic cells. Proc Natl Acad Sci U S A.

[76] Liu X (2011). The neonatal FcR-mediated presentation of immune-complexed antigen is associated with endosomal and phagosomal pH and antigen stability in macrophages and dendritic cells. J Immunol.

[77] Chen Y-Q (2019). Hemagglutinin stalk-reactive antibodies interfere with influenza virus neuraminidase activity by steric hindrance. J Virol.

[78] Kosik I (2019). Neuraminidase inhibition contributes to influenza A virus neutralization by anti-hemagglutinin stem antibodies. J Exp Med.

[79] Zhang A (2022). Hemagglutinin stalk-binding antibodies enhance effectiveness of neuraminidase inhibitors against influenza via Fc-dependent effector functions. Cell Rep Med.

[80] Sanchez-de Prada L (2023). Group 1 and group 2 hemagglutinin stalk antibody response according to age. Front Immunol.

[81] Johansen ND (2025). High-dose influenza vaccine effectiveness against hospitalization in older adults. N Engl J Med.

[82] Aydillo T (2020). Pre-existing hemagglutinin stalk antibodies correlate with protection of lower respiratory symptoms in flu-infected transplant patients. Cell Rep Med.

[83] Yassine HM (2018). Use of Hemagglutinin Stem Probes Demonstrate Prevalence of Broadly Reactive group 1 Influenza Antibodies in Human Sera. Sci Rep.

