# Supplementary material for: Mucosal vaccination with hemagglutinin stalk provides broad protection against influenza virus subtypes in mice
Source: J Clin Invest. 2026 Nov 16;136(22):e203675. doi: 10.1172/JCI203675 (PMC13767620; doi:10.1172/JCI203675)
Supplement: Supplementary file 1 [file jci-136-203675-s092.pdf]

Supplemental Methods

Cells, viruses, and proteins. Chinese hamster ovary (CHO) cells, Vero cells, J774, L929, and Madin-Darby canine kidney (MDCK) cells were obtained from ATCC (Manassas, VA). All cell types were maintained in complete DMEM supplemented with 10% fetal bovine serum (FBS), 2 mM L-glutamine, non-essential amino acids, and antibiotics (100 units/ml of penicillin, 100 µg/ml of streptomycin, and 250 ng/ml of amphotericin B). The stably transfected CHO cells were cultured in complete medium containing G418 at a concentration of 1 mg/ml. All cells were grown at 37°C in a 5% CO_2 atmosphere. FTY720, sphingosine 1-phosphate receptor-1 agonist, was from Cayman Chemical (Ann Arbor, Michigan, Cat# 10006292)

Influenza virus strains used in this study include: PR8 (A/Puerto Rico/8/1934, H1N1), A/Philippines/2/82 X-79 (H3N2, ID: C6154), ΔH5N1 (A/Vietnam/1203/2004)/PR8 (a reassortant virus with HA and NA genes from A/Vietnam/1203/2004 (H5N1), where the multi-basic amino acids in the cleavage site of HA are deleted, along with six internal genes from PR8, ID: SJRG-161052), H6N2HK/14 (a reassortant virus with the HA gene from A/turkey/Massachusetts/3740/1965 (H6N2), the NA gene from A/Hong Kong/4801/2014 (H3N2), and six internal genes from PR8),

A/Bangladesh/0994/2011 (H9N2)/PR8 (a reassortant having HA and NA genes from A/Bangladesh/0994/2011 (H9N2) and six internal genes from PR8, ID: CDC-RG31), and A/Chicken/Hong Kong/G9/1997 (H9N2), which is a filed strain. The virus names were abbreviated as H1N1, H3N2, rH5N1, rH6N2, rH9N2, and H9N2, respectively. All viruses were propagated in 10 to 11-day-old embryonated eggs. Virus titers were determined in MDCK cells and were expressed as $TCID_{50}$ (50% tissue culture infective dose) using the method described by Reed and Muench.

The HA proteins used in this research were obtained from BEI Resources (Manassas, VA). They include HA proteins from A/California/07/2009 (H1N1, NR-51668), A/Singapore/1/1957 (H2N2, NR-52249), A/chicken/Netherlands/14015531/2014 (H5N8, NR-50133), A/turkey/Wisconsin/1/1966 (H9N2, NR-43782), A/Hong Kong/308/2014 (H9N2,

NR-51861), A/laughing gull/Delaware Bay/451/2018 (H13N6, NR-58707), and A/mallard/Alberta/455/2015 (H4N6, NR-51128).

Constructions of plasmids. A pcDNA3.1-Stalk-Fc/wt plasmid, which encodes the CHO-optimized Stalk-Fc/wt sequence, was synthesized by GenScript (Piscataway, NJ). To construct a plasmid expressing Stalk-Fc/mut, an inverse PCR was performed using pcDNA3.1-Stalk-Fc/wt as a template along with the primer pairs: Fc310/311-F: 5'-CCAATCCAGGCCGACGACTGGATGAGCGGCAAGG CC-3' and Fc310/311-R: 5'-CATCCAGTCGTCGGCCTGGATTGGCAGGGCGGACAC-3'. This method introduced HQ/AD mutations at positions 310 and 311 in the IgG Fc portion. Following this, a second round of PCR was conducted using the primer pairs Fc433/434-F: 5'-GAGG GCCTGGCCCAGCACCATACCACAAAGAGCTTT-3' and Fc433/434-R: 5'-GGTATGGTGCTGGGCCAGGCCCTCGTGCACCACGGA-3'. The PCR products were transformed into DH5α cells, and pcDNA3.1-Stalk-Fc/mut was screened from ampicillin agar plates. A similar approach was used to generate the pcDNA3.1-Stalk plasmid, utilizing the primer pairs: 5'-CACATTTCTG GGACATCATCACCATCACCATCACCATTGACTCGAGTCTAGAGG GCCCG-3' and 5'-ATGGTGATGGTGATGGTGATGATGTCCCAGAAATGTGCTCAGCAGCACCCACTCG CCATC-3'. The pcDNA3.1-Stalk plasmid encodes a Stalk protein fused with a foldon domain and an 8x His tag at the C-terminus for purification purposes. The plasmids pcDNA3-HA1 or pcDNA3-HA5 were created by inserting the extracellular domain of the HA gene from the PR8 or A/Vietnam/1203/2004 (H5N1) viruses, fused with a segment containing foldon and a 6 x His tag, into the pcDNA3 vector. The *Kpn* I and *Not* I sites and the *Kpn* I and *Xba* I sites were utilized for inserting the fused cDNA fragments, respectively. To modify the HA1 gene's cleavage site, the NPQRETRG sequence was used to replace the original IPSIQSRG sequences. For the HA5, the cleavage site was abolished by replacing the QRERRRKKRG sequence with QRETQG mutations. To produce a pseudotyped H5N1 reporter virus, the full-length HA gene lacking the polybasic cleavage site from the A/Vietnam/1203/2004 (H5N1) virus and the NA segment from A/mallard/Sweden/81/2002 (H6N1) were amplified. This was done using the H5 reverse genetic plasmid (a gift from Dr. Shin-Hee Kim) and the

NA reverse genetic plasmid (BEI Resources, Cat# NR-29010) as templates and subsequently cloned into the pcDNA3 backbone. Lastly, the pcDNA3-TMPRSS2 plasmid was constructed by cloning human transmembrane serine protease 2 (TMPRSS2) cDNA into the pcDNA3 vector. The fidelity of all plasmids was verified through sequencing.

Expression and purification of Stalk-Fc and Stalk proteins. The plasmids pcDNA3.1-Stalk-Fc/wt, pcDNA3.1-Stalk-Fc/mut, and pcDNA3.1-Stalk were each individually transfected into CHO cells using PEI MAX 40000 (Fisher Scientific, Cat# NC1038561). Stable clones were selected using G418 at a concentration of 1 mg/ml, and the presence of secreted proteins was confirmed by Western blot analysis. A complete DMEM medium with 5% ultra-low IgG FBS was used to grow the CHO clones expressing the Stalk-Fc/wt protein. In contrast, CHO clones expressing Stalk-Fc/mut or Stalk proteins were cultured in complete DMEM medium containing 5% regular FBS. The Stalk-Fc/wt proteins in the supernatants were purified using a Protein A affinity column (ThermoFisher Scientific, Cat# 20356). The Stalk-Fc/mut protein was purified with an anti-mouse IgG agarose column (Rockland, Cat# 210-1150), while the Stalk protein was purified using an anti-His resin affinity column (R&D Systems, Cat# IP999). The affinity columns were washed with two column volumes of PBS and eluted with 0.1M glycine (pH 2.5), followed immediately by neutralization with 1M Tris-HCl (pH 8.0). To remove glycine and Tris-HCl, the purified protein solutions were exchanged into PBS using Amicon Ultra-15 Centrifugal Filter Units (50 K) (Millipore, Cat# UFC905024).

HA1 and HA5 proteins were produced and purified using the same method as the Stalk protein. The SARS-CoV-2 Spike protein was produced as previously described (1). Protein concentrations were measured using a NanoDrop spectrophotometer (Thermo Scientific).

SDS-PAGE and Western blotting. The purified proteins were subjected to various conditions, such as exposure to different temperatures for a specific duration or repeated freeze/thaw treatments. For comparison, protein stored at -80°C was used as a control. These proteins were then mixed with reducing Laemmli buffer. After heating the samples at 97°C for 5 min, they were separated using a 12% SDS-polyacrylamide gel and transferred

to a 0.45 μm nitrocellulose membrane. The membrane was blocked in PBS buffer containing 5% nonfat milk for 1 h, followed by overnight incubation at 4°C with the appropriate Abs. After washing 3 times with PBS, the membrane was exposed to HRP-conjugated secondary antibodies for 1.5 h at room temperature (RT). Finally, the protein bands were visualized using Immobilon Western chemiluminescent substrate (Millipore, Cat# WBKLS0100), and images were captured using a Chemi Doc XRS system (BioRad).

Surface Plasmon Resonance (SPR) analysis. The affinities of Stalk-Fc/wt with mouse FcRn/β2m or FcγRI were assessed using a Biacore instrument at ACROBiosystems. Mouse FcRn/β2m (ACROBiosystems, Cat# FCM-M52W8) was diluted with 10 mM sodium acetate (pH 4.5) and immobilized on a CM5 biosensor chip using an amine coupling kit, achieving a strength of 200 resonance units (RU). Stalk-Fc/wt was diluted in the running buffer (PBS with 0.05% Tween-20, pH 6.0) to a concentration ranging from 15.625 nM to 1000 nM and injected at 25°C. The buffer was run at a flow rate of 30 μl/min, allowing for an association time of 60 s and a dissociation time of 90 s. Regeneration of the sensor surfaces was performed by injecting a regeneration buffer (HEPES with 0.005% Tween-20, pH 7.4) at a flow rate of 20 μl/min for 30 s.

The CM5 chip was immobilized with mouse FcγRI (ACROBiosystems, Cat# CD4-M5227), which was diluted in an immobilization buffer containing 10 mM sodium acetate at pH 5.5, achieving a loading of 100 RU. Various concentrations of Stalk-Fc/wt proteins, ranging from 3.906 nM to 1000 nM, were then injected using a running buffer composed of 1× HEPES, 50 μM EDTA, and 0.005% Tween-20. The injection was performed at a flow rate of 30 μl/min, allowing for association and dissociation times of 60 s and 90 s, respectively. To regenerate the immobilized proteins, a regeneration buffer containing 3M $MgCl_2$ was injected for 60 s at the same flow rate of 30 μl/min. The kinetic constants were determined from the sensorgrams using the 1:1 binding model in BIA Evaluation Software 4.1.

Mice immunization, sample collection, and influenza A virus challenges. The 7- to 8-week-old C57BL/6 male/female mice or FcRn knockout (KO) mice, bred on a C57BL/6 background, were divided into five groups: Stalk-Fc/wt, Stalk-Fc/wt/KO, Stalk-Fc/mut, Stalk, and PBS groups. Each group of mice was intranasally (i.n.) immunized with 20 μl of

either 10 μg Stalk-Fc or 5.5 μg Stalk (the equal molar ratio), combined with 10 μg CpG 1826 (InvivoGen, CA) or 5 μg C-di-GMP (InvivoGen, CA) as an adjuvant, under light to moderate anesthesia using Avertin. The mice received two booster doses at three-week intervals. 2 to 3 weeks after the final boost, sera, nasal washes, and bronchoalveolar lavage (BAL) samples were collected. For intramuscular (i.m.) immunizations, each mouse received a 100 μl mixture of HA proteins and CpG/Alum administered in the thigh muscles of the hind limb. This mixture consisted of 10 μg HA1, 10 μg HA3, and 10 μg CpG in a total volume of 50 μl, which was then combined with 50 μl of Imject Alum (ThermoFisher Scientific).

For nasal wash sampling, the mouse was euthanized, and a small hole was made in the trachea. A 1-ml syringe with a plastic tip was inserted into the trachea toward the posterior opening of the nose. A total of 2 ml of PBS was injected into the nasal cavity and collected through the external nostrils. For the BAL collection, the syringe tip was guided toward the lungs via the trachea, and 1 ml of PBS was injected to flush the bronchi and alveoli to obtain the lavage. This process was repeated once, and the lavages from each mouse were combined. The nasal washes or BAL fluids were clarified by centrifugation and concentrated to 350 μl using an Amicon 0.5 ml centrifugal filter unit (10 K) (Millipore, Cat# UFC501096).

To infect the mice, they were fully anesthetized via intraperitoneal injection of Avertin. A lethal dose of rH5N1 ($5x10^2$ $TCID_{50}$), H1N1 ($3x10^3$ $TCID_{50}$), rH6N2 ($4x10^4$ $TCID_{50}$), or H3N2 ($5x10^3$ $TCID_{50}$) in volume of 50 μl, was slowly administered into their nostrils. A field strain of H9N2 virus was inoculated at a high dose ($1x10^7$ $TCID_{50}$) since it is not adapted in mice and not lethal for mice. Body weight changes, physical appearance, and clinical signs were monitored daily for 14 days. Mice that lost more than 25% of their initial body weight were euthanized immediately.

Enzyme-linked immunosorbent assay (ELISA) and competitive ELISA. The ELISA assays were performed following previously described procedures (1, 2). To assess the binding of Stalk-Fc to FcRn or FcγRI receptors, Stalk-Fc proteins were coated onto an ELISA plate at a concentration of 300 ng per well. A 2-fold serial dilution of mouse FcRn/β2m heterodimer (ACROBiosystems, Cat# FCMM82W6) in PBS at pH 6.0 or pH 7.4, or mouse FcγRI

(ACROBiosystems, Cat# CD4-M82E7) in PBS at pH 7.4, was then added to the plate and incubated at RT for 2 h. To evaluate the binding of Stalk-Fc to complement, Stalk-Fc proteins were coated onto plates through a 2-fold serial dilution, starting at 800 ng per well. Mouse C1q (Complement Technology, Cat# M099) was biotin-conjugated and used for detection at a concentration of 2 μg/ml.

To verify that the recombinant Stalk proteins with or without Fc were properly folded, Stalk-specific monoclonal antibodies (mAbs), including KB2, 6F12, CR6261, CR8020, and FI6V3 (kindly provided by Dr. Kramer Florian and Dr. Jeffery Boyington, NIH), were utilized. These mAbs were labeled with biotin and added to the plate in various concentrations ranging from 0.25 to 0.5 μg/ml. Additionally, a rabbit polyclonal Ab against the HA2 portion of HA (Biorbyt, Cat# orb10765, diluted 1:5000), along with an HRP-anti-rabbit secondary Ab (Southern Biotech, Cat# 4030-05,1:10,000), was included in the conformational tests. Biotin-conjugated proteins were visualized using HRP-Streptavidin (Pierce, Cat# 21130,1:30,000) and TMB (tetramethylbenzidine) substrate (BD Bioscience, Cat# 555214). Color development was stopped by adding 100 μl of 1M sulfuric acid, and the plates were read at 450 nm using a Victor III microplate reader (Perkin Elmer).

To detect HA- or Stalk-specific Abs in mouse sera, nasal washes, and BAL, the 96-well ELISA plates (Maxisorp, Nunc) were coated with 100 ng of the Stalk protein in 100 μl of coating buffer (PBS, pH 7.4) per well and incubated at 4°C overnight. After washing the plates 4 times with PBST (0.05% Tween 20 in PBS), the plates were blocked with a blocking buffer (2% BSA in PBST) at RT for 1 to 2 h. Next, the sera, nasal washes, or BAL samples were added to the plates in two-fold serial dilutions and incubated for 2 h at RT. Followed by six washes with PBST, the plates were then incubated with appropriate detection reagents for 1.5 h. For measuring Stalk-specific IgG, the HRP-conjugated rabbit anti-mouse IgG (1:20,000, Invitrogen, Cat# PA1-28568) was used. For Stalk-specific IgA detection, the biotin-labeled goat anti-mouse IgA (1:5000, Southern Biotech, Cat# 1040-08), along with HRP-Streptavidin (1:7000, Southern Biotech, Cat# 7100-05), were used. After thoroughly washing, 100 μl of TMB substrate was added to each well. The color reactions were stopped by adding 100 μl of 1M sulfuric acid, and the absorbance at 450 nm was measured

using a microplate reader. The titers were determined as the highest dilution of samples showing a two-fold increase over the average OD450 nm values of the blank controls.

Serum from both immunized and controlled animals was tested for Abs that compete with HA Stalk-specific mAbs KB2, 6F12, and FI6V3. The ELISA plates were coated with 100 µl of HA protein from the A/California/07/2009 (H1N1) virus, incubated overnight at 4°C. To optimize binding, the plates were coated with Stalk protein in varying amounts of 12.5 ng, 50 ng, and 100 ng per well. After 24 h, the plates were blocked with 2% BSA in a PBST buffer for 2 h. Serially diluted mouse sera, starting from 1:30, were added to the plates and incubated at RT for 2 h. Following this, biotin-conjugated KB2, 6F12, or FI6V3 mAbs were added to the wells at dilutions of 1:500, 1:200, and 1:25,000, respectively, and allowed to incubate for an additional 2 h. After thorough washing, HRP-Streptavidin (Pierce, Cat# 21130, 1:30,000) was added for 1 h. TMB substrate was then added, and the colorimetric change was measured at OD450 nm. The competition percentage was calculated and represented as the reduction of mean absorbance in the presence of serum IgG.

***Microneutrilization (MN) assay using pseudovirus*.** To measure serum MN Ab titer, lentivirus particles were produced, which were pseudotyped with HA from the H5N1 subtype (A/Vietnam/1203/2004) and NA from the H6N1 subtype (A/mallard/Sweden/81/2002). To make this pseudovirus, 1 µg of psPAX2 plasmid for gag-pol expression, 1.5 µg of luciferase reporter plasmid (pLenti CMV V5-luc Blast), 1 µg of pcDNA3-HA5, 0.25 µg of pcDNA3-NA1, and 1 µg of pcDNA3-TMPRSS2 were mixed with a 29 µl of PEI-Max transfection reagent (Kyfora Bio LLC) in serum-free Opti-MEM. After a 20-minute incubation at RT, 5 mL of 293T cells (2 x 10^6 cells/ml) in complete DMEM media were added, and the mixture was then seeded into three wells of a 6-well plate and incubated for 12 h at 37°C. The media were then replaced with complete DMEM media containing 200 mM sodium butyrate. After 12 h of incubation, the media were replaced with serum-free DMEM supplemented with *vibrio cholerae* sialidase (Sigma, Cat # 11080725001, 10 µ/ml), and further incubated for 36 h at 37°C. Finally, the supernatants were harvested, centrifuged at 3900 rpm for 15 min, and stored at -80°C. Titration of the

pseudovirus was performed on 293T cells in a 96-well plate by adding a 2-fold serial dilution of the supernatant in DMEM. The relative luminescence units per milliliter (RLU/mL) were measured using a plate reader.

Mouse serum samples, treated with RDE and inactivated at 56°C for 30 min, were serially diluted 2-fold in DMEM and mixed with an equal volume of diluted H5N1 pseudovirus, which was adjusted to yield luciferase activity within the linear range. After incubation at 37°C for 1 h, 100 µl of the serum and pseudovirus mixture was added to 50 µl of 4 x 10^4 293T cells in complete DMEM containing 3 µg/ml polybrene. The final mixture was immediately transferred into 96-well plates, and after 8 h, 100 µl of fresh media was added to each well. After 16 h, the supernatants in each well were replaced with complete DMEM. The luciferase activity was evaluated 48 h after the pseudovirus infection using Bright-Glo reagent (Promega, Cat # E2610). The micro-neutralizing titer was defined as the ID_{50} (the dilution factor causing 50% inhibition), calculated by considering the cells infected with the pseudovirus as 0% and the uninfected cells as 100%.

Enzyme-linked Immunospot (ELISpot) assay. The ELISpot assay was conducted to measure the cytokines secreted by lymphocytes. To achieve this, spleens were isolated 2 weeks after the last boost. Single cells were harvested by abrading the tissue over a 40 µm cell strainer. Red blood cells (RBCs) were lysed using 2.5 ml of ACK lysis buffer. The 96-well ELISpot PVDF plates (MabTech, Cat# 3654-TP-10) were pre-wetted with 35% ethanol and then thoroughly washed six times with sterile H2O. Next, the plates were coated with 100 µl of Abs (20 µg/ml) specifically against mouse IFN-γ (MabTech, Cat# 3321-3-250), TNF-α (MabTech, Cat# 3511-3-250), or IL-4 (MabTech, Cat# 3311-3-250) overnight at 4°C. The following day, the plates were washed five times with PBS and blocked using RPMI medium containing 10% FBS at RT for 2 h. Spleen cells were then added at two different numbers: 5 x 10^5 cells/well or 1.25 x 10^5 cells/well. The plates were incubated at 37°C for 40 h and then rinsed with PBS.

Biotin-conjugated Abs specific for mouse IFN-γ, TNF-α, or IL-4 (MabTech, Cat# 3321-6-250, 3511-6-250, and 3311-6-250) were added at concentrations of 1, 0.5, and 1.5 µg/ml, respectively, in PBS containing 0.5% FBS for 2 h at RT. Following PBS washing, HRP-

streptavidin (100 µl/well, 1:700) was added, and the plates were incubated for 1 h. After the final PBS wash, TMB substrate was added, leading the spots to appear over time. The color development was stopped by emptying the plates and washing them extensively with deionized water.

ELISpot was also used to determine antibody-secreting cells (ASC). An IgA or IgG kit (MabTech, Cat# 3865-2H and 3825-2H) was utilized. Briefly, single cells were collected from the femurs and tibias of mice at 10 or 16 months after the final boost. The 96-well PVDF plates were pretreated with 35% ethanol and coated with 100 µl of anti-mouse IgG or IgA antibody (15 µg/ml). Each well was loaded with 1 x 10^6 bone marrow cells, except for the wells in the Stalk-Fc/wt group for the IgG-ASC examination, which used fewer cells (2.5 x 10^5 cells/well) to avoid overlap and conflation of spots. Biotin-labeled Stalk protein (1 µg/ml), HRP-streptavidin (1:500), and TMB were then applied to develop the spots. For both experiments, the PVDF was air-dried overnight, and the number of spots was counted using an ELISpot reader (AID, Germany).

Fluorophore conjugation of antigens. To label antigens, Stalk-Fc or Stalk alone was fluorescently labeled using an Alexa Fluor 647 Microscale Protein Labelling Kit (Invitrogen, Cat# A30009). In brief, the concentration of Stalk-Fc or Stalk proteins was adjusted to 1mg/ml. 1 M sodium bicarbonate was added at 1/10 volume and followed by mixing with Alexa Fluor 647 dye at a molar ratio of 1:28. After incubating 15 min at RT, the conjugate was centrifuged for 1 min at a speed of 16,000 g in a spin column to remove the unreacted dye, and then measured at absorbance at 280 nm (A280) and 650 nm (A650) by a NanoDrop spectrophotometer. Degree of labeling (DOL) was measured and calculated using an equation: DOL= (moles dye)/ (moles protein) = A650 × dilution factor / (239,000 × protein concentration (M)). Ideally, a DOL should be between 5-8.

In vitro and in vivo transcytosis assays. The evaluation of FcRn-mediated Stalk-Fc transcytosis was conducted using MDCK (rFcRn/rβ2m) cells (3) grown on transwell inserts with a 0.4 µm pore size (Corning, Cat# 3413). Cells were seeded at a density of 1.2 × 10^5 cells per well in Opti-MEM media supplemented with 10% low-IgG FBS. Once the cells became fully confluent, achieving a trans-epithelial electrical resistance (TEER) of 250-300

Ω*cm^2, the media were replaced. In the inner chamber, 200 µl of low pH buffer (Opti-MEM/2% low-IgG FBS/20 mM MES, pH 6.0) was added, while the outer chamber received 600 µl of neutral pH buffer (Opti-MEM/2% low-IgG FBS/20 mM HEPES, pH 7.4). After allowing the system to equilibrate for 20 min at 37 °C, the inner chamber buffer was replaced with 200 µl of proteins diluted in the prewarmed low pH buffer at a concentration of 50 µg/ml for Stalk-Fc/wt or Stalk-Fc/mut, and 27.5 µg/ml for Stalk. The transwell inserts were then placed into new wells containing 600 µl of fresh neutral pH buffer for an additional 90 min of incubation at 37 °C. After incubation, the transported proteins in the outer chamber were collected and quantified using ELISA. To detect Stalk-Fc and Stalk, the microplate was coated overnight at 4 °C with 100 µl of FI6V3 mAb at a concentration of 1µg/ml. Biotin-conjugated goat anti-mouse IgG2a (1:4000, Southern Biotech, Cat# 1080-08) and Biotin-conjugated anti-His Ab (1:4000, GenScript, Cat# A00613), along with HRP-Streptavidin (1:5000, Biolegend, Cat# 405210), were used to measure Stalk proteins with and without the Fc-fusion. The four-parameter logistic (4-PL) curves for Stalk-Fc/wt, Stalk-Fc/mut, and Stalk proteins were plotted using online software provided by AAT Bioquest. The amount of unknown protein was calculated based on these curves.

To examine the protein transported into the nasal tissues, the C57BL/6 mice and FcRn KO mice were i.n. administered with 10 µg of AF647-labeled proteins and collected 7 h following the delivery under anesthetization. Heads were removed and fixed in 4% paraformaldehyde overnight at 4°C. After fixation, the nasal tissues were decalcified with TBD-1 solution (Thermo Scientific, Cat# 6764001) at 4°C for 2 h, followed by cryoprotection in 30% sucrose overnight at 4°C. The samples were embedded in optimal cutting temperature (OCT) compound (Fisher Healthcare, Cat# 23730571). The frozen samples were sectioned with a Microm HM 550 cryostat (Thermo Scientific) along the coronal plane in 7 µm sections. The slides (Fisherbrand Superfrost Plus, Cat# 22037246) were air dried and quenched with 0.05M Tris-HCl (pH 7.4) for 30 min at RT. Finally, after washing, the samples were DAPI-stained before imaging using an LSM 800 confocal microscope (Zeiss).

Flow cytometry. Analysis of tissue-resident memory T cells (TRM) in the lungs was performed using flow cytometry, following a previously described method with

modifications (2, 3). Briefly, to distinguish TRM in the lungs from T cells in the bloodstream, mice were injected intravenously with 3 μg of PE-CD45 Ab (Biolegend, Cat# 103106) in 100 μl of PBS at the retro-orbital site. After 5 min, the mice were euthanized, and the lungs were removed and treated with a digestion buffer (RPMI with 1 mg/ml of collagenase IV, 5 mM of $CaCl_2$, 10 μg/ml DNase I) for 1 h at 37°C. Red blood cells were lysed with ACK buffer. Mononuclear cells were further enriched by centrifuging the cell suspensions in 37% Percoll. The cells were stimulated with pooled peptides from H1N1 virus HA (Miltenyi Biotec, Cat#130-099-803) in the presence of CD28 Ab (Biolegend, Cat# 102116) and CD49d Ab (Biolegend, Cat# 103710) for 2 h at 37°C followed by addition of GolgiPLug (BD, Cat# 555029) and continued culturing for additional 6 h. Then, the cells were washed with PBS, stained with Fixable Live/Dead Aqua staining dye (Invitrogen, Cat# L34966) at RT for 30 min and blocked with FcR block (anti-mouse CD16/CD32, BD Biosciences, Cat# 553142) in FACS buffer (2% FBS and 2 mM EDTA in PBS) at 4°C for 30 min. Subsequently, the surface staining of cells was performed at 4°C for 45-60 min by using the following mAbs: FITC-CD3 Ab (BD, Cat# BDB555275), APC-H7-CD4 Ab (BD, Cat# 590181), BV785-CD8a Ab (Biolegend, Cat# 100750), PerCP-Cy5.5-CD44 Ab (Biolegend, Cat#103032), AF700-CD62L (Biolegend, Cat#104426), APC-CD69 Ab (BD, Cat# BDB560689), and PE-eFluor610-CD103 Ab (eBioscience, Cat# 61103182, abbreviated as PE610-CD103). The cells were further washed with PBS, fixed and permeabilized with Cytofix/CytoPerm solution (BD, Cat# 554722) for 20 min at 4°C, and subjected to intracellular staining with BV421-IFN-γ Ab (Biolegend, Cat# 505830), BV650-TNF-α Ab (Biolegend, cat# 506333), and BV605-IL-2 Ab (Biolegend, Cat# 563911). The phenotyping was conducted using a FACS Celesta cytometer (BD Biosciences). Abc beads (Invitrogen, Cat# A10497) and Arc beads (Invitrogen, Cat# A10346) were used to create compensation controls. Data analysis was performed using the FlowJo software (Tree Star).

Measurement of antigen uptake. To investigate the effects of Fc-fusion on HA Stalk uptake by macrophages or dendritic cells, we conducted an FcR engagement test. A murine macrophage cell line, J774, with 7.5×10^5 cells per well, was seeded into a 12-well plate and incubated at 37°C for 24 h until reaching 95% confluence. Certain wells received

normal mouse IgG (50 µg IgG in 500 µl serum-free DMEM), while others were supplied with media only. For the preparation of bone marrow-derived dendritic cells (BMDCs), bone marrow cells isolated from C57BL/6 or FcRn KO mice were seeded into a 10-cm non-tissue culture-treated petri dish containing complete RPMI media supplemented with 20 ng/ml mouse GM-CSF (Biolegend, Cat# 576302). The media were changed every 2-3 days, and after 7-9 days, the floating and loosely attached cells were harvested. BMDCs (1.5×10^6 cells/well) were transferred to a 96-well V-bottom plate. AF647-labeled Stalk proteins (1.5 µg for Stalk-Fc/wt and Stalk-Fc/mut, and 0.84 µg for Stalk) were added to each well. To analyze the AF647-positive cells, either J774 cells or BMDCs were harvested after 25-minute incubation at 37 °C. Flow cytometry was performed following Live/Dead aqua staining for J774 cells and Live/Dead aqua plus BV785-CD11c Ab (Biolegend,117336) staining for BMDC cells. Data were analyzed using FlowJo and plotted as an overlay histogram. The mean fluorescence intensity (MFI) was calculated and normalized by dividing the raw MFI value by the DOL value, allowing for the meaningful comparison of protein uptake among different groups.

Virus titration. Mice were infected with the influenza A virus and euthanized at 4 day-post—infection (dpi). The lung and nasal turbinate were collected, minced into small pieces with scissors, and placed into 2 ml round-bottom tubes containing serum-free Opti-MEM along with two grinding balls. Homogenization was performed using a TissueLyser LT (Qiagen) at high-speed oscillation (50 Hz). 3-5 mins of homogenization were used for the lungs or nasal turbinate to release the virus, respectively. The samples were clarified via centrifugation and then diluted in media through 10-fold serial dilutions before being used to infect MDCK cells in a 96-well plate. After 1 h incubation at 37°C, the supernatants in each well were removed, and Opti-MEM media containing 1 µg/ml TPCK-trypsin was added. After 3 days of incubation, the presence of the virus was verified by measuring the hemagglutination activity in the supernatant, and the virus titer was calculated and presented as $TCID_{50}$/ml using the Reed-Muench method.

Cell-cell fusion inhibition assay. Vero cells in 96-well plates were infected with various strains of influenza viruses, including H1N1, rH5N1, rH6N2, rH9N2, or H3N2. After 12 h of

infection, cells were washed with PBS and treated with TPCK-trypsin at a concentration of 5 μg/ml for 7 min. Pooled sera were diluted 10-fold and added to the plates, and incubated for 1.5 h. To induce cellular membrane fusion, the cells were incubated for 10 min with PBS (pH 5.0), while PBS at pH 7.2 was used as a control. After washing, the cells were incubated for 3 h at 37°C, followed by fixation with 4% PFA and staining with NP-specific Ab and DAPI. The formation of syncytia and/or clustering of nuclei due to cellular membrane fusion was visualized using fluorescence microscopy.

ADCC, ADCP, and CDC assay. Antibody-dependent cellular cytotoxicity (ADCC) assay was performed using a mouse FcγRIV Reporter kit (Promega, Cat# M1201). Briefly, MDCK cells (5 x 10^4 cells/well) were seeded on a white flat-bottom 96-well plate. The cells were cultivated at 37°C for 18 h and were then infected with influenza A virus (MOI=3) for an additional 14 h. After washing, 25 μl of assay buffer (RPMI with 4% Low-IgG FBS) was added to each well, followed by adding 25 μl 4-fold serially diluted mouse sera (1: 3.33 starting dilution). After adding 25 μl of Jurkat cells (7.5 x 10^4/well) stably expressing mouse FcγRIV, the plates were incubated at 37 °C for 6 h. Subsequently, the Bio-Glo luciferase substrate (75 μl/well) was added and incubated for an additional 25 min at RT in the dark. Luminescence was read using a SpectraMax iD3 plate reader. Each diluted serum sample was tested in duplicates. Data were presented as a mean fold change of RLU (relative luminescence units) = RLU in each serum dilution / RLU in the absence of serum.

Antibody-dependent cellular phagocytosis (ADCP) assay was conducted using a bead-based approach (4). HA proteins (HA1, HA3, HA4, HA5, HA6, HA9) from various subtypes of influenza A viruses were biotinylated using the EZ-link Sulfo-NHS-LC-Biotin kit (Thermo Scientific, Cat# A39257). The biotinylated HA proteins were then coated onto yellow/green NeutrAvidin beads (Invitrogen, Cat# F8776) by mixing 25 μl of beads with 20 μg of Biotin-HA protein in 800 μl of PBS at 37°C overnight. After three washing steps, the Biotin-HA beads were resuspended in 800 μl of PBS containing 0.1% BSA. Phagocytosis was assessed in J774, a macrophage cell line, or bone marrow-derived macrophages (BMDMs). BMDMs were isolated from the femur and tibia of C57BL/6 mice by using complete DMEM/F12-10 media supplemented with 20% of L-929 cell supernatant. Briefly,

10 μl of Biotin-HA-beads (3×10^6) were mixed with 10 μl of the immune serum in 80 μl of PBS and incubated for 2 h with gentle shaking at 37°C. Unbound Abs were thoroughly washed away with cold PBS. The J774 or BMDM cells, cultured to 80-90% confluence in 96-well plates, were precooled at 4°C for 10 min. The Biotin-HA-beads (3×10^6) were then added to the precooled J774 or BMDM cells and incubated for 30 min at 4°C. Following this, the unbound beads were completely washed away with cold PBS, and the plates were incubated at 37°C for 1 h to allow for phagocytosis. Cells were then fixed with 4% PFA and stained with DAPI. To measure bead uptake in J774 cells, five random fields from each sample, 8,000-10,000 cells per field, were photographed at low magnification (10X) and used for quantitative analysis in Image J. For measuring bead uptake in BMDM cells, the internalization of beads was quantitatively measured by flow cytometry.

The antiviral activity of Stalk Abs was also tested using a complement-dependent cytotoxicity (CDC) assay. In this experiment, MDCK cells were grown in a flat-bottom 96-well plate (Greiner, Cat# 655073) and subsequently infected with various subtypes of the influenza virus at an MOI of 3. After a 12-h infection period, the infected cells were washed twice with PBS. Mouse sera, treated with Receptor Destroying Enzyme (RDE, 1:5 dilution), and mixed with the same volume of 1:5 diluted Guinea pig complement (Sigma, Cat# 234395). The mixtures were added to the wells. To assess nonspecific cell lysis, a control well of cells was treated with 10% complement alone, which established a baseline reaction value. The maximum lysis value was determined by treating the cells with 1% Triton X-100. After 3-h incubation at 37°C, the plates were allowed to sit at RT for 15 min, and the supernatants were subsequently removed. Following this, 100 μl of Opti-MEM media was added to each well, followed by the addition of 100 μl Cell Titer Glo 2.0 luminescent cell viability detection reagent (Promega, Cat# G9241). The plates were incubated at RT for 12 min, and the luminescence was measured using a SpectraMax iD3 plate reader (Molecular Devices). The cytotoxicity resulting from the Ab-complement complex was calculated using the following equation: cytotoxicity (%) = (optical density of baseline reaction - optical density of experimental reaction) / (optical density of baseline reaction - optical density of maximum reaction) x 100.

References:

1. Li W, et al. An FcRn-targeted mucosal vaccine against SARS-CoV-2 infection and transmission. *Nat Commun*. 2023;14(1):7114.

2. Ochsner SP, et al. FcRn-Targeted Mucosal Vaccination against Influenza Virus Infection. *J Immunol*. 2021; 207(5):1310-1321.

3. Tesar DB, et al. Ligand valency affects transcytosis, recycling and intracellular trafficking mediated by the neonatal Fc receptor. *Traffic*. 2006; 7(9):1127-42.

4. Ackerman ME, et al. A robust, high-throughput assay to determine the phagocytic activity of clinical antibody samples. *J Immunol Methods*. 2011; 366(1-2):8-19. Erratum in: *J Immunol Methods*. 2012; 376(1-2):156.

Supplemental Table 1. The characteristics of the recombinant proteins used in the Research.

Protein name	N-terminus	Intermediate	C-terminus (Fc or His)			Binding capacity		
	HA stalk	Fd domain	Hinge region mutations	C1q-binding mutations	FcRn-binding mutations	FcRn	FcγR	C1q
Stalk-Fc/wt	stabilization mutations*	Yes	C224S<br>C227S<br>C229S	E318A<br>K320A<br>K322A	No	+	+	-
Stalk-Fc/mut	stabilization mutations*	Yes	C224S<br>C227S<br>C229S	E318A<br>K320A<br>K322A	HQ/AD(310/311)<br>HN/AQ(433/434)	-	+	-
Stalk	stabilization mutations*	Yes	Fc is replaced with 8x His tag			-	-	-

* Stabilization mutations include: 1) 33 - 310 aa in matured HA protein were replaced with a GSG linker; 2) SIQS sequence before cleavage site (R326/G327) were substituted with QRET sequence; 3) 386-418 aa were replaced with GSGGSG linker; 4) K377M, Y420D, N421L, and E429L mutations were introduced to stabilize the HA Stalk conformation under low-pH conditions

Supplemental Table 2. Groups of animals are immunized with antigens and PBS.

Groups	Antigens	Mice
Stalk-Fc/wt	Stalk-Fc/wt	C57BL/6
Stalk-Fc/wt/KO	Stalk-Fc/wt	FcRn Knock Out (KO)
Stalk-Fc/mut	Stalk-Fc/mut	C57BL/6
Stalk	Stalk	C57BL/6
PBS	PBS	C57BL/6

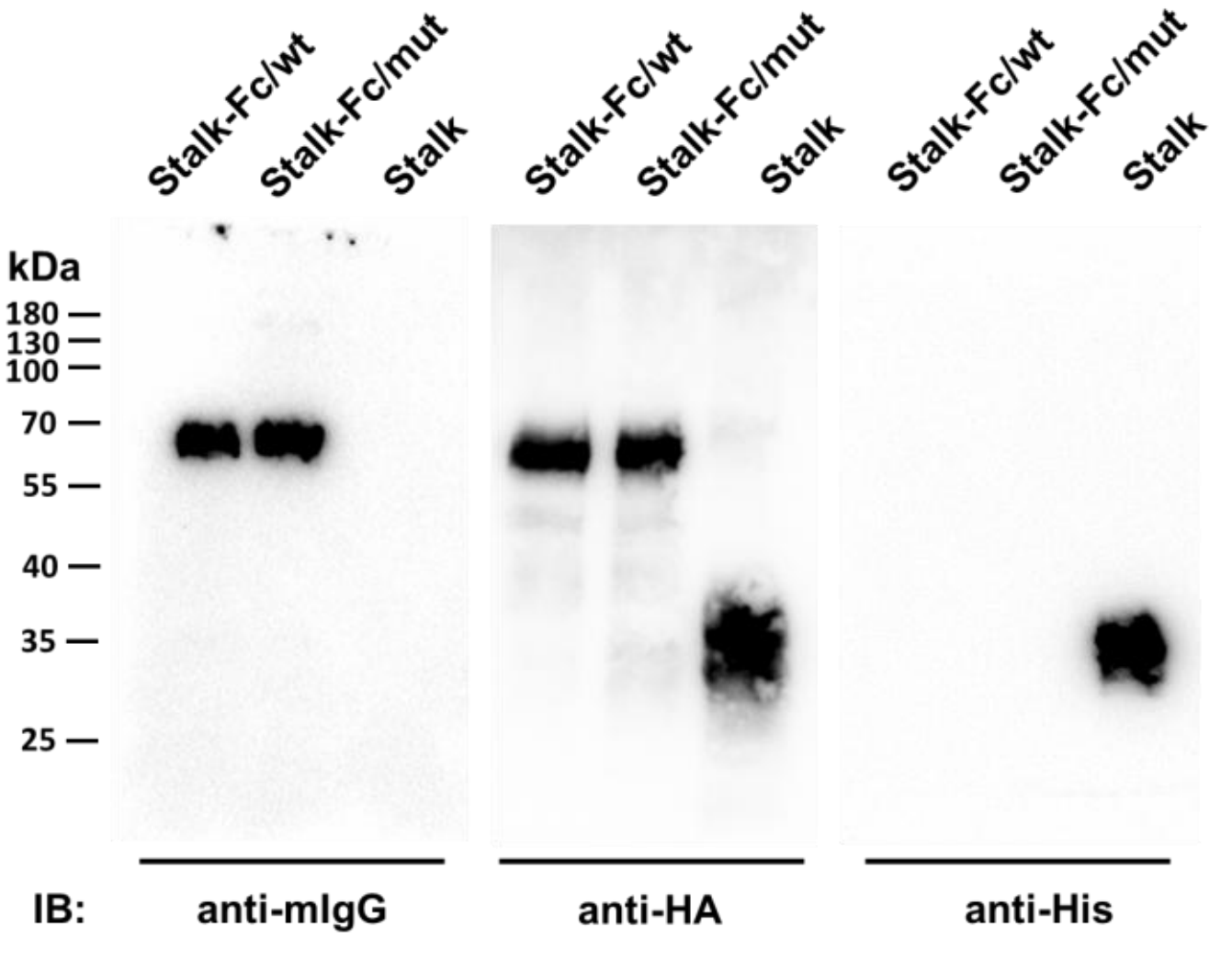

Supplemental Figure 1. Western blot analysis of the Stalk-Fc and Stalk proteins. The purified Stalk-Fc/wt, Stalk-Fc/mut, and Stalk proteins were subjected to 12% SDS-PAGE under non-reducing conditions (left) or reducing conditions (middle and right). The proteins were blotted by HRP-conjugated anti-mouse IgG2a (left), goat anti-HA primary Ab and HRP-conjugated anti-goat Ab (middle), or mouse anti-His Ab (IgG2b) and HRP-conjugated anti-mouse IgG2b (right). The Immobilon chemiluminescent HRP substrate was used to visualize the protein bands.

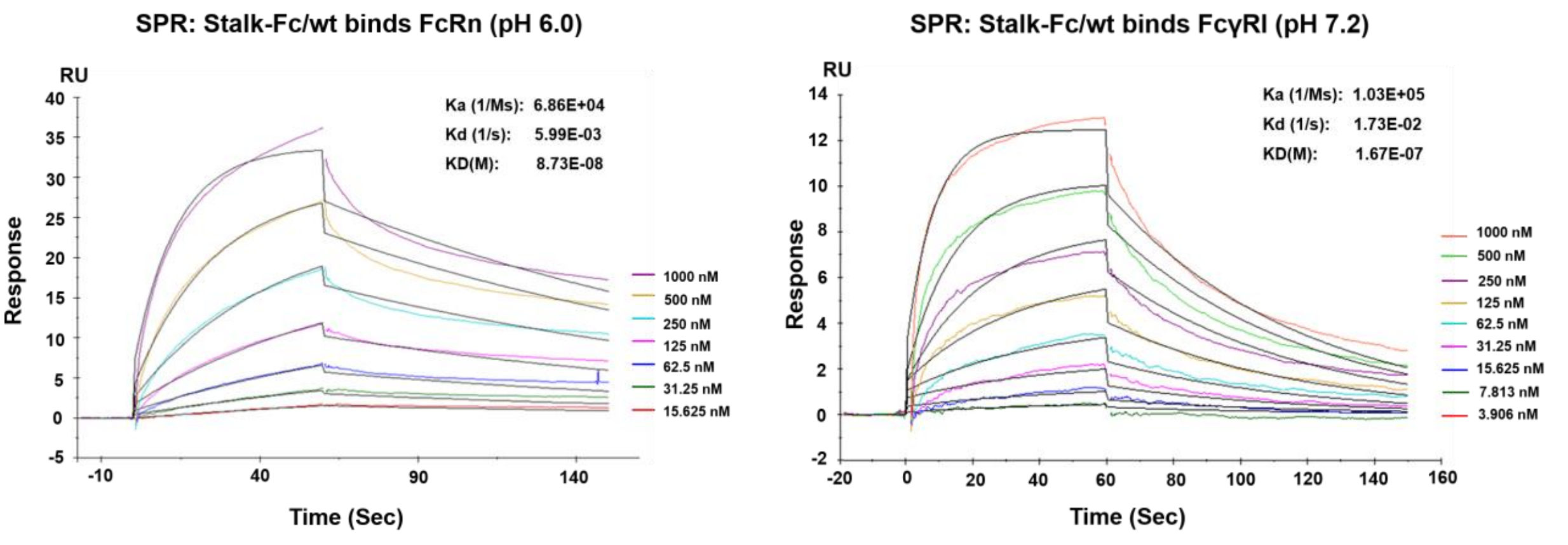

Supplemental Figure 2. Surface Plasmon Resonance (SPR). SPR was utilized to precisely evaluate the affinity of Stalk-Fc/wt binding to mouse FcRn/β2m (left) and mouse FcγRI (CD64) (right).

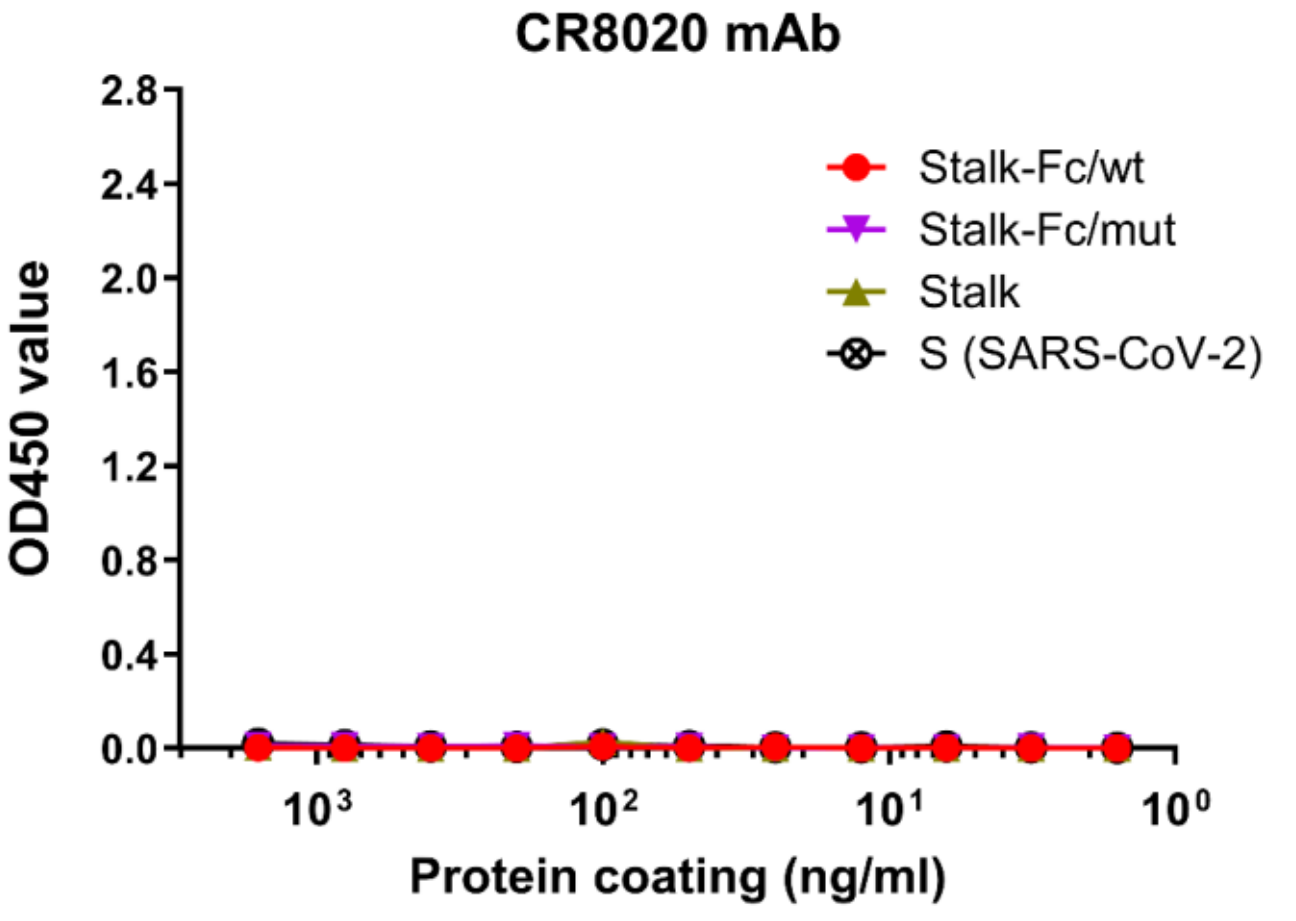

Supplemental Figure 3. CR8020 mAb Binding. Stalk-Fc/wt, Stalk-Fc/mut, and Stalk proteins were coated at different concentrations on the ELISA plate. Spike protein from the SARS-CoV-2 virus served as a negative control. Biotin-conjugated mAb CR8020, which is specific to the HA Stalk of Group 2 influenza viruses, along with HRP-Streptavidin, was used to measure the specific binding.

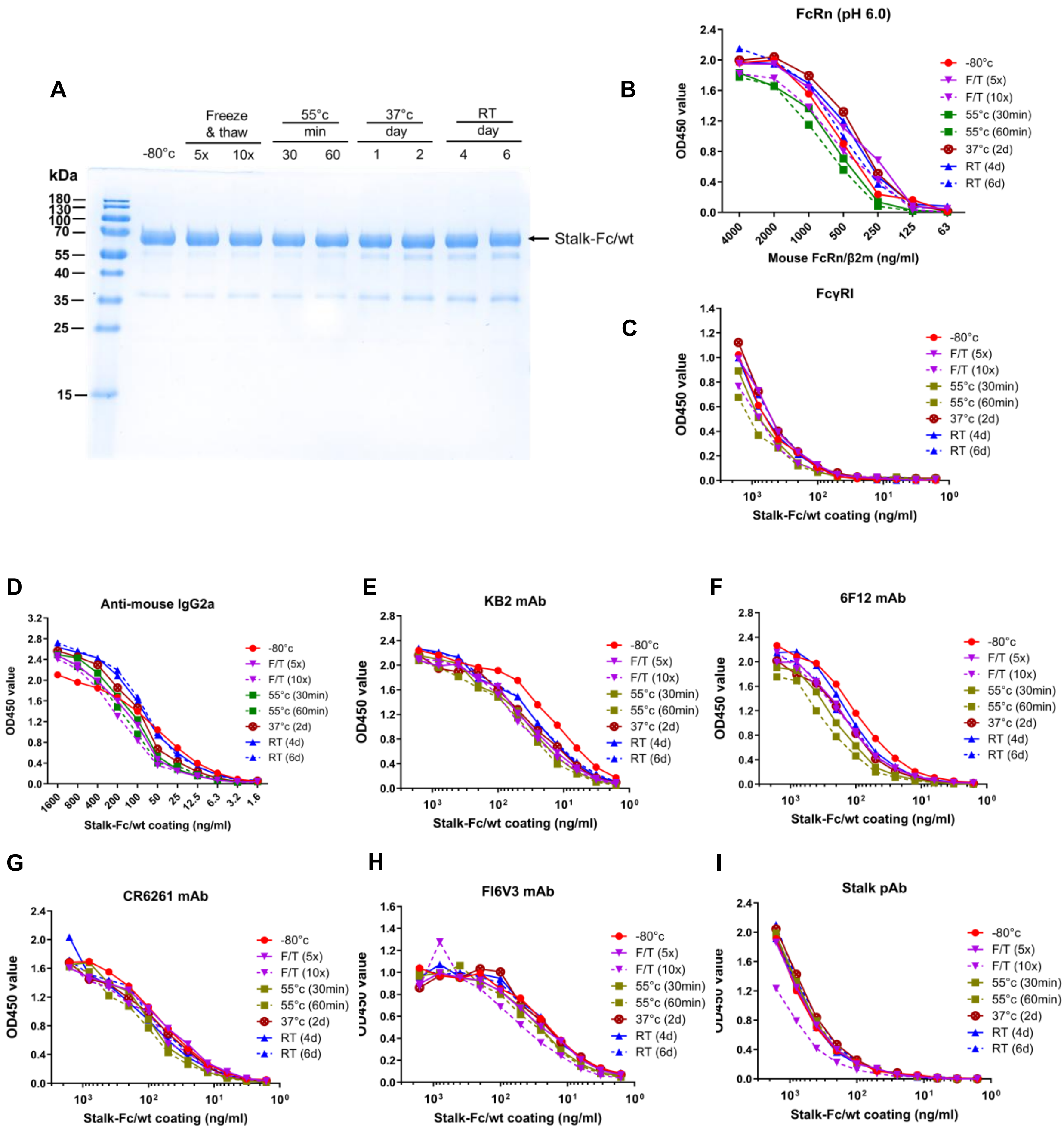

Supplemental Figure 4. Stability of Stalk-Fc/wt protein. Stability of Stalk-Fc/wt protein. (**A**) The Stalk-Fc/wt protein was subjected to various conditions, including freeze/thaw cycles or storage at different temperatures (room temperature (RT), 37°C, and 55°C). After these treatments, the proteins were analyzed using SDS-PAGE under reducing conditions and stained with Coomassie blue. ELISA was performed to evaluate the binding of the treated Stalk-Fc/wt protein to mouse FcRn/β2m (**B**), FcγRI (**C**), anti-mouse IgG2a Ab (**D**), and HA Stalk-specific mAbs KB2 (**E**), 6F12 (**F**), CR6261 (**G**), and FI6V3 (**H**), and a Stalk-specific rabbit polyclonal Ab (**I**).

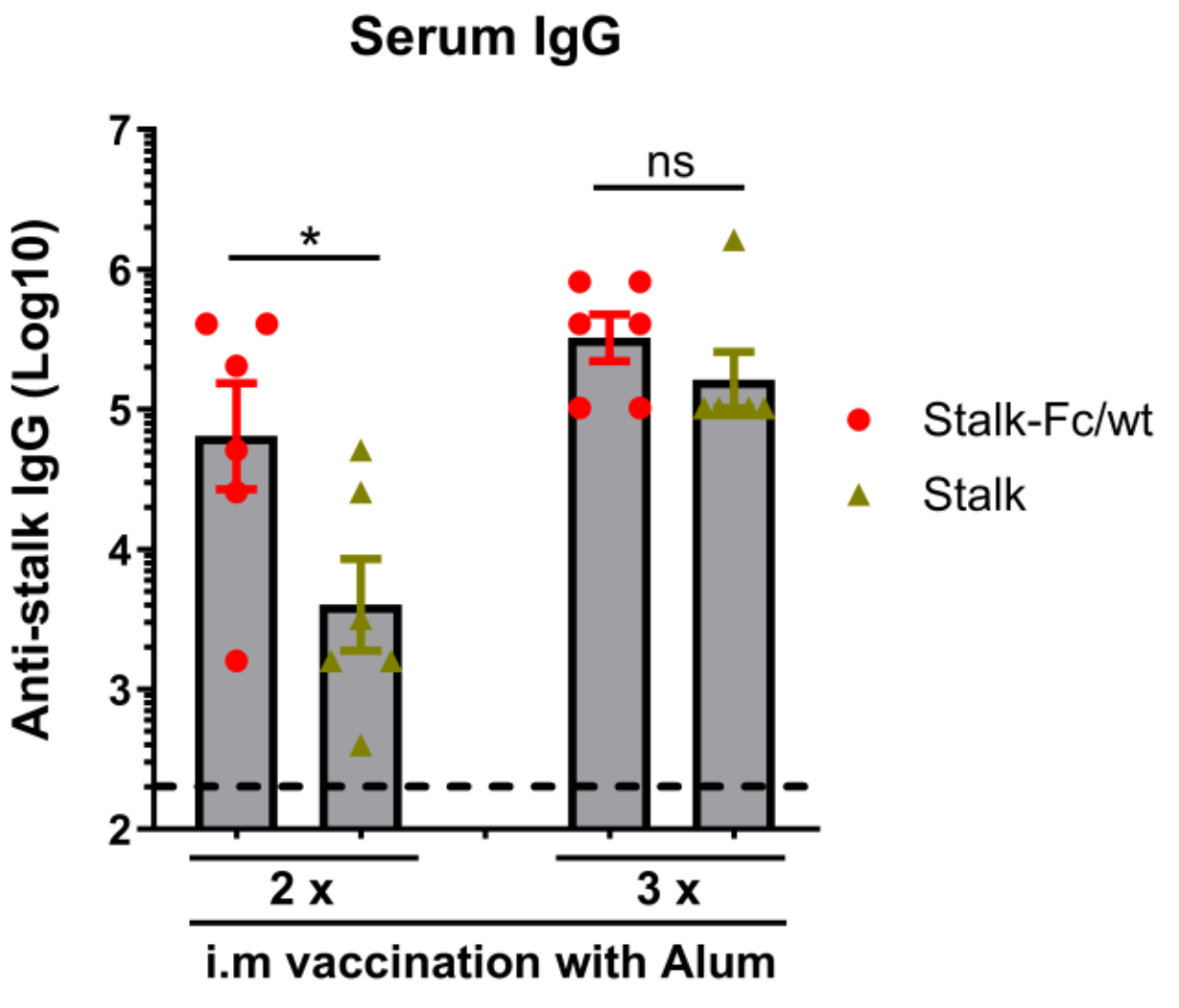

Supplemental Figure 5. Serum antibody responses after intramuscular (i.m.) immunizations with different Stalk antigens. C57BL/6 mice (n=6) at 7 weeks old were i.m. immunized 2 or 3 times with either 10 µg of Stalk-Fc/wt or 5.5 µg of Stalk antigens (equal molar number), combined with Alum adjuvant, at 2-week intervals. Serum Abs were measured using ELISA plates coated with Stalk protein and HRP-conjugated anti-mouse IgG Abs. The Stalk-specific IgG endpoint titer was reported as the mean ± SEM. Statistical analysis was performed using an unpaired t-test for the 2-time immunization group and a Mann-Whitney test for the 3-time immunization group. Asterisks (*) indicate statistical significance (*$p<0.05$), while “ns” denotes no significant difference.

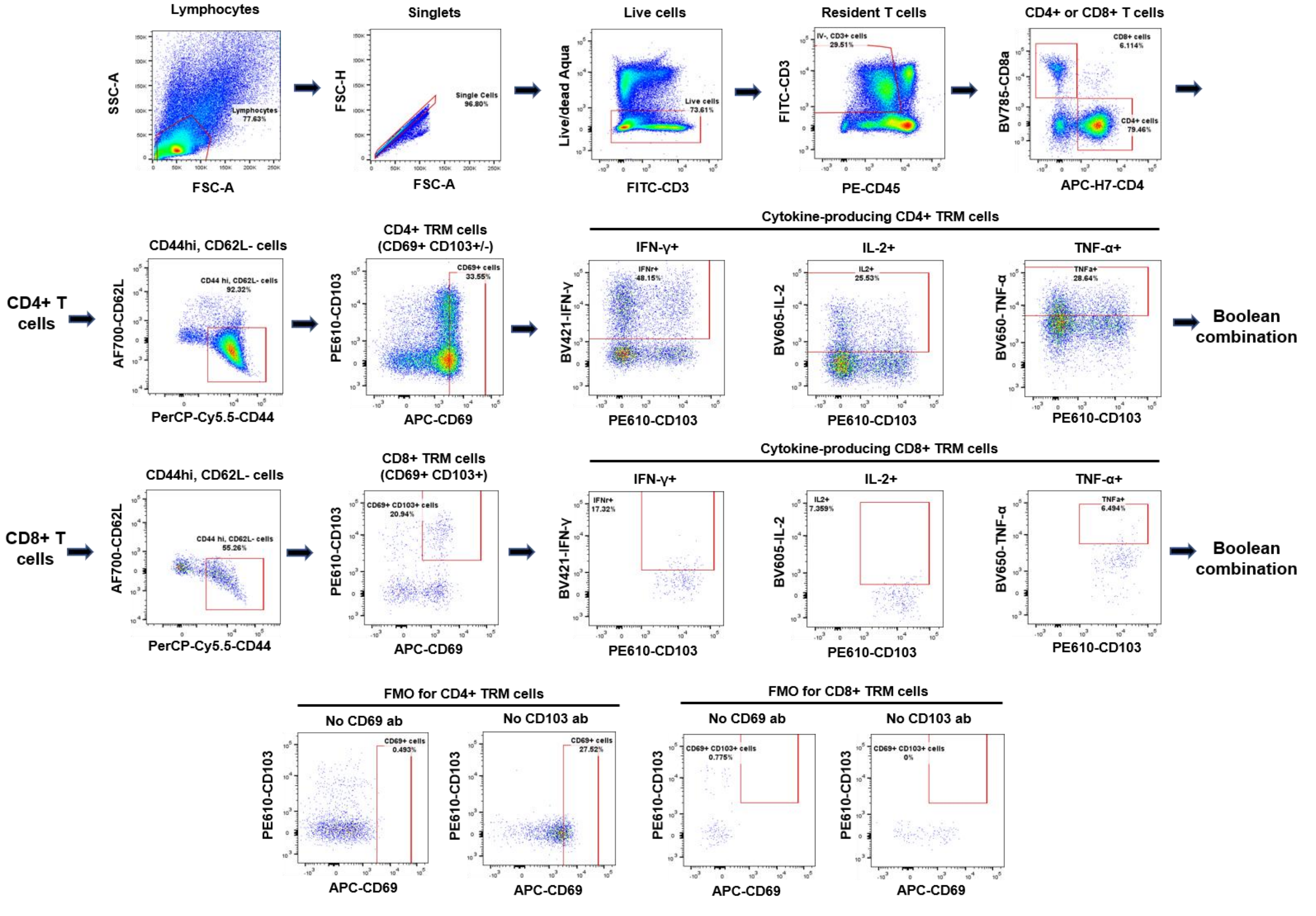

Supplemental Figure 6. Gating strategy for quantifying TRM cells in the lungs. Lymphocytes were gated from lung cells using appropriate FSC and SSC values in the first plot. The single live cells were further isolated through the FSC-A vs FSC-H plot and Live/Dead staining vs FITC-CD3 plot. T cells in the lung were defined as negative for in vivo PE-CD45 Ab staining but positive for in vitro FITC-CD3 Ab staining. Then, this population was divided into two subsets based on the surface expression of CD4 and CD8 molecules. The CD4+ or CD8+ cell subsets with the high expression of CD44 and negative expression of CD62L were further gated for the subsequent staining with APC-CD69 or PE610-CD103 Abs. The CD4+ TRM cells were identified as IV-CD3+CD4+CD44hiCD62L-CD69+CD103+/-, while CD8 TRM cells were identified as IV-CD3+CD8+CD44hiCD62L-CD69+CD103+. To assess the functional state of TRM, three Abs (BV421-IFN-γ, BV650-TNF-α and BV605-IL-2) were used to examine the production of each cytokine in TRM following pooled HA peptide stimulation. The Boolean Combination was applied to characterize the single cytokine-positive TRM cells (monofunctional) as well as the double or triple cytokine-positive TRM cells (polyfunctional). FMO controls without CD69 or CD103 Abs staining for both CD4+ and CD8+ TRM cells were included to ensure the accuracy of gating.

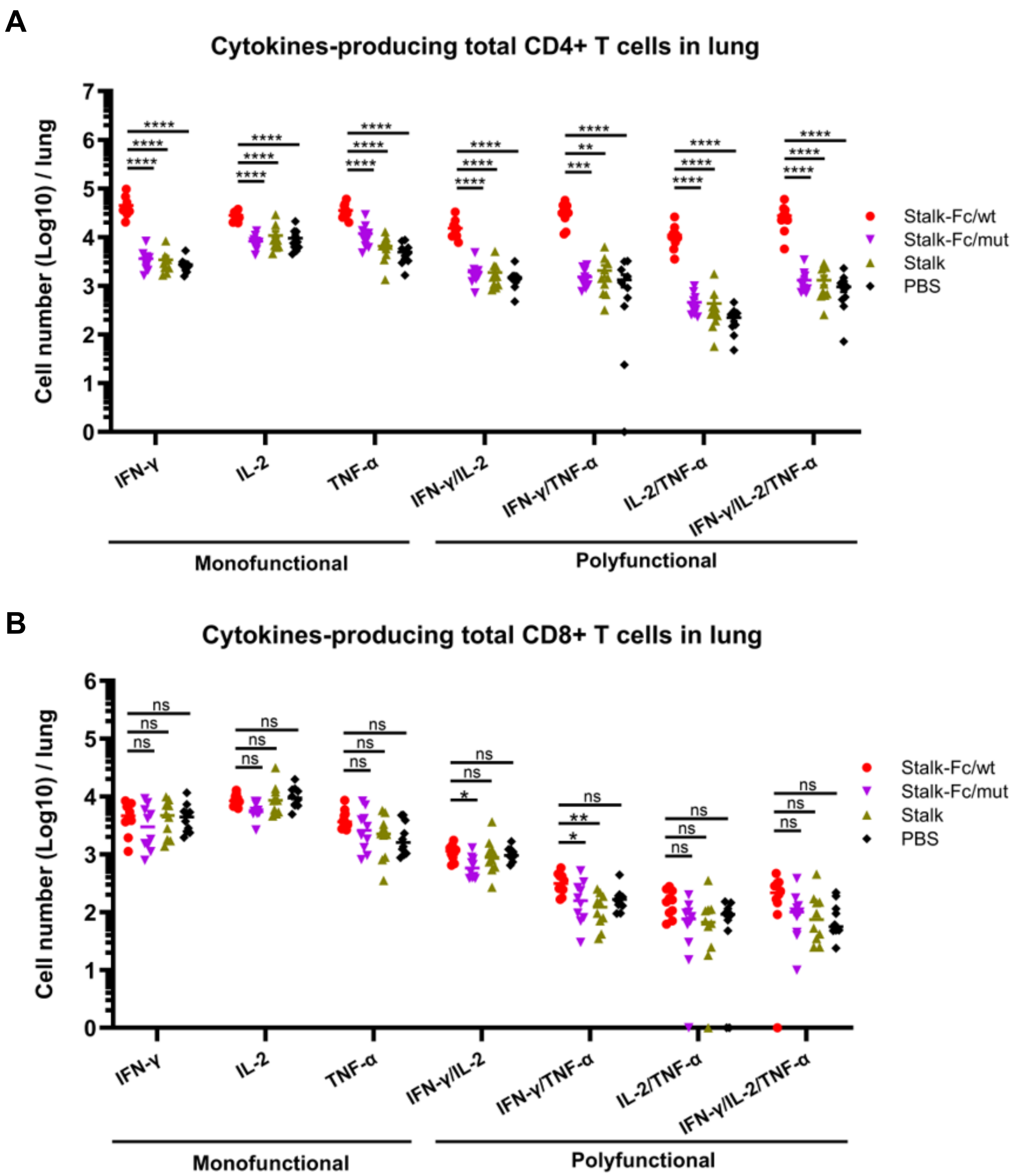

Supplemental Figure 7. Cytokine production was assessed in total T cells isolated from the lungs. The total number of cytokine-producing CD4+ (**A**) and CD8+ T cells (**B**) from each mouse lung is presented as mean ± SEM. The statistical methods used include the Kruskal-Wallis test with Dunn's multiple comparisons for the IFN-γ/TNF-α panel in A, and the IL-2/TNF-α and IFN-γ/IL-2/TNF-α panels in **B**. An Ordinary ANOVA with Dunnett's multiple comparisons test was used for the other panels in both **A** and **B**. Significance levels are indicated as follows: *, $p < 0.05$; **, $p < 0.01$; ***, $p < 0.001$; ****, $p < 0.0001$; and ns, not significant.

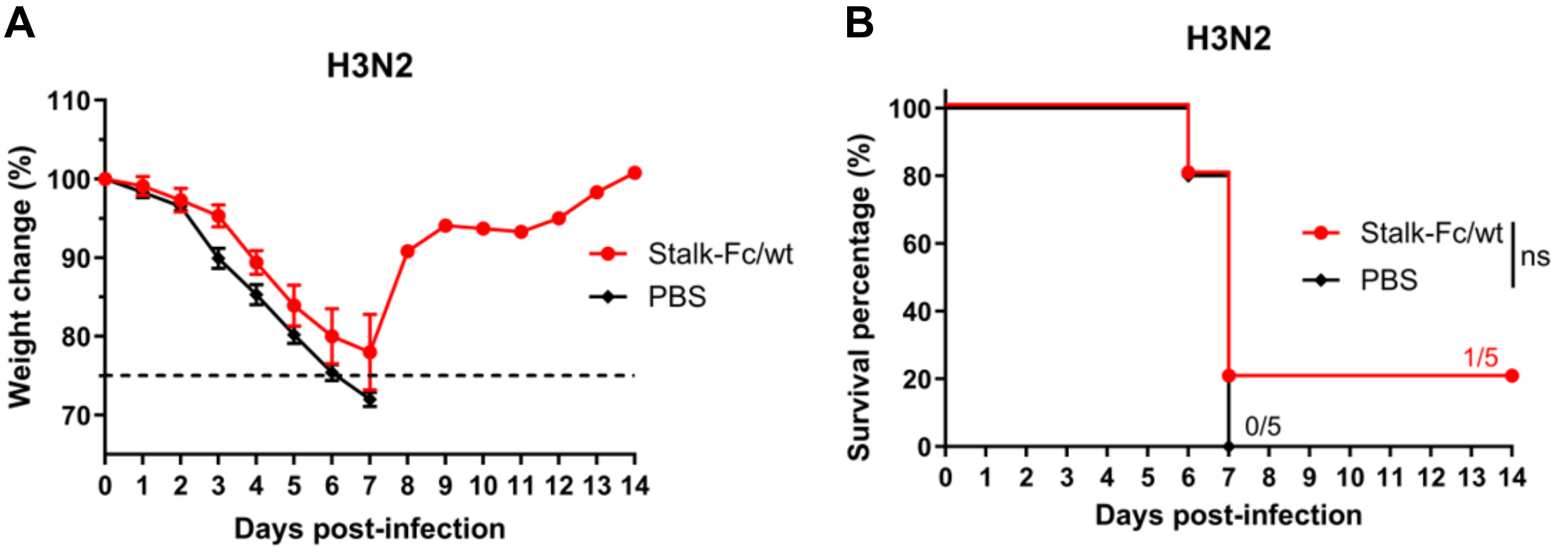

Supplemental Figure 8. Stalk-Fc/wt immunization did not provide protection against the H3N2 virus. C57BL/6 mice (n = 5 group) immunized with either Stalk-Fc/wt or PBS were challenged with the A/Philippines/2/82 X-79 (H3N2) virus at a lethal dose of 5 x 10^3 $TCID_{50}$/50 µl. The results, including daily weight change (mean ± SEM) (**A**) and survival rates (**B**), were presented. The Log-rank (Mantel-Cox) test was employed to assess the statistical differences in the survival rates (**B**). ns, not significant.

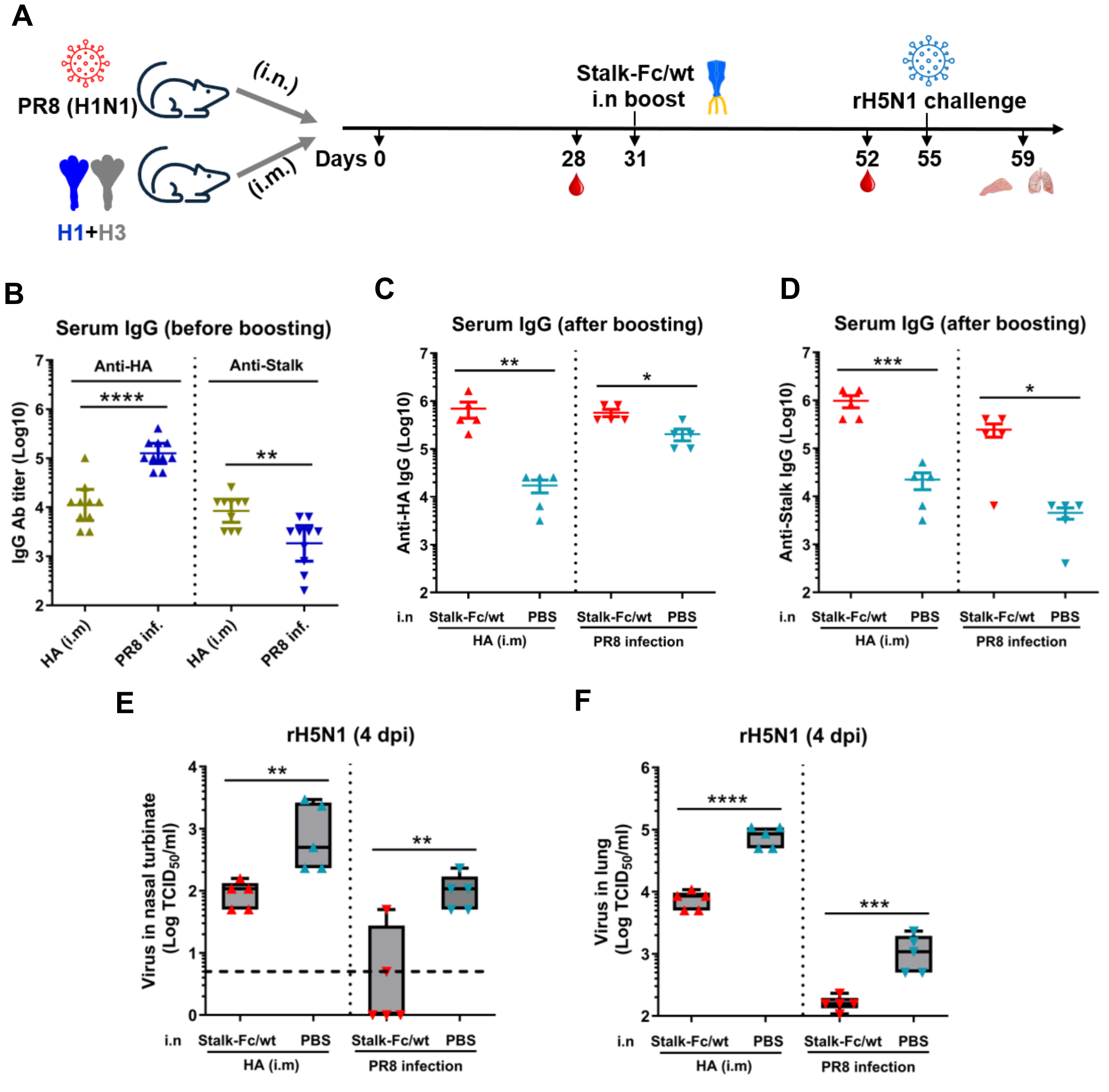

Supplemental Figure 9. Effect of pre-existing Immunity on immune response and protection elicited by Stalk-Fc/wt i. n. immunization. (**A**) The schematic diagram outlines procedures for vaccination, challenge, and sampling. To establish pre-existing immunity against HA, groups of 10 C57BL/6 mice were either i.n. infected with a sublethal dosage of the PR8 virus (0.1 MLD_{50}) or i.m. immunized with 10 μg of HA1 and HA3 proteins, along with adjuvants CpG/Alum. (**B**) Pre-existing immunity was confirmed by ELISA, detecting serum IgG responses specific to either HA1 or Stalk, 28 days after infection or immunization. On day 31, mice of both infected and immunized mice were i.n. boosted with either 10 μg of Stalk-Fc/wt or PBS (n=5 per group). The impact of pre-existing immunity on immune responses boosted by Stalk-Fc/wt was assessed using ELISA to measure HA- (**C**) or Stalk- (**D**) specific serum IgG Ab responses, 21 days after the boost. The effect of pre-existing immunity on protection conferred by the Stalk-Fc/wt booster was evaluated by infecting the i.m. immunized mice with 500 $TCID_{50}$ rH5N1 viruses, and the PR8-infected mice with 1500 $TCID_{50}$, 24 days after the boost (**A**). 4 days post-infection, viral loads in the nasal turbinate (**E**) and lungs (**F**) were measured (mean ± SEM). To determine statistical differences, an unpaired t-test was applied for **B**, **D** (left panel), **E**, and **F**, while the Mann-Whitney test was used for **C** and **D** (right panel). The significance levels are indicated as follows: * $p<0.05$, ** $p<0.01$, *** $p<0.001$, **** $p<0.0001$.

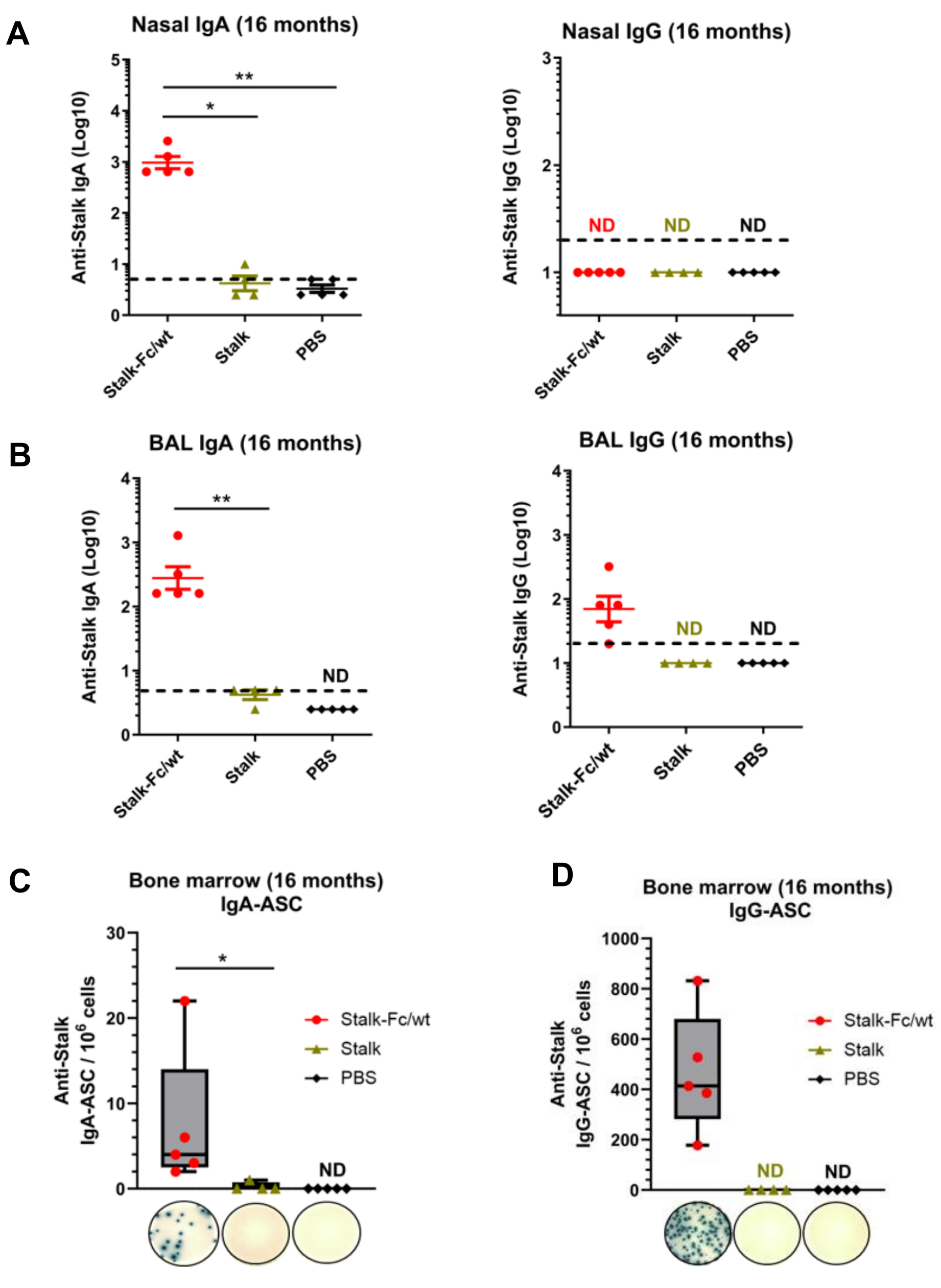

Supplemental Figure 10. Local antibody responses after long-term immunization. The Stalk-specific IgA or IgG Ab responses in nasal washes (**A**) and BAL (**B**) of mice (n = 4-5), 16 months after the final boost with the indicated antigens, were measured by ELISA. The endpoint Ab titers were shown as mean ± SEM. Statistical analyses were performed using the Kruskal-Wallis test followed by Dunn's multiple comparisons for nasal washes (**A**, left), or the Mann-Whitney test for BAL (**B**, left), excluding samples that were not detected (ND). *p<0.05, **p<0.01. (**C** and **D**) Quantification of long-lived antibody-secreting cells (ASC) in bone marrow. Groups of mice (n = 4-5) were i.n. immunized three times with the indicated antigens. After 16 months of immunization, bone marrow cells were isolated and subjected to an ELISpot assay to quantify HA Stalk-specific IgA- (**C**) or IgG- (**D**) ASC cells. The box-and-whisker plots show the median and interquartile range. Cells from the Stalk-Fc/wt group were seeded at 2.5×10^5 cells per well for detecting IgG-ASC, while 1×10^6 cells per well were seeded for detecting IgG-ASC in other groups, as well as IgA-ASC in all groups. Statistical analyses were conducted using the Mann-Whitney test (two-tailed) for (**C**). The analysis did not include ND (not detected) groups. Asterisks (*) indicate statistical significance: *p<0.05.

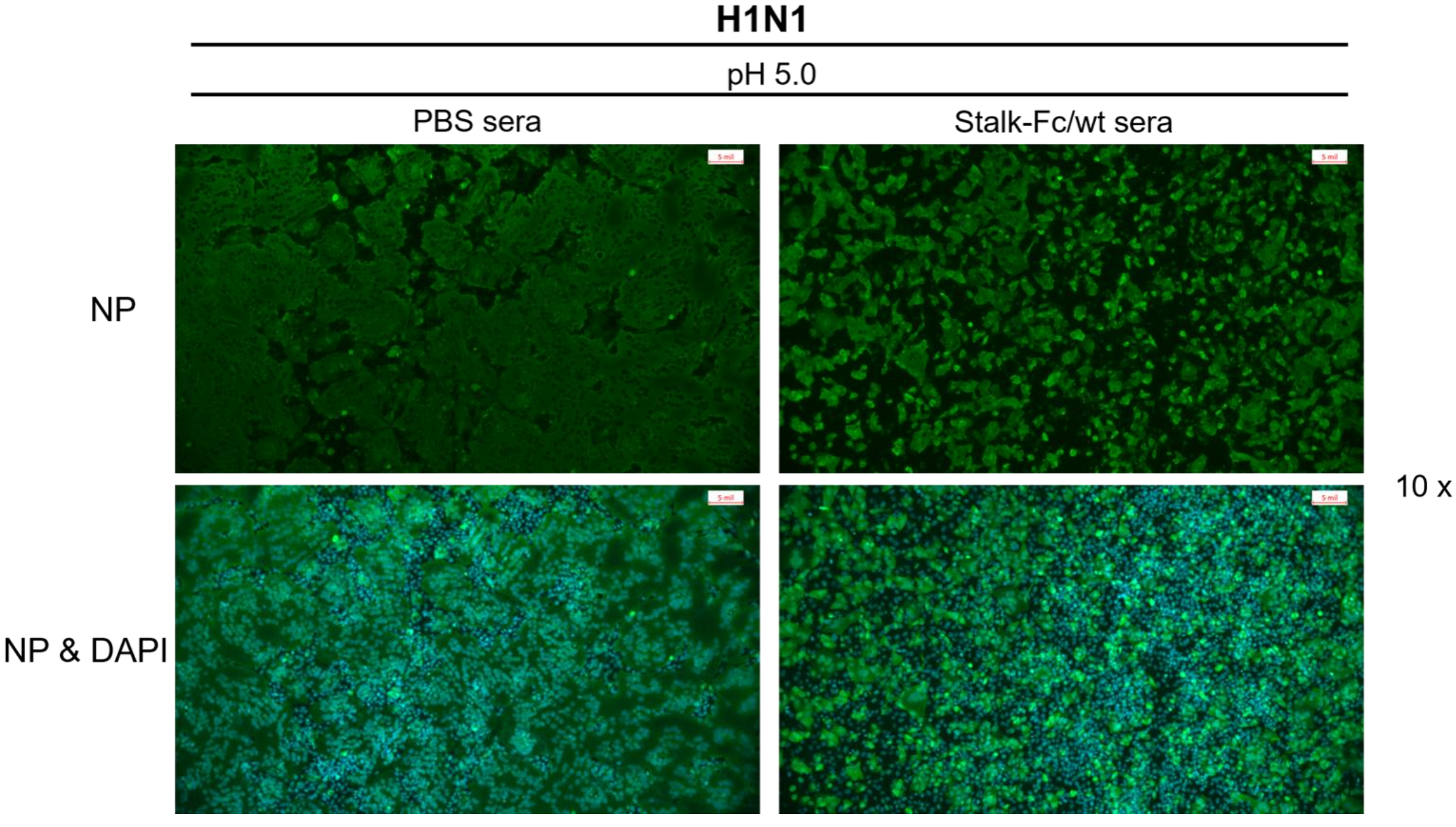

Supplemental Figure 11. Inhibitory effect of immune sera on cell-cell fusion caused by the H1N1 virus infection. Vero cells infected with H1N1 were treated with TPCK-trypsin (5 μg/ml) to cleave HA. Then the cells were incubated for 1.5 h at 37°C with media containing the immune sera from Stalk-Fc/wt or PBS group, followed by a 10-min treatment with a pH 5.0 buffer to induce membrane fusion. After immunofluorescence staining with NP-specific Ab and DAPI, the fluorescent cells were visualized under a Zeiss microscope. The representative fields (10 x magnification) are shown with 127 μm scale bars.

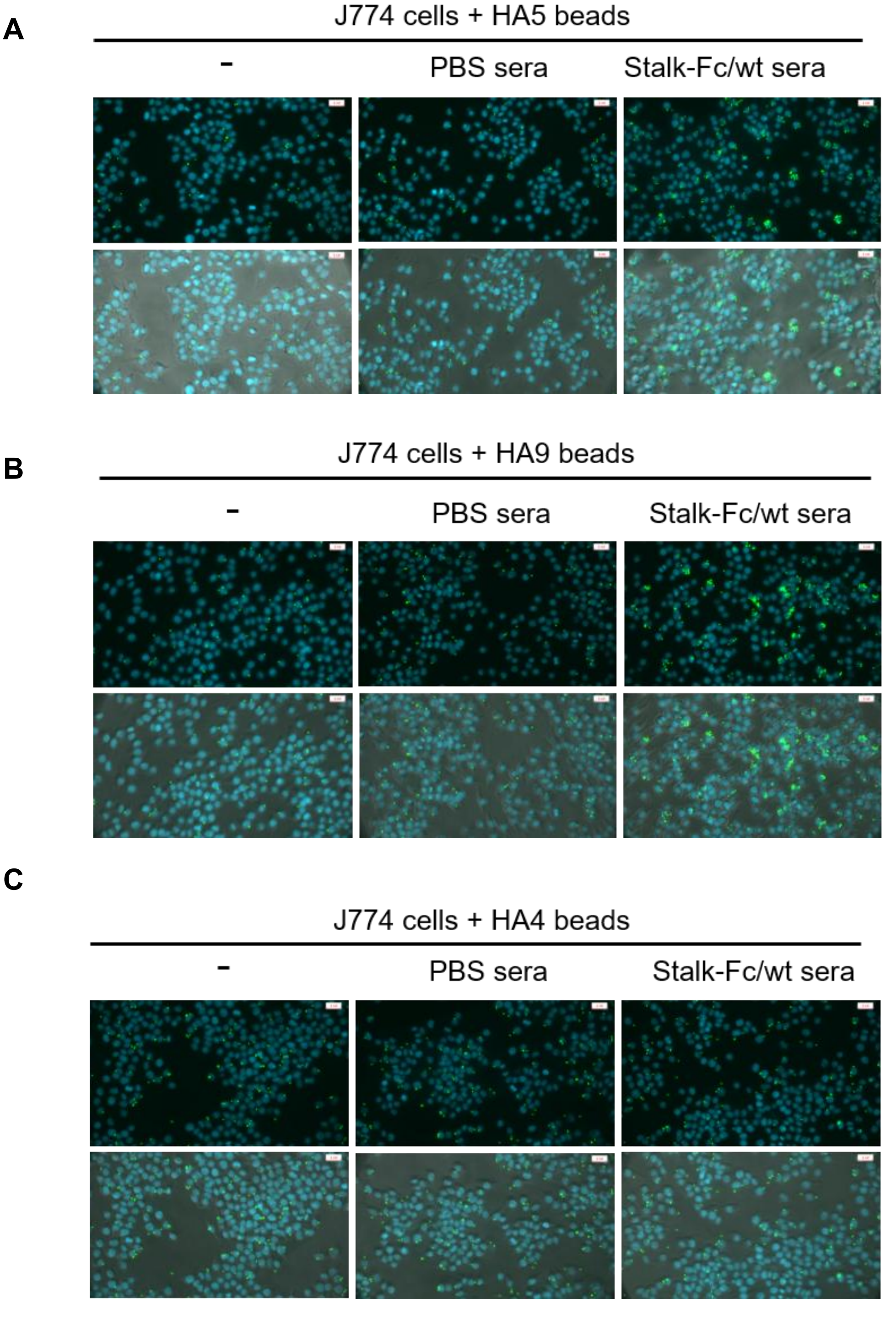

Supplemental Figure 12 . Effect of immune sera on the phagocytosis of influenza HA-coated beads in J774 macrophage cells. NeutrAvidin beads (yellow/green) coated with HA1, HA9, and HA4 proteins from different influenza virus subtypes were incubated with immune sera (10% dilution) from the indicated groups for 2 h. Subsequently, they were transferred to a plate containing J774 cells and incubated for 30 min at 4°C. After washing with PBS, the cells were incubated for an additional hour at 37°C, then fixed and stained with DAPI. The signals of HA1 (**A**), HA9 (**B**), and HA4 (**C**) in the cells were measured using a Zeiss fluorescence microscope. The images were captured with (bottom rows) or without a bright field overlay (top rows). Scale bars: 127 μm.

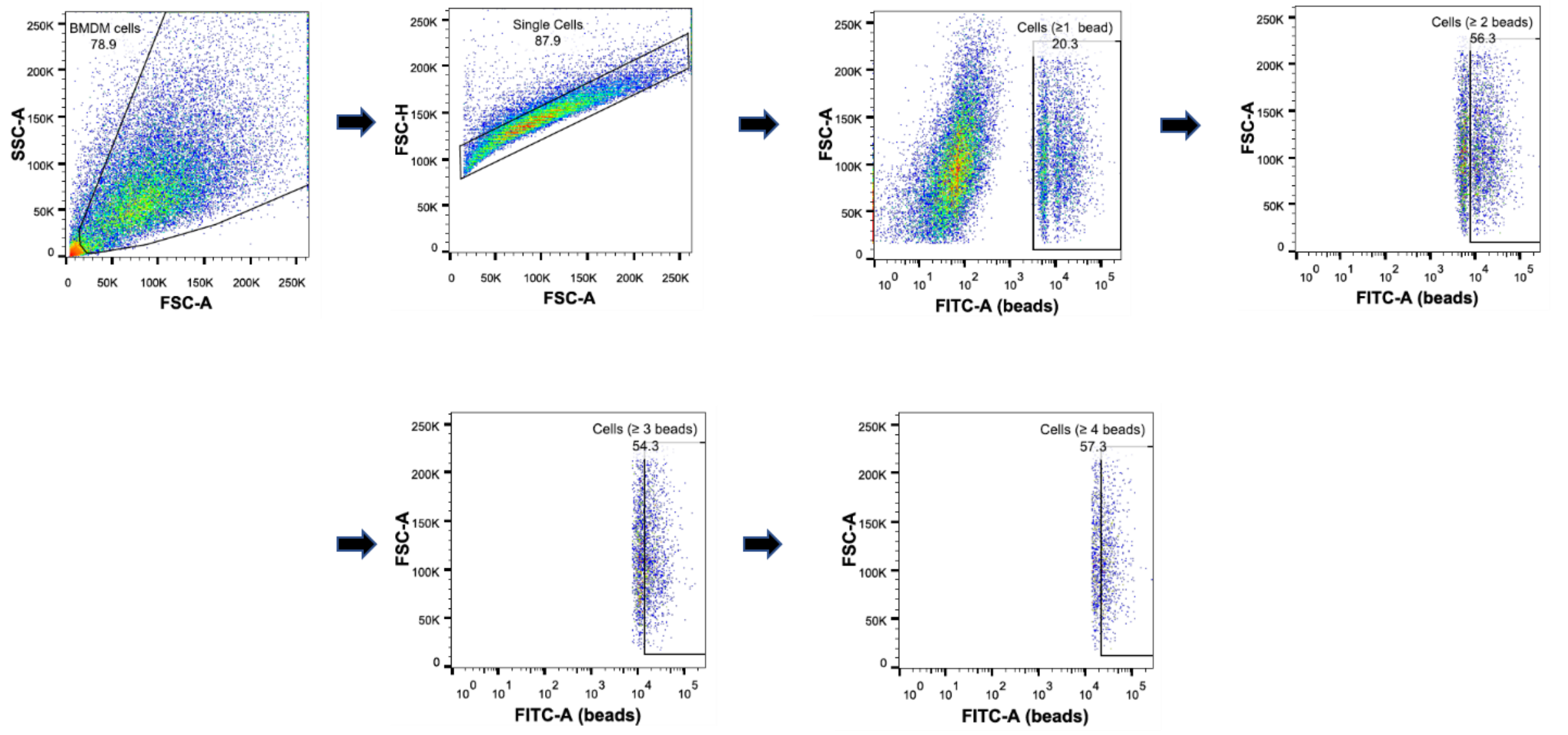

Supplemental Figure 13. Gating hierarchy to quantify the BMDM cells that internalized HA-beads. First, the FSC-A vs SSC-A plot was used to exclude cell debris. Next, the FSC-A vs FSC-H plot was used to identify single cells, which were then separated into two populations based on signals in the FITC channels. Since the number of beads internalized by each BMDM varies, the positive cell's signal intensity also varies. They were further divided into 4 groups, corresponding to the engulfment of more than 1, 2, 3, or 4 beads.

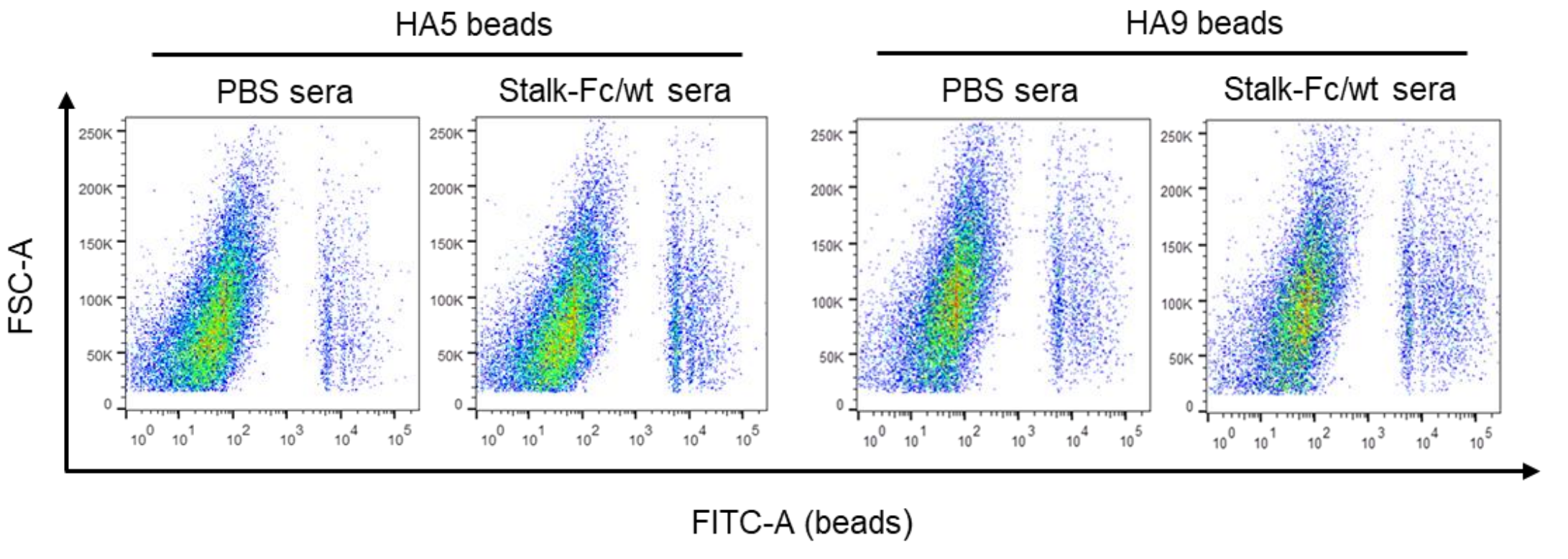

Supplemental Figure 14. Dot plots showing the phagocytosis of HA-coated beads by BMDM cells. The effect of sera from Stalk-Fc/wt immunized or PBS-control mice on the phagocytosis of HA5- (A) or HA9-coated beads (B). The graphs are representative results from two independent experiments.

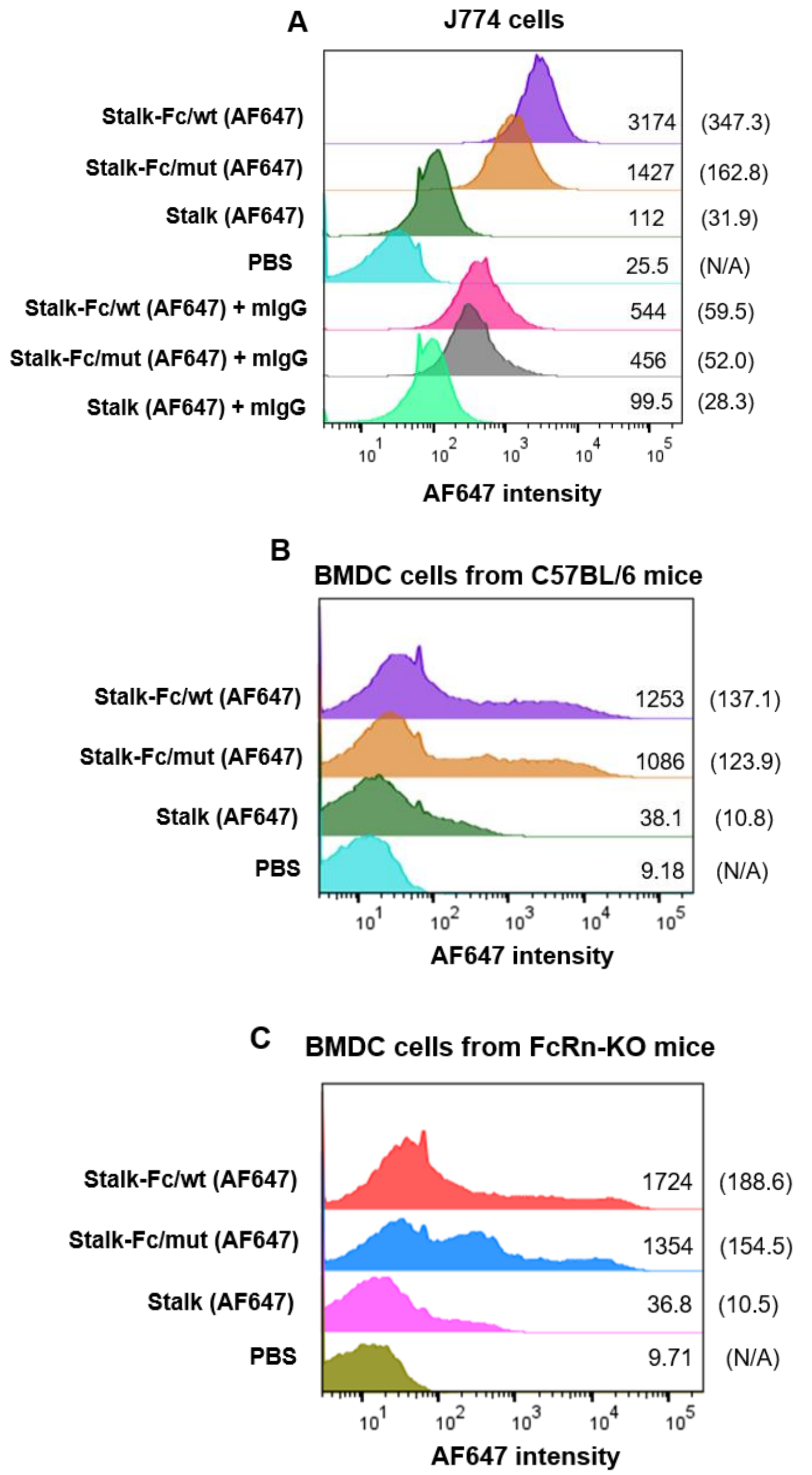

Supplemental Figure 15. Antigen engulfment in J774 (A) or BMDC cells from C57B6/L (B) or FcRn-KO mice (C). J774 cells in a 12-well plate were pretreated or not with mouse IgG at a concentration of 100 μg/ml for 35 min at 37°C. Different AF647-proteins were then added into each well at the same molar dose (equivalent to 1.5 μg AF647-Stalk-Fc/wt protein) without removing the previous media. Cells were incubated at 37 °C for an additional 25 min and stained with Live/Dead aqua for the subsequent flow cytometry assay. BMDC cells from C57BL/6 or FcRn KO mice were seeded into a 96-well V-bottom plate (1.5 x 10^6 cells/well) and incubated for 25 min with AF647-proteins as described above. Then, flow cytometry was conducted after Live/Dead aqua and BV786-CD11c antibody staining. Results were presented as the overlay histograms with MFI (mean fluorescence intensity, number outside the parentheses) and normalized MFI (MFI divided by degree of labeling, number in the parentheses). For both cells, the experiment was repeated twice, and only one representative result was displayed.

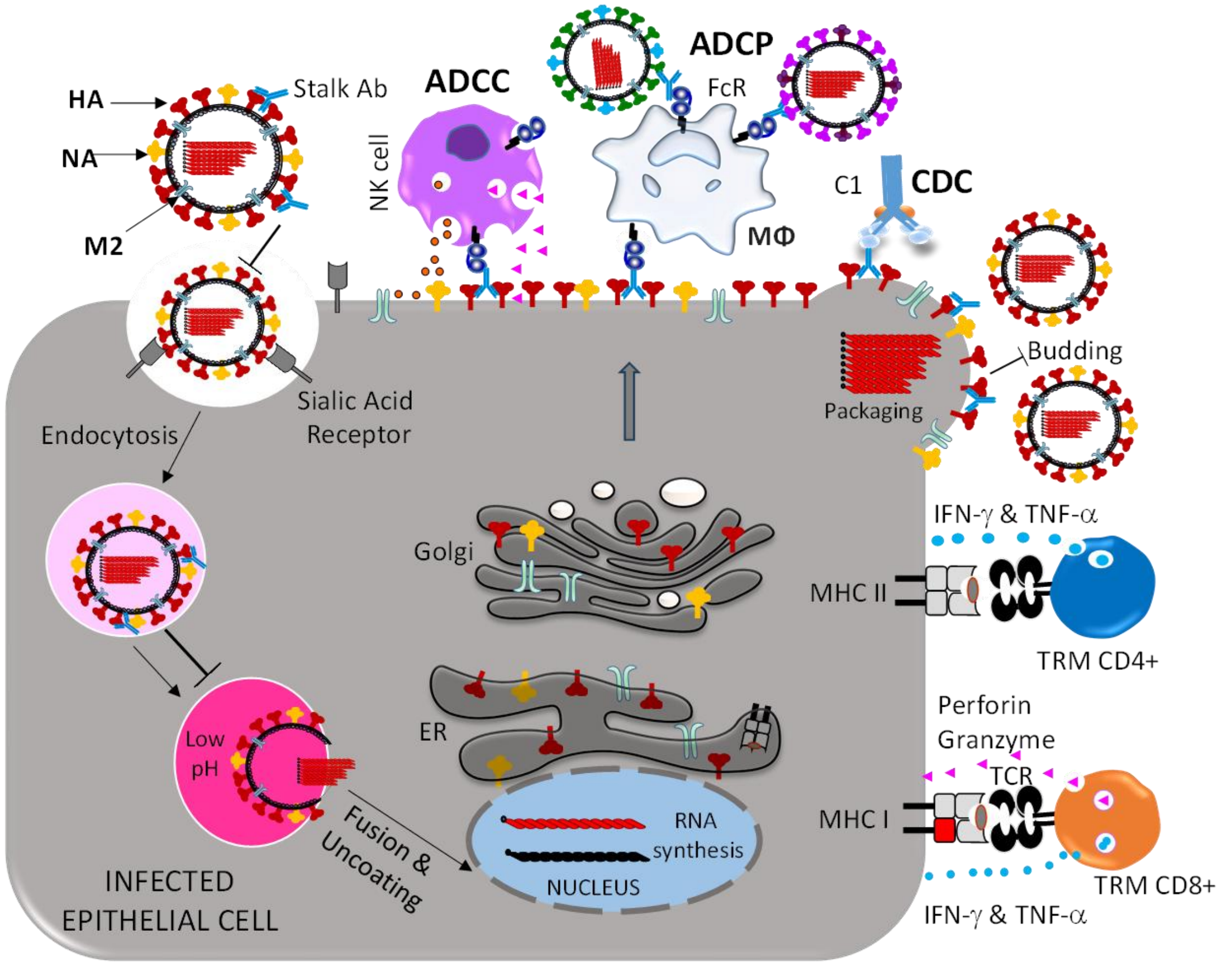

Supplemental Figure 16. The proposed model summarizes the protective mechanisms activated by intranasal immunization with HA Stalk-Fc. HA Stalk-specific Abs neutralize the influenza virus by inhibiting the fusion of the virus with endosome membranes. Additionally, antibodies targeting the HA Stalk proteins can trigger Fc receptor (FcR)-mediated effector functions against infected cells or viruses through indirect mechanisms. These include antibody-dependent cell-mediated cytotoxicity (ADCC), complement-dependent cytotoxicity (CDC), and antibody-dependent cellular phagocytosis (ADCP), which result in the destruction of virus-infected cells or viruses by immune cells or the complement system. Tissue-resident memory (TRM) T lymphocytes in the airways recognize viral peptides presented on MHC II or MHC-I molecules, regulate mucosal immunity by releasing cytokines, and eliminate virus-infected cells by releasing granules, as indicated.
