# Supplementary material for: Mucosal vaccination with hemagglutinin stalk provides broad protection against influenza virus subtypes in mice
Source: J Clin Invest. 2026 Nov 16;136(22):e203675. doi: 10.1172/JCI203675 (PMC13767620; doi:10.1172/JCI203675)

Figure 1B (left panel)

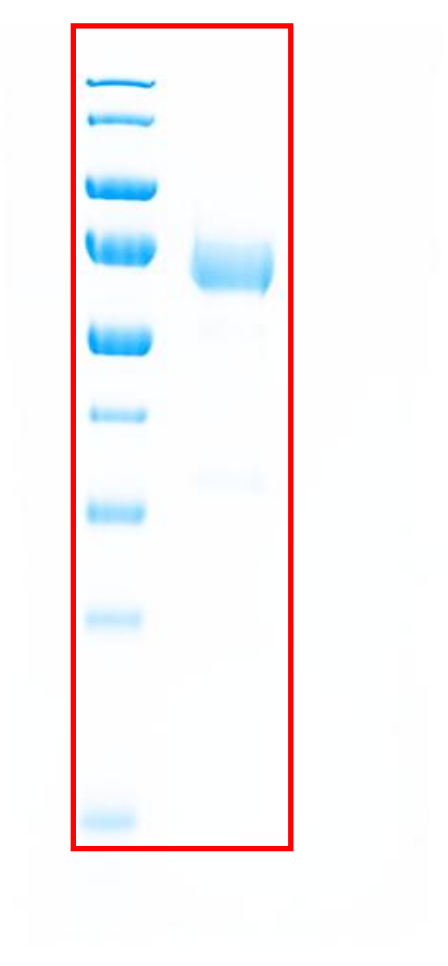

Figure 1B (middle panel)

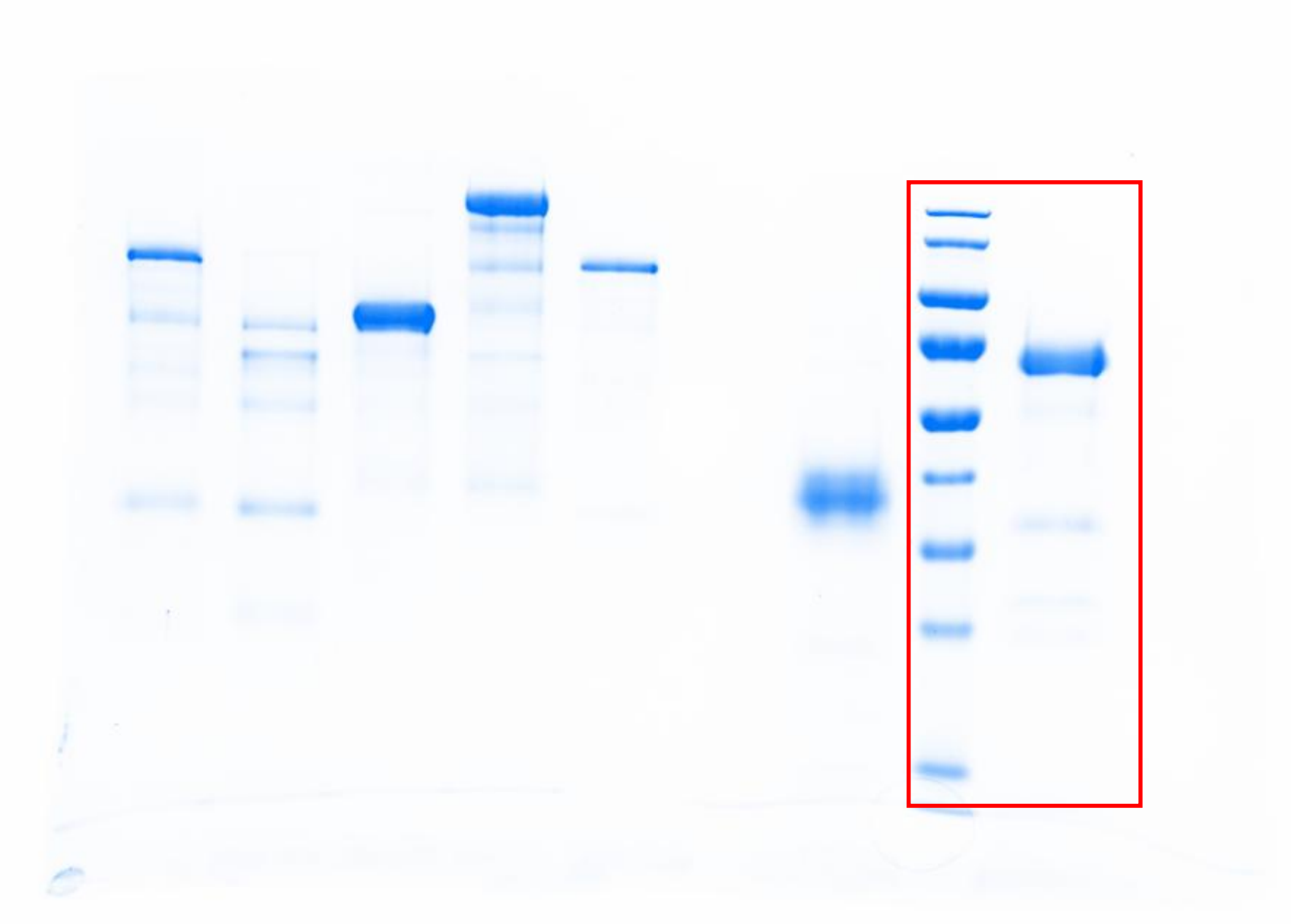

Figure 1B (right panel)

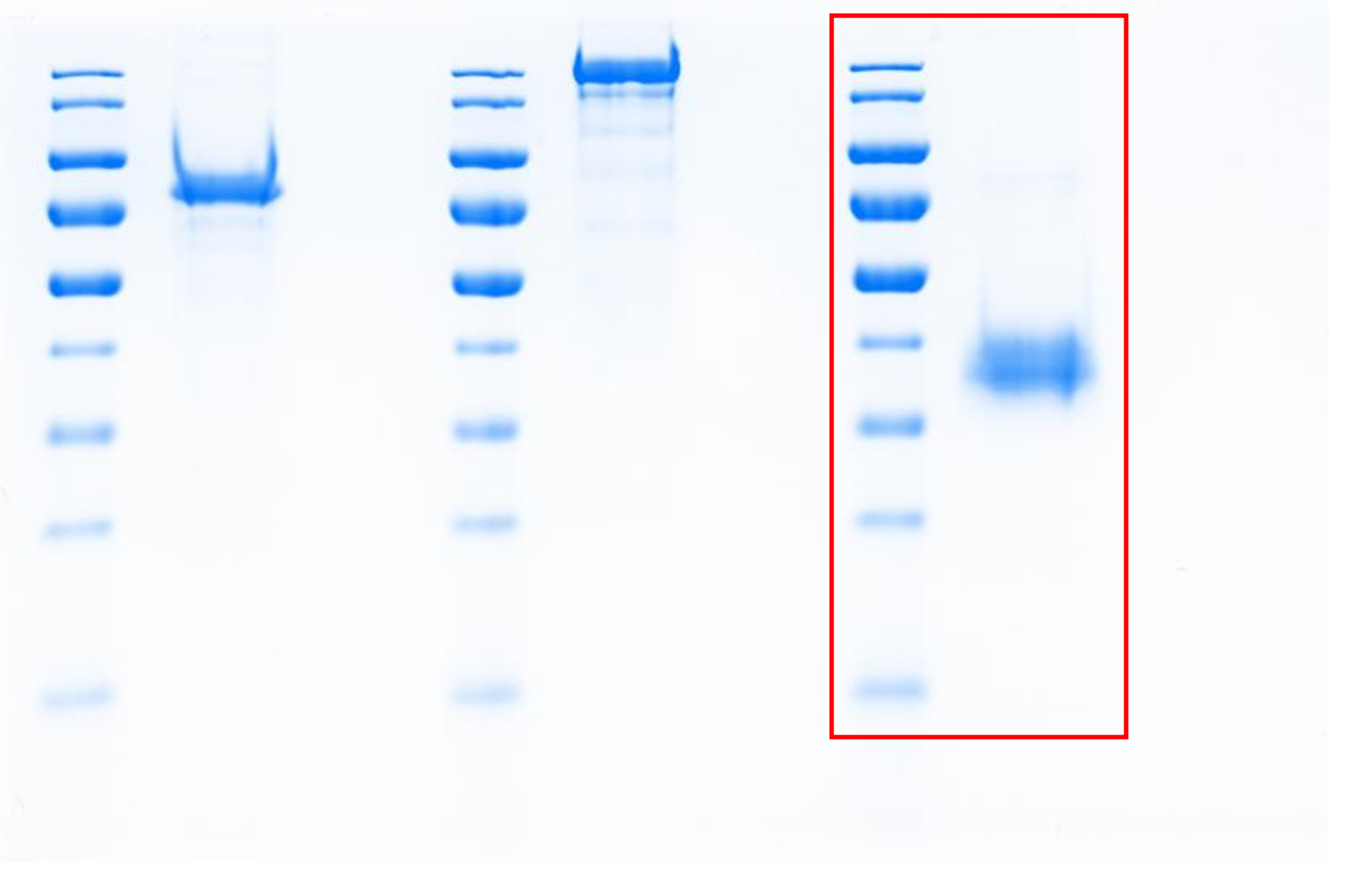

Supplemental Figure 1 (left panel)

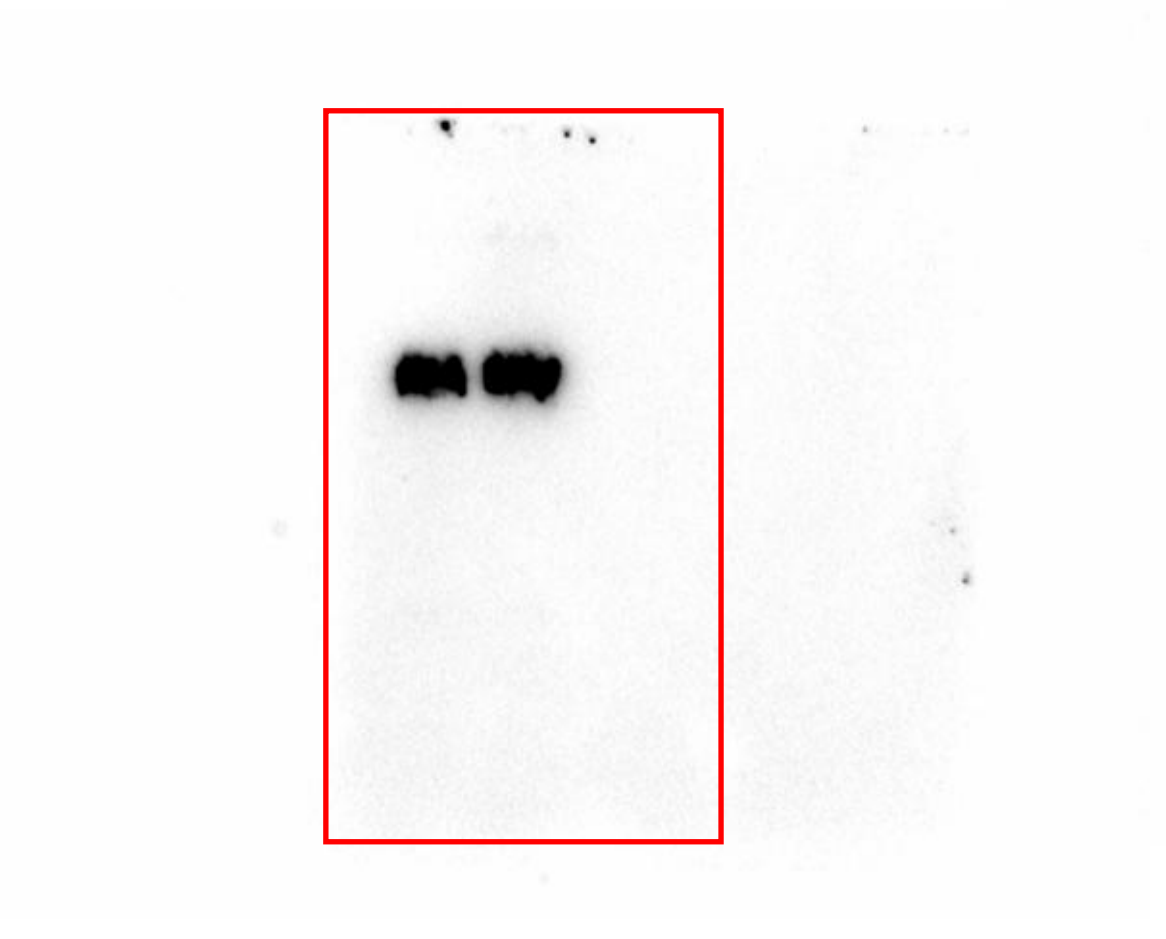

Supplemental Figure 1 (middle panel)

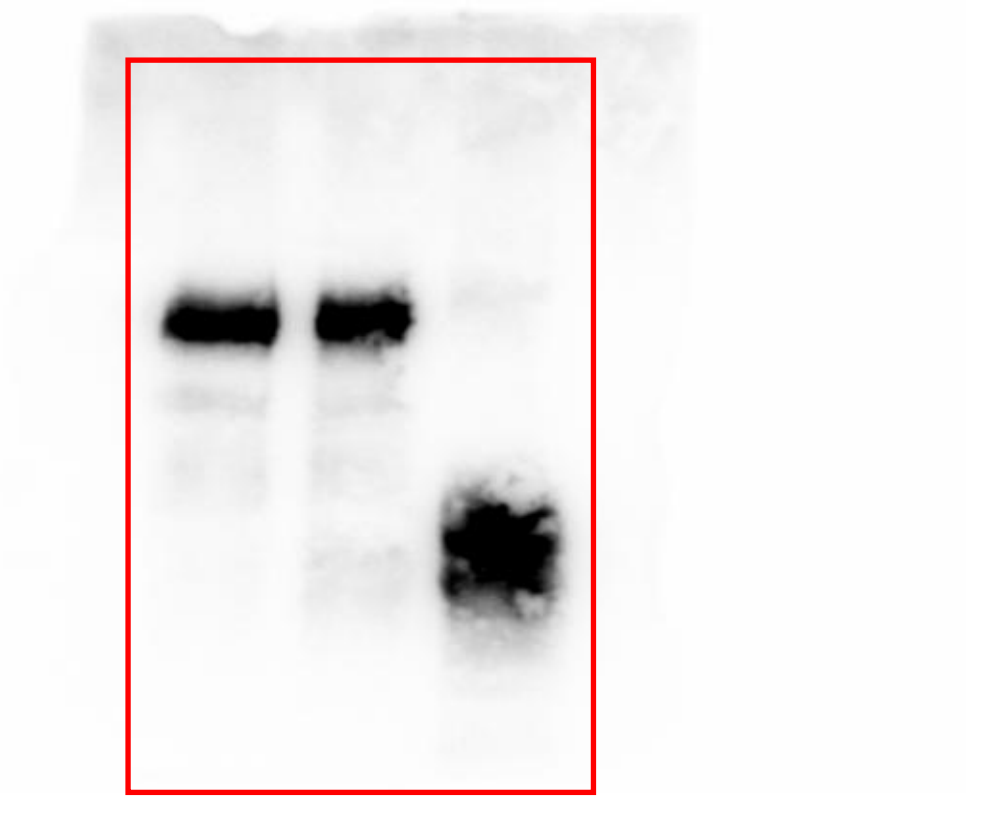

Supplemental Figure 1 (right panel)

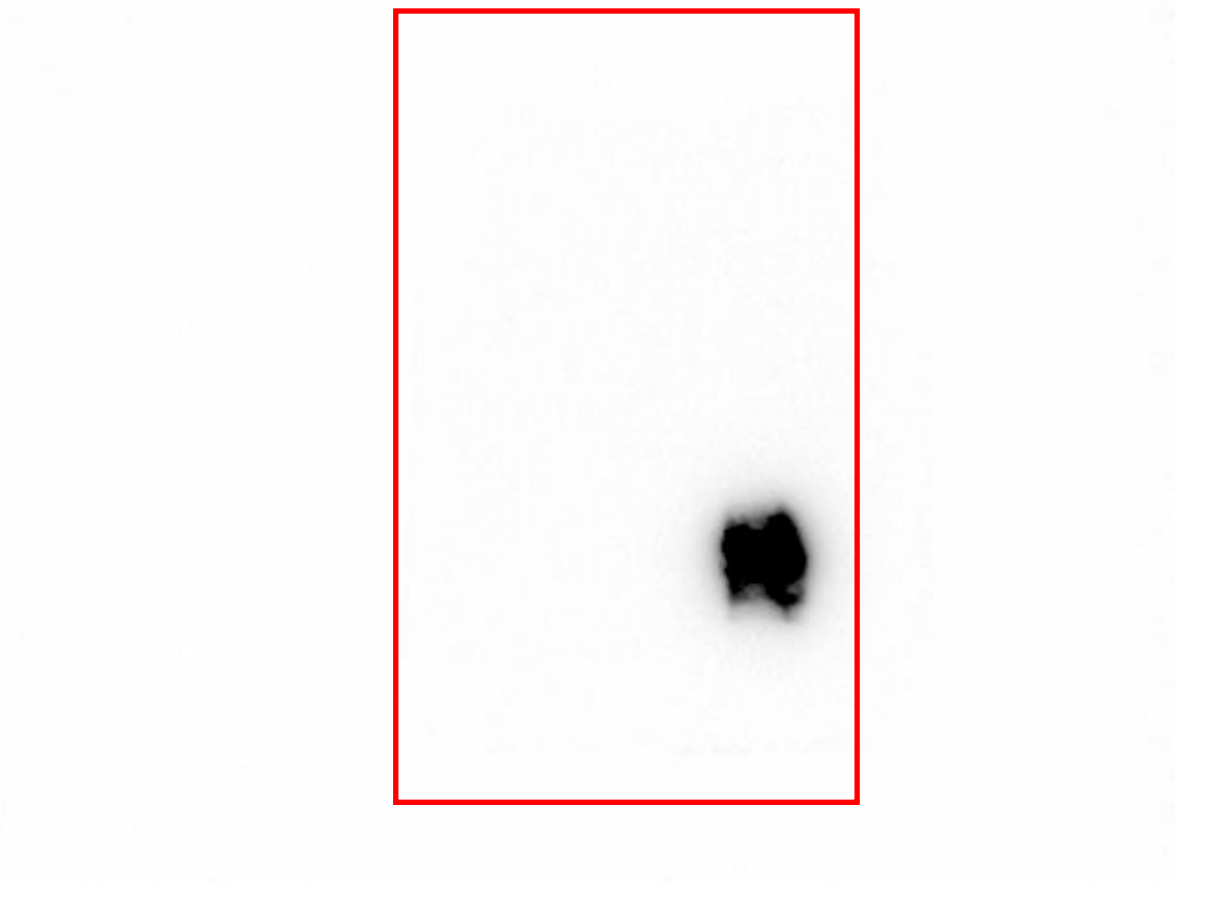

Supplemental Figure 4A

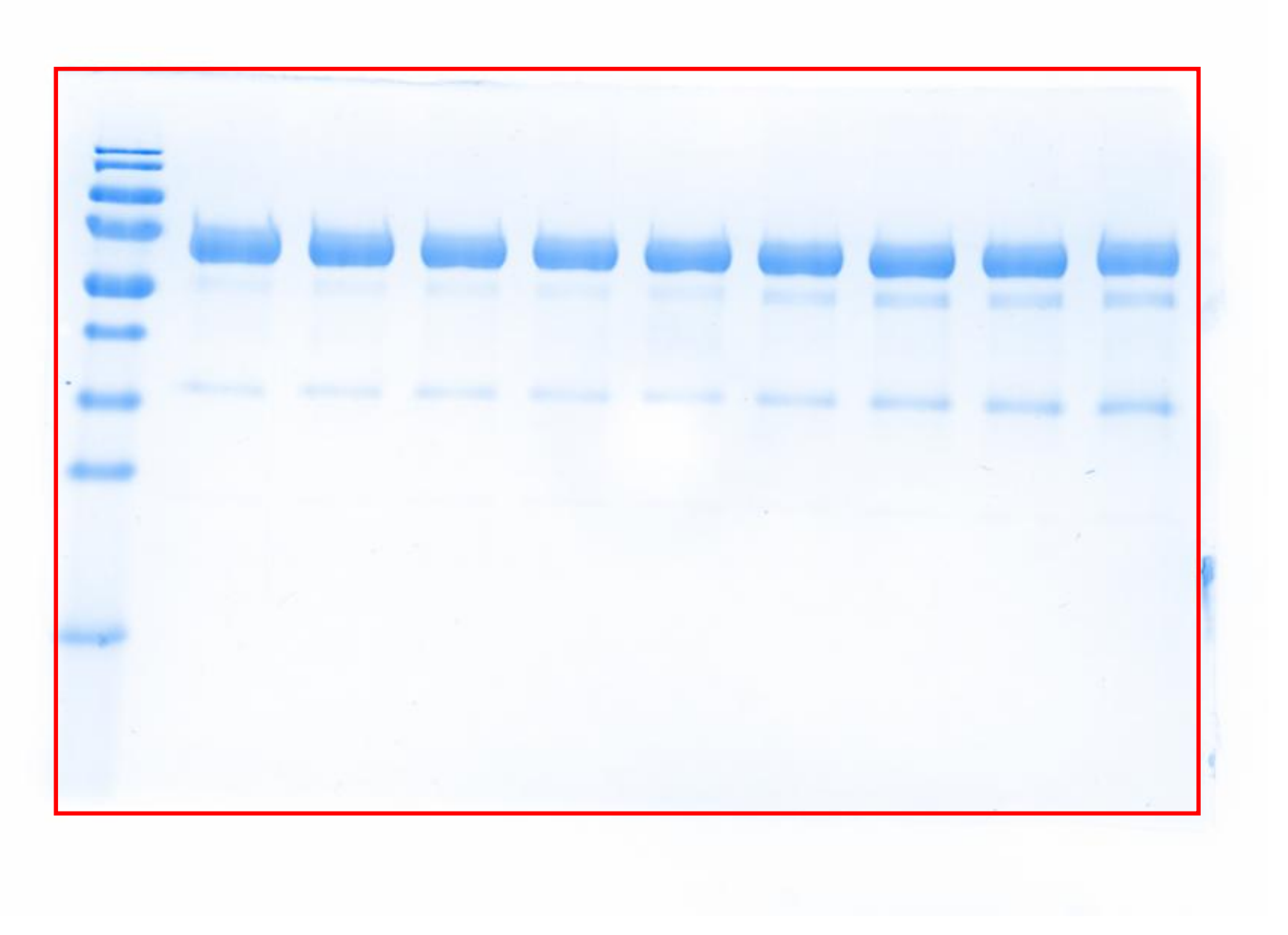

Supplement: Supplementary file 2 [file jci-136-203675-s093.pdf]
